# Bismuth-based nanosystems for precise tumor theranostics: Recent progress, challenges and perspectives

**DOI:** 10.7150/thno.140822

**Published:** 2026-10-02

**Authors:** Haohao Zhang, Mengge Guo, Zhen Kong, Yujie Ma, Laixin Hong, Mingyuan Pang, Zimai Wang, Min Yang, Juan An, Yen Leng Pak, Jibin Song

**Affiliations:** 1 College of Biological and Chemical Engineering, Qilu Institute of Technology, Jinan 250200, China.; 2 College of Chemistry, Beijing University of Chemical Technology, Beijing 10010, China.

**Keywords:** Bi-based nanomaterials, multimodal imaging, synergistic tumor therapy, immune microenvironment regulation, nanotheranostics

## Abstract

Precision tumor diagnosis and treatment are constrained by inaccurate early diagnosis, therapeutic resistance, immunosuppressive tumor microenvironments, and toxic side effects of drugs, resulting in unsatisfactory efficacy of monotherapies. Multifunctional nanoplatforms capable of integrating multimodal imaging, synergistic therapy and immune regulation constitute a core research focus in precision oncology. Bi-based nanomaterials exhibit high X-ray attenuation, tunable band structures, excellent photoacoustic/catalytic performance and controllable degradability, rendering them suitable for imaging-guided precision tumor theranostics. This article systematically reviews four categories of Bi-based nanomaterials: elemental/single-phase, heterostructures, core-shell structures, and Bi-based/containing MOFs. We summarize strategies for structural modification and structure–activity relationship optimization, elaborate their advantages in multimodal imaging, as well as synergistic anti-tumor mechanisms combining phototherapy, radiotherapy, sonodynamic therapy and immunotherapy, including activation of regulated cell death, tumor microenvironment remodeling, and tumor growth inhibition and anti-metastasis effects. Furthermore, the bottlenecks hindering their clinical translation are outlined, and prospects for the development of precision nanotheranostics are proposed. This work provides theoretical references for the clinical translation of Bi-based nanomaterials for tumor theranostics.

## 1. Introduction

Malignant tumors severely threaten human health. Despite advances in surgery, chemotherapy, radiotherapy, immunotherapy and other modalities, clinical tumor diagnosis and treatment still face multiple challenges. Conventional imaging hardly identifies tiny lesions and early metastatic foci, readily leading to missed and misdiagnoses 1, 2. Tumors develop therapeutic resistance via mechanisms including DNA repair and drug efflux. Meanwhile, the immunosuppressive tumor microenvironment (TME) induces effector T cell exhaustion, further impairing therapeutic efficacy 3. Consequently, immune checkpoint inhibitors fail to fully activate systemic anti-tumor immune responses 4. Severe side effects greatly limit clinical translation. Traditional chemotherapeutics lack targeting ability and damage normal tissues; some inorganic nanotheranostics are poorly biodegradable and may cause organ toxicity due to long-term *in vivo* accumulation. These drawbacks greatly hinder safe and efficient clinical diagnosis and treatment 5, 6. Accordingly, developing novel nanotheranostic systems integrating precise visualized diagnosis 7-9, high-efficiency synergistic therapy 10-15 and immune microenvironment regulation 16-20 has become an urgent and promising research direction in oncology.

In recent years, the rapid advancement of nanobiomaterials has offered new strategies to address the aforementioned clinical dilemmas. Among them, Bi-based nanomaterials possess unique and tunable physicochemical properties and exhibit great potential in visual tumor theranostics, whose core advantages can precisely target and alleviate the above clinical challenges (**Figure 1**).

To overcome the deficiency in accurate diagnosis, bismuth has a high atomic number (Z = 83) and excellent X-ray attenuation capability. It can serve as a high-performance computed tomography (CT) contrast agent to markedly improve imaging contrast of lesions. Compared with scarce and high-cost Au- and Pt-based materials, Bi-based nanomaterials feature abundant raw material reserves and low fabrication costs. Such economic and resource merits help cut medical expenditures, overcome the application limitations of noble metal materials, and facilitate clinical translation 21, 22. Au nanoparticles tend to accumulate *in vivo* and carry long-term toxic risks, while Pt-based drugs exhibit severe side effects. Benefiting from well-established clinical application foundations, Bi-based materials can degrade and be metabolized within organisms without heavy metal accumulation. They induce negligible organ toxicity and inflammatory responses, demonstrating superior biocompatibility to Au- and Pt-based nanomaterials at effective therapeutic dosages 23-25.

Outstanding imaging performance constitutes a core advantage of Bi-based nanotheranostics. Bismuth has an atomic number of 83 and a K-edge energy of 90.5 keV, matching the energy window for clinical CT imaging, and possesses stronger X-ray attenuation capacity than gold and platinum. Au (Z = 79) has a lower K-edge energy, and Pt (Z = 78) delivers a weaker X-ray attenuation coefficient. At identical mass concentrations, Bi-based contrast agents achieve higher imaging contrast, facilitating tumor boundary discrimination and precise lesion localization. Furthermore, Bi-based nanomaterials are compatible with multispectral photoacoustic (PA) imaging to realize high-resolution visualization of deep tissues, broadening clinical application scenarios 26-28.

Integrating imaging capability with multiple anti-tumor functions, Bi-based nanomaterials enable the construction of theranostic platforms with greater multifunctionality than Au- and Pt-based counterparts. Certain Bi-based nanomaterials fit the NIR-II theranostic window, possessing deep tissue penetration and high photothermal conversion efficiency for deep-seated tumor treatment. Au nanorods suffer from poor photothermal stability, whereas Pt-based materials lack intrinsic photothermal effects. Owing to its high atomic number, Bi-based materials act as efficient radiosensitizers that generate secondary electrons and ROS to enhance radiotherapy efficacy and mitigate radiation damage to normal tissues, outperforming Au nanoparticles in some scenarios 29. In addition, Bi-based nanocarriers are readily functionalized to load chemotherapeutics and conjugate targeting moieties. They can be combined with sonodynamic therapy, immunotherapy and other modalities to establish multimodal synergistic systems. For instance, bismuthene-based composites achieve synergistic effects of targeted delivery, photothermal therapy (PTT) and immune activation to address the therapeutic bottlenecks of monotherapy 30.

Through structural design, these materials can also be endowed with multimodal imaging functions including magnetic resonance imaging (MRI), PA imaging and ultrasound (US) imaging, enabling precise tumor localization and real-time visual monitoring throughout treatment, and supporting image-guided precision therapy. Aiming at drug resistance caused by monotherapy, Bi-based nanomaterials present multiple therapeutic activities. As a high-Z element, bismuth can achieve efficient radiotherapy sensitization, amplify radiation-induced DNA damage in tumor cells and reverse radioresistance. Additionally, they can generate ROS or local hyperthermia via piezocatalysis, sonocatalysis and photothermal conversion, triggering non-apoptotic cell death pathways such as ferroptosis, pyroptosis and PANoptosis to eliminate tumor cells from multiple dimensions and effectively reverse therapeutic resistance. For the immunosuppressive TME, Bi-based nanomaterials can directly kill tumor cells and simultaneously induce immunogenic cell death (ICD). This process releases damage-associated molecular patterns, promotes dendritic cell maturation and effector T cell infiltration, remodels the immunosuppressive microenvironment, produces an *in situ* tumor vaccine effect, and remarkably improves the response rate of immunotherapy 31-36. In terms of severe toxicity and poor degradability of conventional agents, most Bi-based nanomaterials (e.g., bismuth oxides, bismuth oxyhalides and bismuth chalcogenides) have favorable biocompatibility and controllable degradability. Their *in vivo* metabolic pathways can be optimized via morphology regulation, defect engineering and surface functional modification to reduce the risk of organ accumulation and meet clinical biosafety requirements 37-39.

With the rapid advances in nanosynthesis technology, interface engineering and biomedical research, breakthrough progress has been achieved in Bi-based nanotheranostic systems 40-46. Current research in this field has gradually shifted from the development of single-functional nanomaterials toward multiple research directions, including multifunctional synergistic theranostics, TME responsiveness, precise targeted delivery, and tumor immune regulation (**Figure 2**). Via elaborate design strategies such as nonstoichiometric ratio modulation, heterojunction interface engineering, valence state regulation, and hierarchical structure assembly, researchers have effectively improved the tumor theranostic efficiency, targeting specificity and biosafety of Bi-based nanomaterials, accelerating their translational application in precision tumor theranostics 47-52. Although abundant fundamental research outcomes have been obtained, existing review articles still exhibit evident limitations. Few publications systematically summarize the multifunctional synergistic strategies of Bi-based nanomaterials for visualized tumor theranostics. Summaries regarding the core material design principles, multi-mechanism synergy modes and TME regulation mechanisms remain fragmented and superficial, lacking comprehensive and in-depth systematic induction 53, 54. Most previously published related reviews are limited to a single type of Bi-based material 55-57, individual theranostic modality 58, or merely radiotherapy sensitization effects 59-62, failing to integrate multi-modal theranostics, TME remodeling, immune activation and other multi-dimensional research contents 63. On this basis, grounded on cutting-edge research advances, this review establishes a hierarchical and systematic classification framework for Bi-based nanotheranostic materials, precisely illustrates the structure-activity relationship between material architectures and performances, and deeply analyzes the cascade synergistic mechanisms underlying coupled multi-modal theranostics and immune remodeling of TMEs. Furthermore, forward-looking optimization strategies for clinical translation are proposed by combining emerging interdisciplinary technologies, filling the research gaps in existing reviews.

This article systematically summarizes the multifunctional synergistic applications and state-of-the-art advances of Bi-based nanomaterials in visualized tumor theranostics. First, core material design strategies are summarized from the perspectives of morphology modulation, structural engineering, valence defect construction, interface modification and biosafety optimization. Second, their application merits in multi-modal imaging including CT, MRI, PA imaging and US imaging, as well as the value of imaging-guided precision theranostics are elaborated. Meanwhile, the action mechanisms of radiotherapy sensitization, photodynamic therapy (PDT), sonodynamic therapy and immunotherapy are comprehensively dissected, with emphasis on summarizing the latest research breakthroughs of multi-modal synergistic theranostics, TME-responsive cascade catalysis, and closed-loop theranostic systems. Finally, targeting the development goals of intelligence, precision and clinical translatability, the future research directions and interdisciplinary innovation trends of such nanotheranostic platforms are prospected. This review aims to sort out cutting-edge achievements in this field, refine the theoretical system of Bi-based precision tumor theranostics, provide comprehensive and authoritative references for relevant investigations, and promote the deepening of fundamental research and industrial translational development of Bi-based nanomaterials toward clinical precision tumor treatment.

## 2. Design and Synthesis Strategies of Bi-based Nanomaterials

The pursuit of precise and synergistic tumor theranostics necessitates the development of multifunctional nanotheranostic platforms. Benefiting from high X-ray attenuation, excellent photothermal/photodynamic conversion efficiency, favorable biocompatibility, and tunable catalytic activity, Bi-based nanomaterials possess unique advantages for multimodal tumor theranostics. Their overall theranostic performance, targeting capability, and biosafety are closely governed by structure–activity relationships regarding morphology, defect/valence states, interfacial properties, and biocompatibility design. Accordingly, rational structural modulation and precise synthetic strategies are essential to realize synergistic multifunctionality and accelerate the clinical translation of Bi-based nanosystems. To fully exploit their theranostic potential and resolve the inherent drawbacks of conventional tumor therapy-including unsatisfactory therapeutic outcomes, severe systemic toxicity, and the dissociation of diagnosis and treatment-multidimensional optimization strategies have been developed, including targeted structural engineering, precise defect and valence regulation, heterojunction and interface modification, as well as the tailored improvement of biocompatibility and degradability.

In terms of material structures, Bi-based nanomaterials are classified into four categories: elemental/multimetallic composite Bi-based nanomaterials, non-heterojunction core-shell coated composites, typical Bi-based heterojunction nanomaterials, and Bi-based/containing metal-organic framework nanomaterials (Figure 3). **Table 1** summarizes the core positioning, prominent structural characteristics and core advantages of each category.

### 2.1 Elemental/Multimetallic Composite Bi-based Nanomaterials

As the most fundamental Bi-based nanostructures, this category generally includes metallic bismuth 71, Bi-based oxygen-containing compounds 72-74, bismuth fluoride (BiF_3_) 64, 75, Bi-based multimetallic composites 76, Bi-based perovskites 65, 77 (**Table 2**). From the crystal structure perspective, these materials are mostly single-phase crystals, or multimetallic composite Bi-based nanomaterials with simple, regular structures and no complex heterointerface defects, delivering high crystallinity and chemical stability. Their tunable electronic and band structures, narrow band gap, abundant defects and oxygen vacancies endow them with versatile properties including photothermal effect, acoustic response, catalytic activity and radiosensitization. Monocomponent crystals display obvious anisotropic growth. Various morphologies (nanoparticles, nanoflowers, nanosheets, nanowires, hollow structures) can be accurately synthesized by regulating pH, temperature, surfactants and reaction time. Morphology further governs the specific surface area, photothermal efficiency, cellular uptake and *in vivo* circulation. Single-phase materials like metallic bismuth, bismuth oxides and Bi-based perovskites present large acoustic impedance mismatch with biological tissues and strong ultrasonic scattering. They act as US contrast agents and produce heat or ROS upon ultrasonic stimulation for sonodynamic therapy (SDT). Single-phase Bi-based materials such as perovskites and bismuth tungstate^25^, ^78^-^80^ possess layered or octahedral structures, featuring fast ion conduction and sensitive TME-response. They also maintain stable structures in the weakly acidic, ROS-rich TME to ensure durable theranostic performance.

#### 2.1.1 Bi-Based Oxygen-Containing Compounds

Bi-based oxygen-containing compounds represent the most essential and widely applied category among single-phase bismuth nanomaterials. They can be synthesized via mild routes including hydrothermal, solvothermal and precipitation methods, and predominantly present single crystalline phases such as ilmenite, layered Aurivillius and perovskite-like structures. These materials are mainly featured with layered octahedral and sheet architectures, possessing abundant intrinsic oxygen vacancies, stable lattice structures and superior ionic conductivity 81-87. Owing to these merits, they exhibit sensitive responsiveness to TME and are widely utilized for piezoelectric, photodynamic and chemodynamic sensitization. Bi-based oxygen-containing compounds gradually degrade in the weakly acidic TME with slow release of bismuth ions, resulting in low risk of heavy metal accumulation, tunable biocompatibility and favorable biosafety during *in vivo* metabolism. Wang *et al*. 73 fabricated PVP-modified copper bismuthate nano-piezoelectric sonosensitizers (PCBO) with spatially asymmetric structures (**Figure 4A**). Using tetragonal CuBi_2_O_4_ as the single-phase matrix, inequivalent Cu atoms constructed distorted [CuO_4_]^6^⁻ layers, which rotated and stacked along the c-axis to form a non-centrosymmetric point group 4 structure. Strong hybridization between Bi 6s and O 2p orbitals narrowed the band gap to 1.83 eV, and PVP surface modification further improved water dispersibility and biocompatibility. This single-phase Bi-based composite oxide integrates piezoelectric effect, rapid charge migration from narrow band gap and Cu^2^⁺-mediated glutathione (GSH) depletion. Under ultrasonic irradiation, the built-in electric field efficiently separates electron-hole pairs and greatly enhances superoxide anion production, thereby achieving synergistic tumor inhibition via piezoelectricity-augmented SDT and amplified oxidative stress.

Rapid tumor growth induces abnormal angiogenesis and insufficient oxygen supply, leading to severe hypoxia, which greatly restricts oxygen-dependent therapies such as radiotherapy, PDT and chemodynamic therapy. Shi *et al*. 25 employed sodium oleate as a soft template and carbon source to synthesize ultrathin biodegradable 2D Bi_2_O_2_CO_3_ nanosheets with abundant oxygen vacancies via a hydrothermal method. The obtained nanosheets present a thickness of only 2.8 nm with tetragonal Bi_2_O_2_CO_3_ as the single-phase matrix (**Figure 4C**). High-density oxygen vacancies (**Figure 4B**) were in-situ created due to the ultrathin geometry and surface undercoordinated sites, which optimized the band structure and radiocatalytic activity. This material serves as a low-dose radiosensitization platform with excellent X-ray absorption, radiocatalytic ROS generation, TME-responsive degradation and favorable biosafety. The ultrathin 2D structure maximizes atomic exposure and active site density. Oxygen vacancies facilitate carrier separation and singlet oxygen production.

During the synthesis of Bi-based radiosensitizers, clinical safety and compliance remain major challenges for their further development. Traditional bismuth nanomaterials adopt non-clinically approved precursors such as bismuth nitrate and bismuth citrate, which carry potential toxicity and metabolic risks. For the first time, Huang *et al*. 88 utilized Food and Drug Administration (FDA)-approved bismuth subsalicylate as the bismuth source to fundamentally address biosafety issues and barriers to clinical translation. Using transferrin as the biological template and functional ligand, transferrin-bismuth nanoparticles (TBNPs) were fabricated via a one-pot green synthetic route in aqueous solution at room temperature (**Figure 4D**). The as-prepared core-shell composites consist of metallic bismuth, bismuth oxide and transferrin, exhibiting uniform spherical morphology with an average size of ~10 nm, which favors *in vivo* circulation and blood-brain barrier penetration. The bismuth loading and structural stability were optimized by tuning the feed ratio of bismuth precursor to protein. In-situ coating with transferrin endows the nanoparticles with superior dispersibility, colloidal stability and tumor targeting capability. The composite crystalline phase is dominated by rhombohedral metallic bismuth with surface bismuth oxide. Owing to the nanoscale dimension and transferrin modification, TBNPs possess efficient blood-brain barrier permeability and active tumor targeting. Their highly active interfaces enable synergistic radio-immunotherapy for enhanced anti-tumor efficacy.

Miao and Li’s group have systematically investigated Bi-based topological nanotheranostic materials. Using BiF_3_ as the precursor, the team developed a mild topochemical synthetic strategy to achieve anion substitution and structural reconstruction, yielding single-phase sulfur-doped bismuth oxide nanomaterials. With Na_2_S as both sulfur source and reducing agent, precise regulation of reaction time, feed ratio and temperature enabled isomorphic substitution of F⁻ by S^2^⁻/O^2^⁻. The resulting materials perfectly inherit the crystal framework and nanomorphology of the BiF_3_ template, realizing highly controllable topological transformation. The sulfur-doped materials retain the face-centered cubic crystal system of the precursor with intact lattices and pure phases. Sulfur doping and topological modulation narrow the band gap, endowing the materials with excellent near-infrared (NIR) light absorption and efficient photogenerated carrier separation. Under 730 nm irradiation, they generate ROS including ·O_2_^-^ and ^1^O_2_, and deplete GSH efficiently via valence band hole oxidation and Bi^3^⁺ coordination. Meanwhile, the materials exhibit stable photothermal conversion capability and TME-responsive degradability, which enables rapid degradation in acidic, GSH/H_2_O_2_-overexpressed TME and subsequent excretion through hepatic and renal metabolism.

Du *et al*. 89 constructed a universal high-efficiency phototherapy platform based on quasi-topological bandgap engineering and morphology inheritance characteristics (**Figure 5A**). They proposed a quasi-topological bandgap modulation strategy, elucidated the dual inheritance rules of crystal morphology and phase at the structural and energy band levels, and established a facile room-temperature synthetic route with wide applicability for photothermal-photocatalytic synergistic tumor therapy. Benefiting from the flower-like structure with high specific surface area and strong NIR absorption, the platform achieves efficient anti-tumor efficacy via combined photothermal ablation and photocatalytic oxidative stress.

Chen *et al*. 90 developed topologically modulated photocatalytic nanocatalysts for NIR-responsive ferroptotic tumor therapy. Precise topological regulation and sulfur doping optimize the band gap structure and carrier separation efficiency, constructing a single NIR-activated bismuth nanocatalytic platform (**Figure 5B**). This strategy overcomes the limitations of conventional ferroptosis inducers (complex components, non-specific activation and poor versatility), which depletes GSH via photogenerated electron-hole pairs, amplifies tumor oxidative stress and triggers targeted tumor ferroptosis. Furthermore, Chen designed topological mesoporous structures to fabricate US/NIR dual-responsive drug delivery systems for immunotherapy 91. Integrating bandgap narrowing and drug loading capacity, the mesoporous topological nanocarriers break the depth limitation of conventional phototherapy and enable deep tumor treatment (**Figure 5C**). Loaded with ivermectin, the platform integrates sonocatalysis, PTT, chemotherapy and immune activation, achieving precise and controllable tumor ICD.

In addition, bismuth oxide and oxygen-nonstoichiometric bismuth oxide nanoparticles exhibit differential cytotoxicity: they exert potent killing effects solely on malignant cells while maintaining favorable biocompatibility with normal cells. The core underlying mechanism lies in that these materials only trigger controllable oxidative stress inside tumour cells with hyperactive metabolism.

Nanoscale *α*-Bi_2_O_3_ and Bi(OH)_3_ possess intrinsic structural advantages distinct from other bismuth-based nanosystems. The as-synthesized pristine Bi(OH)_3_ nanoparticles exhibit an average particle size of only 6 nm, while *α*-Bi_2_O_3_ obtained via annealing at 525 °C displays a particle size of 10 nm. Such ultrafine crystallites deliver a large specific surface area. Meanwhile, Bi(OH)_3_ contains markedly abundant surface hydroxyl groups. XPS characterization reveals that the peak area fraction of hydroxyl species in the O 1s spectrum reaches 52% for Bi(OH)_3_, higher than the 41% recorded for *α*-Bi_2_O_3_; these plentiful surface hydroxyl groups improve aqueous dispersibility and interfacial catalytic activity with cells 92. Annealed *α*-Bi_2_O_3_ adopts a pure monoclinic crystal phase with well-ordered crystal planes that supply high-density Bi^3^⁺ active sites, and the material features tunable oxygen vacancy defects, which establish a crystalline foundation for controllable ROS catalysis 93. Lamellar *α*-Bi_2_O_3_ nanostructures can be fabricated via precipitation, and this morphology boasts an elevated surface-area-to-mass ratio. Monte Carlo simulations verify that lamellar architectures outperform spherical and cubic counterparts in secondary electron yield per unit mass under X-ray irradiation, amplifying radiation energy deposition 94.

The synergistic effect of the aforementioned structural features endows the materials with unique theranostic performances: ultrafine dimensions and abundant hydroxyl groups facilitate preferential internalization by tumour cells, while tunable oxygen vacancies and Bi^3^⁺ sites initiate controlled oxidative stress exclusively within highly metabolic tumour cells, achieving differential cytotoxicity that is potent against malignant cells yet biocompatible toward normal cells. Combined with the high Z of bismuth, the lamellar crystalline structure enables efficient radiosensitization, integrating selective tumour suppression, CT contrast imaging and radiation sensitization into an all-in-one theranostic platform 95.

#### 2.1.2 Metallic Bismuth

Metallic bismuth is a distinctive category among single-phase Bi-based nanomaterials. Its monocomponent structure endows it with unique merits. Free of impurity phases and component segregation, single-phase metallic bismuth possesses uniform and tunable physicochemical properties, which mitigates performance fluctuation commonly seen in multicomponent materials. It facilitates mechanism exploration, preparation quality control and subsequent translational applications, serving as an ideal matrix for constructing standardized functional nanoplatforms. With a dense metallic lattice, metallic bismuth exhibits excellent stability under physiological conditions, which lays a solid foundation for targeted delivery *in vivo*. Additionally, it can dissociate controllably in TME, satisfying the requirements for controlled release of active substances and safe metabolism in organisms. Rich surface coordination sites enable one-step functional modification without complicated linking procedures, readily endowing the material with active targeting, immune evasion, long blood circulation and tumor-specific recognition capacities. Furthermore, single-phase metallic bismuth releases no toxic ions or inflammatory components. After surface modification, it alleviates macrophage phagocytosis, strengthens immune evasion and enhances tumor accumulation. It undergoes controllable oxidative degradation in acidic, high-GSH and hypoxic microenvironments to produce soluble bismuth ions, which can be rapidly eliminated *in vivo* without long-term accumulation. This effectively addresses the major drawback of poor *in vivo* metabolism of conventional inorganic nanomaterials.

To overcome the inherent defects of conventional high-Z nanomaterials including prominent toxicity, insufficient stability and single function, Irina Zavestovskaya *et al*. 71 developed functional bismuth nanoparticles with metallic bismuth as core and Pluronic F127 as surface modifier. Taking advantage of metallic bismuth’s high atomic number, low ionization energy and superior proton stopping power among non-radioactive elements, spherical metallic bismuth nanocrystals with uniform particle size were synthesized via a purely physical and green approach: femtosecond laser liquid ablation. Using high-purity bismuth as target in acetone medium, femtosecond pulsed laser induced melting, vaporization and explosive nucleation of the target, followed by rapid cooling. (**Figure 6A**) Particle size was precisely regulated by centrifugal classification, and polymer coating was realized via hydrophobic self-assembly. No chemical reducing agents, toxic solvents or surfactants remained throughout the process. The resultant functional bismuth nanoparticles featured narrow size distribution, outstanding colloidal stability and favorable biosafety. Under proton irradiation, the high-Z bismuth core efficiently captured proton energy within the Bragg peak region, triggering emission of low-energy Auger electrons and secondary electrons. These electrons induced water radiolysis to generate abundant ROS. Such effects enhanced local radiation dose physically and provoked oxidative stress to cause severe DNA damage chemically. Ultimately, the Caspase 3-mediated apoptosis pathway was activated, raising tumor cell apoptosis rate and colony inhibition efficiency, which effectively suppressed primary sarcoma growth and reduced lymph node metastasis potential *in vivo*.

Aiming to resolve practical challenges including low tumor accumulation, monotonous phototherapy modes and compromised therapeutic efficacy caused by tumor hypoxia, Sudip Mondal *et al*. 96 fabricated anti-epidermal growth factor receptor (EGFR) antibody-conjugated manganese phthalocyanine-bismuth nanocomposites (anti-EGFR-MPB) using metallic bismuth as the core functional substrate. Manganese phthalocyanine and Bi-based composite matrix were prepared via a mild solvothermal method. Particle surface pretreatment was conducted through PEG activation and esterification, followed by amidation for covalent conjugation of anti-EGFR antibodies (**Figure 6B**). The controllable liquid-phase reaction system enabled precise regulation of crystal phase, particle size and surface physicochemical properties, while well preserving the metallic state and high crystallinity of bismuth and achieving efficient integration of diverse functional components. Metallic bismuth (Bi^0^) acted as the core unit for NIR absorption and photothermal conversion. Manganese phthalocyanine provided photosensitivity and fluorescence signals, while DSPE-PEG improved dispersion stability in physiological media. Surface-conjugated antibodies realized specific cellular targeting. This integrated nanoplatform combined active targeting, fluorescence/PA dual-modal imaging, PTT and PDT.

On the basis of previous studies on metallic Bi-based nanotheranostics, researchers upgraded the systems via structural refinement, biomimetic interface design and functional synergy. These advancements broke through bottlenecks including inadequate targeting, unsatisfactory physiological stability, single therapeutic modality and rapid *in vivo* clearance. Compared with the above bismuth nanoparticles and antibody-conjugated manganese phthalocyanine-bismuth composites, dextran/red blood cell membrane hybrid biomimetic bismuth nanoflowers (DRBCM BNF) reported by Wen *et al*. 97 achieved remarkable progress in structural design. First, the conventional smooth spherical morphology was transformed into flower-like hierarchical structure with large specific surface area (**Figure 6C**), which greatly enhanced NIR-II light absorption and photothermal conversion efficiency (33.52%). Synchronous oxygen production and ROS generation were realized in hypoxic TME, tackling hypoxia-induced therapeutic resistance from structural perspective. Second, single polymer coating was upgraded to a dual biomimetic shell consisting of red blood cell membrane and Dextran 40. The CD47 "don’t eat me" signal on cell membranes facilitated effective macrophage evasion, and dextran offered strong steric stabilization. These improvements greatly enhanced physiological dispersion stability, prolonged blood circulation and elevated tumor accumulation. Combined PTT and radiotherapy downregulated HSP70 and inhibited p-p65 pathway, synergistically relieving tumor thermoresistance and radioresistance to achieve potent anti-tumor effects. This work provided novel physiologically compatible strategies for precision design and clinical translation of metallic Bi-based nanotheranostics.

Although morphology regulation and biomimetic coating improved the stability and combined chemo-radiotherapeutic performance of metallic bismuth materials, issues such as lack of immunomodulatory functions still existed. To address these problems, Xiao *et al*. 98 innovatively combined biological carriers with porous bismuth nanostructures, realizing key breakthroughs in structure and function. Different from DRBCM BNF that relied on passive targeting and immune evasion for tumor accumulation, the novel bifidobacteria-mediated drug-loaded porous bismuth nano-hybrids adopted porous metallic bismuth skeleton instead of dense structure (**Figure 6D**), which significantly boosted loading capacity and acid/light-responsive sustained release of hydrophobic immune agonists. Beyond traditional cell membrane biomimicry, the anaerobic bacterium-bismuth nanoparticle hybrid utilized the hypoxia tropism of bifidobacteria to achieve active targeting and colonization in deep tumors, drastically improving targeting efficiency. Functionally, the application scope was expanded from localized radio/phototherapy sensitization to PTT combined with TLR7/8 pathway-mediated anti-tumor immunotherapy, while retaining CT imaging capability. An all-in-one theranostic platform integrating deep tumor targeting, controlled drug release, PTT and immune activation was constructed. Metallic Bi-based materials were thus upgraded from adjuvants for local treatment to targeted immunotheranostic systems for hypoxic tumors, with distinctly enhanced deep tissue penetration, immune activation and abscopal anti-tumor efficacy.

#### 2.1.3 Bi-Based Multimetallic Composites

Bi-based multimetallic composites nanomaterials exhibit superior theranostic performance compared with single-component bismuth materials via rational assembly and structural design of multiple metal components. They are generally synthesized through mild phase regulation, acid-etching phase transformation or biomimetic mineralization. Atomic dispersion and interfacial coupling between bismuth and functional metals (cobalt, iron, manganese, etc.) are achieved in such composites. These materials retain bismuth’s merits, meanwhile, transition metal components endow the composites with enzyme-mimetic oxygen production, efficient electron-hole separation and STING pathway activation, fundamentally overcoming the drawbacks of conventional bismuth materials such as low ROS yield, limited efficacy against hypoxic tumors, single theranostic modality and poor tumor penetration.^76^ Carrier structures like layered double hydroxides (LDHs) and protein-based biomimetic mineralization further improve physiological stability, biocompatibility and tumor-targeted delivery. The integrated system realizes synergistic effects including enhanced sonodynamic/radiation therapy under imaging guidance, TME remodeling and anti-tumor immune activation, offering promising design strategies for multifunctional, high-efficiency and biosafe Bi-based theranostic platforms for clinical translation. Cao *et al*. 99 fabricated amorphous bismuth-doped cobalt-iron layered double hydroxides (a-CoBiFe-LDH) via phase transformation combined with acid etching (**Figure 7A**). Bandgap narrowing and oxygen vacancy introduction greatly accelerate electron-hole separation. The as-prepared sample achieves much higher US-triggered ROS generation than conventional sonosensitizers, and possesses Fe-based MRI and Bi-based CT dual-modal imaging capacities for MRI-guided high-efficiency SDT. This work breaks the functional limitations of single bismuth materials and shows great potential for clinical translation in synergistic SDT and imaging.

#### 2.1.4 Bi-based Perovskites

Bi-based perovskites possess superior ultrasonic responsiveness for sonodynamic research, benefiting from perovskite/quasi-perovskite crystal structures, intrinsic bismuth properties, and structural modulation via oxygen vacancies, doping and polarization. Typical representatives including KBiO_3_, BiFeO_3_, Bi_2_WO_6_, Bi_4_Ti_3_O_12_ feature asymmetric layered frameworks with facile lattice distortion, exhibiting prominent piezoelectric and ferroelectric behaviors. Oxygen vacancies (V_O_) can introduce discrete defect energy levels within the band gap of Bi-based semiconductors to narrow the band gap and broaden the absorption range of NIR light and US. Oxygen vacancies trap photogenerated electrons, suppress electron-hole recombination and prolong carrier lifetime. Meanwhile, the unsaturated coordination sites induced by oxygen vacancies exhibit prominent catalytic activity, lowering the energy barrier for the generation of ·O_2_^-^, ·OH and singlet oxygen, and markedly boosting the production of reactive species via sonodynamic, photodynamic and piezoelectric catalysis. In addition, oxygen vacancy-rich lattices enable reversible Bi^3+^/Bi^4+^ redox cycling, deplete intracellular glutathione and disrupt the antioxidant defense system of tumors. Under ultrasonic excitation, these materials efficiently convert mechanical energy into electrical energy to form built-in electric fields, which separate electron-hole pairs, suppress carrier recombination and improve charge utilization 100. The delocalized 6s orbitals of bismuth facilitate carrier migration. Combined with oxygen vacancy engineering, Fe-based metal doping and corona polarization, the band gap is precisely narrowed (down to 1.9 eV for KBiO_3_). Defect levels and catalytic active sites are introduced to reduce ultrasonic activation energy. Thus, ROS such as ⋅O_2_^-^ and ⋅OH are abundantly generated under low-intensity US. Cascaded amplification of oxidative stress is further realized via coupling with Fenton reaction, gas regulation and glycosylation modification. Moreover, these materials show favorable biocompatibility, biosafety and stability, as well as dual-modal PA imaging and piezoelectric imaging capabilities. With deep tissue penetration and noninvasive controllability of US, they overcome the limitations of conventional functional materials, serving as high-performance ultrasonic-responsive substrates.

Ma *et al*. 87 synthesized bismuth titanate nanosheets via one-step hydrothermal method. Surface oxygen vacancies were introduced through low-temperature hydrothermal treatment, followed by high-pressure corona polarization to obtain BTO-OVP (**Figure 7B**). The material presents a typical Aurivillius layered structure (**Figure 7C-E**), constructed by alternating stacking of (Bi_2_O_2_)^2+^ and (Bi_2_Ti_3_O_10_)^2-^. The ultrathin 2D structure shortens carrier migration pathways. Oxygen vacancies act as electron traps to narrow band gaps and inhibit electron-hole recombination. Corona polarization aligns ferroelectric domains and strengthens built-in electric fields, enhancing piezoelectric response and charge separation. Synchronous ROS generation and catalytic CO_2_ reduction to CO are achieved under US, enabling efficient synergistic sonopiezoelectric therapy.

Ding *et al*. 101 fabricated Fe-doped bismuth tungstate nanosheets with oxygen defects via one-pot hydrothermal synthesis, and modified the surface with PEG-NH_2_ to improve dispersibility (**Figure 7F**). The products retain the orthorhombic 2D layered structure of Bi_2_WO_6_ (**Figure 7G**). Uniform Fe lattice doping induces abundant oxygen vacancies, which narrow the band gap, boost ultrasonic activation and endow the material with Fenton-like activity. Meanwhile, piezoelectric effect drives directional carrier migration and alleviates recombination. The integrated piezoelectric sonodynamic and chemodynamic therapy greatly elevates ROS yield and anti-tumor efficacy.

### 2.2 Bi-based Typical Heterojunction Materials

Compared with monophasic Bi-based nanomaterials, Bi-based heterojunction nanomaterials break the inherent limitations of single-phase systems in band structure, carrier separation and conversion efficiency of light, acoustic and ray energy via interfacial coupling between diverse Bi-based semiconductors, or between Bi-based materials and non-bismuth semiconductors/metals, emerging as a core upgraded system of Bi-based theranostic nanomaterials. Relying on band engineering, built-in electric field construction and multi-component functional synergy at heterojunction interfaces, these materials present distinct classifications, unique architectures and innovative mechanisms for precise tumor theranostics. Typical configurations include p-n Bi-based semiconductor heterojunctions 102 (e.g., coupled Bi-based semiconductors with different conductivity types such as BiF_3_/Bi_2_O_3-x_:S_x_), bismuth/non-bismuth semiconductor heterojunctions 103 (e.g., Bi-based materials combined with conventional semiconductors like MnO_x_), bismuth-metal Schottky heterojunctions 104 (e.g., Schottky interfaces formed between bismuth and noble metals including Pt and Au), as well as self-heterojunctions/interlayer heterojunctions 105 (e.g., intra-crystalline self-heterostructures of oxygen-deficient bismuth oxysalts). Each category achieves performance improvement through specific charge transfer pathways.

Well-matched bismuth-based heterojunctions trigger interfacial band bending via Fermi level equilibration and build an internal electric field to drive directional charge migration. Z-scheme and S-scheme heterojunctions recombine low-energy charge carriers and spatially separate high-energy redox carriers, retaining strong redox capacity to facilitate cascade catalysis. Bismuth metal-based Schottky junctions confine free electrons via localized surface plasmon resonance (LSPR) to simultaneously boost photothermal conversion and catalytic performance. Atomically tight heterointerfaces reduce charge transport resistance and suppress interfacial recombination. Interfacial lattice matching, defect coupling and elemental interdiffusion modulate band offset, improve the separation efficiency of photogenerated carriers, and balance catalytic activity and biocompatibility.

Structurally, Bi-based heterojunction nanomaterials feature atomically tight interfaces, multidimensional hierarchical morphologies, tunable defects, and intrinsic properties of high-Z bismuth. They enable efficient carrier separation and transport, optimize specific surface area, dispersibility and biocompatibility, and exhibit outstanding performance for tumor radiosensitization as well as sonodynamic/piezoelectric therapy.

During radiotherapy, the strong X-ray attenuation of bismuth enhances radiation energy deposition. Heterojunction interfaces facilitate the dissociation of electron–hole pairs, boost the production of ROS (·OH) and induce DNA double-strand breaks, thereby achieving effective radiosensitization at low doses. In sonodynamic piezoelectric therapy, built-in electric fields cooperate with piezoelectric effects to suppress carrier recombination. Certain systems relieve tumor hypoxia via photothermal assistance and in-situ catalytic oxygen generation, triggering multiple cell death pathways including apoptosis, pyroptosis, ferroptosis and PANoptosis, activating anti-tumor immunity and remodeling the TME, providing theoretical and experimental foundations for constructing high-performance Bi-based heterojunction theranostic nanoplatforms.

Yang *et al*. 106 developed a 1D bamboo-segmented core-shell heterojunction sonosensitizer (MnO_2_@Mn-BiVO_4_, BMMBV). With BiVO_4_ nanorods as the backbone, surface amination was performed prior to redox reaction and selective etching with KMnO_4_ (**Figure 8A**). Amorphous MnO_2_ shells grew *in situ*, while Mn^2^⁺ was doped into the BiVO_4_ lattice, forming an axially segmented bamboo-like p-n heterojunction. Subsequent DSPE-PEG-PEI modification guaranteed its stable dispersion. This design synergizes three merits: Mn doping-induced band gap narrowing for enhanced piezoelectric sonodynamic effect, Mn^2^⁺ release from responsive degraded MnO_2_ to activate the STING pathway, and efficient cellular uptake and tumor penetration of 1D nanostructures. Under US, the material produces abundant ROS, induces ICD, and reverses MHC-I deficiency-related tumor immune escape.

Lv *et al*. 107 adopted a co-precipitation, in-situ reduction and surface coating strategy to fabricate the target material (Figure 8B). 2D Ce-doped BiOCl nanosheets were first synthesized via co-precipitation, followed by in-situ loading of Pt nanoclusters, and final encapsulation with polyglutamic acid (PGA). The as-prepared sample presents uniform 2D sheet morphology. Ce substitution at Bi sites creates abundant oxygen vacancies in BiOCl lattice, which constructs Schottky heterojunctions with Pt (Figure 8C). The outer PGA layer improves dispersibility and biocompatibility. The Ce^4^⁺/Ce^3^⁺ redox cycle boosts Fenton-like reactions, and Pt accelerates charge transfer as well as endows dual-enzyme catalytic activity. The sheet structure enlarges the specific surface area. Upon US irradiation, dual-pathway in-situ oxygen generation relieves tumor hypoxia, accompanied by massive ROS production and GSH depletion, achieving high-efficiency synergistic sonodynamic-immunotherapy.

Xiao 108 fabricated a piezoelectric-enhanced NIR photocatalytic Bi/SrTiO_3_ bimetallic Bi-based nanoheterojunction (Figure 9A). Metallic Bi with localized LSPR was in-situ anchored on piezoelectric SrTiO_3_ nanocubes to form intimate metal-semiconductor heterostructures (Figure 9B-E). After amination, the composite was incorporated into injectable, self-healing and pH-responsive gelatin/oxidized chondroitin sulfate hydrogel, yielding BGO composite hydrogel with dual functions of anti-tumor and osteogenesis. Centered on Bi/SrTiO_3_ heterojunctions, this system establishes Schottky barriers and built-in electric fields via interface engineering. The retained piezoelectric phase of SrTiO_3_ enables piezoelectric-driven charge separation. Uniformly dispersed ~ 3 nm ultrafine Bi nanoparticles introduce LSPR effect to broaden NIR absorption. Silanization realizes covalent crosslinking with hydrogel, and pH-responsive hydrogel achieves targeted drug release in TME. Under combined US and NIR irradiation, the synergistic effect of heterojunctions remarkably elevates ROS production to kill osteosarcoma cells. Under mild US, piezoelectric electrical stimulation activates the PI3K/AKT pathway to accelerate bone regeneration.

Controllable conversion of multivalent metal ions (Ce^3^⁺/Ce^4^⁺, Fe^2^⁺/Fe^3^⁺) enables the construction of endogenous redox catalytic cycles. The variable-valence sites act as electron transport bridges to accelerate charge transfer and trigger Fenton/Fenton-type cascade reactions, continuously generating reactive oxygen species under the stimulation of the tumor microenvironment. Valence regulation can also tune band edge positions to match the redox potentials of intracellular substrates including glutathione, hydrogen peroxide and oxygen, achieving tumor-specific stimulus-responsive catalytic activation. To address the clinical limitations of compromised sonodynamic efficacy and insufficient anti-tumor immunity caused by tumor hypoxia, Pan 109 developed an US-TME dual-responsive multi-component Bi-based heterojunction nanocatalyst (Figure 9F). Using 2D BiOCl as the matrix, Ce ion doping induces lattice defects and Ce^3^⁺/Ce^4^⁺ redox cycles. Pt nanoclusters were further grown in-situ to construct BiOCl:Ce-Pt heterojunctions, followed by PGA functionalization to obtain multifunctional theranostic agent BiOCl:Ce-Pt@PGA (BCCP) (Figure 9G-H). Multi-dimensional structural optimization was achieved via crystal defect regulation, heterojunction interface engineering, 2D morphology control and surface modification. The BiOCl:Ce-Pt heterojunction narrows band gap and facilitates carrier separation and interfacial electron transport, producing abundant ROS for potent SDT under US. Ce doping endows the system with Fenton-like activity and high oxygen evolution capacity. Pt nanoclusters with dual-enzyme activity realize dual-pathway in-situ oxygen generation to reverse tumor hypoxia. The 2D structure and oxygen vacancies effectively deplete GSH and trigger ferroptosis. PGA coating improves *in vivo* delivery and biocompatibility. This design integrates Bi-based semiconductor sonocatalysis, Ce-mediated Fenton-like reaction, Pt nanozyme catalysis, GSH depletion and ICD activation, achieving US-augmented self-oxygenated sonodynamic-immunotherapy.

### 2.3 Non-heterojunction Core-Shell Coated Composites

Core-shell structure brings breakthroughs for performance optimization, functional integration and clinical translation of Bi-based nanomaterials. Current core-shell Bi-based theranostic nanomaterials share identical design features: with elemental bismuth, bismuth oxides, sulfides or heterostructures as inner cores, functional shells including silica, covalent organic polymers, metal oxides, biological membranes and bacterial vesicles are epitaxially grown. Such structurally stable and interface-tunable core-shell heterostructures are widely applied in multimodal imaging and synergistic combination therapy of tumors, showing prominent advantages in targeted theranostics and anti-metastasis research of solid tumors such as non-small cell lung cancer, breast cancer and liver cancer. The core-shell interface modulates electronic structure and accelerates carrier separation, thereby enhancing photothermal, photodynamic and catalytic effects. Serving as a physical barrier, the shell prevents core aggregation and premature degradation to improve physiological stability. Via pore regulation and surface modification, it efficiently loads chemotherapeutics, hypoxia-activated prodrugs and gene fragments for stimuli-responsive release. Modular core-shell design separately optimizes the theranostic performance of cores and biological functions of shells including targeting, long circulation and immune evasion, overcoming the limitations of single-component materials. Shells modified with antibodies or hybrid biomembranes endow nanomaterials with active tumor targeting, enhancing tumor accumulation and reducing systemic toxicity. Degradable shells composed of silica, metal oxides and covalent organic polymers respond to the acidic microenvironment, high GSH and hydrogen peroxide in tumors, achieving targeted disassembly and functional activation with guaranteed precision and biosafety. Furthermore, this structure enables facile integration of multimodal imaging and combined therapy, constructing an all-in-one "diagnosis-therapy-monitoring" platform and offering new strategies for precise and individualized tumor treatment.

In the field of microenvironment-responsive theranostics, BDS GO_x_@MnO_x_ core-shell nanozymes (Figure 10A) 110 consist of bismuth nanocrystal cores, stimuli-responsive MnO_x_ shells and loaded glucose oxidase. The composites are fabricated via in-situ redox reaction and interfacial self-assembly (Figure 10B). This modular core-shell structure enables dual-modal CT/MRI imaging and triple activation responses to pH, GSH and glucose. It overcomes the drawbacks of conventional nanozymes including single response mode, isolated therapeutic modalities and disconnection between imaging and treatment. Cascade-enhanced starvation therapy and chemodynamic therapy are achieved, offering an efficient strategy for theranostics of triple-negative breast cancer.

To address the challenges in brain tumor radiotherapy, Fe_3_O_4_@Bi_2_S_3_ core-shell nanoparticles (Figure 10D) 68 are synthesized via solvothermal and template transformation methods. Composed of magnetic Fe_3_O_4_ cores, high-Z Bi_2_S_3_ shells and homologous cell membrane coatings, the system integrates magnetic targeting, radiosensitization and biological targeting (Figure 10C). It efficiently crosses the blood-brain barrier and accumulates in glioma tissues. Combined radiotherapy and ferroptosis pathways are realized under real-time MRI guidance for precise radiosensitization, which remarkably improves the therapeutic efficacy of orthotopic glioblastoma and reduces damage to normal tissues.

To tackle major obstacles in clinical treatment of non-small cell lung cancer, such as immunosuppressive microenvironment, silenced cell death pathways, poor drug targeting and drug resistance of monotherapy, Xu 111 developed a quadruple-layer biomimetic core-shell nanoreactor named BSCAPM (Bi@SiO_2_@COP@AQ4N&PTX@hybrid membrane). Metallic bismuth nanoparticles are first prepared by chemical reduction, followed by SiO_2_ coating via the Stöber reaction. A COP shell is then grown through solvothermal Schiff-base reaction to co-load paclitaxel (PTX) and hypoxia-activated prodrug AQ4N. Finally, hybrid membranes fused by erythrocyte and lung cancer cell membranes are coated via membrane extrusion to construct a functionally modular core-shell theranostic system (**Figure 11 A-B**). This structure features distinct functional zones, efficient interfacial synergy and favorable biocompatibility. The Bi core provides 808 nm laser-driven photothermal conversion and CT imaging capability. The interlayer SiO_2_ modulates electron transport, facilitates carrier separation and induces mitochondrial dysfunction. The porous COP shell generates ROS for PDT and achieves high drug loading capacity. The outer hybrid membrane endows nanoparticles with long circulation, immune evasion and homologous active targeting towards lung cancer. Nevertheless, such highly complex multicomponent nanosystems face potential obstacles in large-scale production and batch-to-batch reproducibility.

For minimally invasive treatment of superficial tumors, Bi/Bi_2_SiO_5_@SiO_2-x_ core-shell nanocomposites 112 are combined with dissolvable microneedle platforms. A gradient functional core-shell structure is synthesized through hydrothermal treatment and high-temperature reduction. The SiO_2-x_ shell stabilizes Bi cores and boosts ROS production, and realizes pH/light/US dual-gas (NO and H₂) responsive release. This design solves the limitations of poor targeting, systemic toxicity of gas therapy and insufficient penetration depth of PTT/SDT. It achieves CT-guided minimally invasive percutaneous combined treatment, establishing a non-invasive and well-tolerated theranostic paradigm for superficial tumors such as breast cancer.

In summary, rationally designed Bi-based core-shell nanomaterials integrate diverse imaging modes, stimuli responses, therapeutic mechanisms and delivery strategies. They exhibit prominent synergistic effects and precise controllability in tumor theranostics, providing systematic guidelines and theoretical support for the construction of high-performance multifunctional nanotheranostic platforms.

### 2.4 Bi-based/containing Metal-Organic Framework Nano-Materials

Bi-based/containing MOFs are constructed via coordination self-assembly between Bi^3^⁺ metal nodes and organic ligands to form multidimensional porous stable frameworks, whose functions can be modularized through doping, modification, coating and drug loading. Featuring favorable biocompatibility and degradability, they exhibit TME responsiveness and enable efficient loading and sequential release of functional molecules. Their structure and pore channels are precisely tunable; specific morphologies accelerate carrier migration and boost ROS generation via photo/sonocatalysis. Surface functionalization also endows the materials with targeting ability, stimuli responsiveness and interfacial synergism. Bismuth nodes absorb X-rays to realize radiosensitization and deplete GSH to disrupt tumor redox homeostasis for enhanced therapeutic efficacy. Organic ligands act as photosensitizers and sonosensitizers to further increase ROS yield. These MOFs degrade specifically in TME, avoiding *in vivo* accumulation. Via modular design, they integrate imaging, multimodal therapy and immune activation, showing great potential for high-efficiency, low-toxicity and precise synergistic theranostics of solid tumors including breast cancer, cervical cancer, colorectal cancer and lung cancer.

In the field of cascade catalytic synergistic therapy, Zhang 113 reported an ultrasmall bismuth-copper bimetallic coordination polymer as a nanotheranostic platform (Figure 12A). Synthesized via Bi-O and Cu-O chelation between Bi^3^⁺, Cu^2^⁺ and gallic acid ligands, the material possesses a stable organic-inorganic coordination network. Its ultrasmall particle size and flexible skeleton endow it with renal clearance, high photothermal conversion efficiency and Fenton-like catalytic activity. After phospholipid coating and glucose oxidase immobilization, an integrated nanoplatform for starvation therapy, chemodynamic therapy and mild-temperature PTT was constructed. This system overcomes the drawbacks of conventional treatments, including insufficient endogenous H_2_O_2_ for chemodynamic therapy, heat shock protein-induced drug resistance in PTT, and long-term toxicity caused by accumulation of inorganic nanoparticles, achieving safe and efficient multimodal synergistic tumor inhibition. To boost the efficacy of combined radiochemotherapy, Chang 70 developed a biomimetic IZB@CCM MOF delivery system. This ZIF-8-based bismuth-loaded nanosystem has a rigid framework assembled from Zn^2^⁺ and 2-methylimidazole via Zn-N coordination, where bismuth nanodots and FAK inhibitor IN10018 are co-encapsulated (Figure 12B-C). Coated with hybrid membranes of cancer cells and cancer-associated fibroblasts (CAFs), it serves as a dual-targeted delivery vehicle. The porous framework enables high drug loading and acid-responsive drug release, while the outer hybrid membrane confers homologous targeting and immune evasion. The bismuth component enhances radiation energy deposition to realize effective radiosensitization. It fundamentally addresses clinical challenges in cervical cancer radiotherapy, such as CAF-mediated radioresistance, short circulation half-life, poor targeting and off-target toxicity of small-molecule inhibitors, thereby improving therapeutic outcomes and alleviating adverse effects.

This chapter systematically reviews the design strategies and research advances of four categories of Bi-based tumor theranostic nanomaterials, elemental/single-phase Bi-based nanomaterials, non-heterojunction core-shell coated composites, typical Bi-based heterojunction nanomaterials, and Bi-based/containing metal-organic framework nanomaterials, with a horizontal comparative analysis conducted from the perspectives of crystal architecture, interfacial characteristics, core synergistic mechanisms, and application merits. Single-phase bismuth materials serve as the fundamental substrate of the whole material system. Their band structures can be precisely tuned via morphological modulation, defect engineering and elemental doping, while they possess outstanding X-ray attenuation capacity and TME-responsive degradability, which renders them universal substrates for photodynamic, sonodynamic and radiotherapeutic sensitization. Bi-based heterojunctions construct atomically coupled interfaces through in-situ lattice matching, where spontaneous built-in electric fields are generated to hinder charge carrier recombination, intrinsically boosting ROS production and breaking the catalytic performance bottleneck of single-phase counterparts. Non-heterojunction core-shell composites adopt a post-coating modular architecture: the core bears intrinsic theranostic properties, whereas the outer shell endows nanoplatforms with biological functions including prolonged blood circulation, homologous tumor targeting and stimuli-responsive drug release, thus realizing integrated multi-modal functionalities. Benefiting from well-ordered porous coordination skeletons, Bi-MOFs deliver ultrahigh drug loading efficiency and customizable pore engineering, coupled with favorable biodegradability, making them ideal candidates for co-delivery of multiple agents and cascade catalytic therapy. Distinct structural regulation routes adopted by the four types of bismuth nanomaterials address distinct clinical hurdles including insufficient efficacy of monotherapy, tumor hypoxia, immunosuppression and off-target toxicity, which offers hierarchical and systematic structural design guidelines for the fabrication of high-performance, clinically translatable multifunctional tumor theranostic nanoplatforms.

## 3. Bismuth-Based Nanomaterials for Tumor Imaging and Visualized Therapy

Bi-based nanomaterials possess unique physicochemical properties and hold great potential for tumor theranostics, especially in multimodal imaging. Current BiNMs have been extensively studied for CT imaging, MRI, PAI, US and fluorescence imaging.

### 3.1 Computed Tomography Imaging

Metallic bismuth features low cost and superior physicochemical performance. With an atomic number of 83 (Au: 79, Pt: 78, Ta: 72), its X-ray attenuation coefficient reaches 5.74 cm^2^·g⁻^1^ at 100 keV, outperforming heavy metals including gold (5.16 cm^2^·g⁻^1^), platinum (4.99 cm^2^·g⁻^1^) and tantalum (4.3 cm^2^·g⁻^1^). Its outstanding X-ray absorption capacity makes it an ideal candidate for radiosensitization and CT contrast imaging. The interaction between X-rays and matter is dominated by the photoelectric effect and Compton scattering. High-Z elements like bismuth produce intense photoelectric effect via K-shell inner electrons. The probability of this effect is proportional to the fourth power of atomic number and inversely proportional to the cube of photon energy, which greatly improves X-ray absorption efficiency. Stronger X-ray attenuation generates bright signals on CT images, effectively enhancing lesion distinguishability and imaging contrast.

Nanoscale Bi-based materials such as Bi quantum dots and Bi_2_O_3_ nanoparticles deliver larger specific surface areas. Surface modification can regulate their *in vivo* biodistribution and pharmacokinetics, further optimizing their performance as CT contrast agents. For instance, the ternary Bi-based heterojunction nanozyme Bi@Bi_2_O_3_-Pt-PEG (BBOP) 114 is engineered with Z-scheme heterojunctions, Schottky contacts and oxygen vacancies, endowing the material with sonocatalysis, catalase-like activity and immunomodulatory functions for CT imaging of orthotopic and metastatic breast cancer. Its HU value shows a strong positive linear correlation with concentration. Benefiting from high X-ray attenuation of bismuth, BBOP achieves excellent CT imaging performance. Tumor accumulation peaks at 3 h post intravenous injection, enabling accurate visualization of tumor size, boundary and distant metastases. It provides real-time positioning for sonocatalytic immunotherapy and realizes integrated imaging and therapeutic monitoring of metastatic tumors with high signal-to-noise ratio and spatiotemporal resolution.

Defective Bi-based homojunction Schottky heterostructures (Bi HJ) 115, are applied for CT imaging in mouse 4T1 subcutaneous breast cancer models. It exhibits better *in vitro* imaging performance than iodixanol. The CT signal at tumor sites peaks at 12 h post injection (about 40 HU), 2.3 times that at 1 h, allowing long-term and clear delineation of tumor margins (Figure 13A-B). Composed solely of bismuth without toxic components, it achieves high-safety precise tumor diagnosis and image-guided therapy.

Wang *et al*. 116 developed lipid-coated Bi-based nanoflower microsphere dry powder inhalants (DP BNF@Lat MPs), for CT imaging of breast cancer lung metastasis. At the same concentration, its *in vitro* CT signal intensity is 3.5 times that of clinical iohexol, with a linear relationship between HU value and concentration (R^2^=0.9955). After intratracheal administration, the CT value of lung metastatic lesions rises markedly within 30 min (Figure 13C-E). This agent clearly identifies tumor location and distribution, realizing non-invasive and high-resolution targeted imaging of lung metastases.

### 3.2 Magnetic Resonance Imaging

Bulk bismuth is non-paramagnetic and thus cannot serve as an MRI contrast agent directly. Rational structural design and functionalization enable Bi-based nanomaterials for MRI. Common strategies include incorporating paramagnetic ions (e.g., Fe^3^⁺, Gd^3^⁺, Mn^2^⁺) or utilizing localized electron spins from surface defects to modulate water proton relaxation. Gd³⁺ possesses seven unpaired 4f electrons with strong paramagnetism, which effectively shortens T₁ relaxation time and generates bright signals on T₁-weighted MRI. Gd chelate-functionalized bismuth nanomaterials have been developed as theranostic agents for multimodal MRI/CT/PAI imaging 117.

Magnetic nanoparticles such as Fe_3_O_4_ are widely investigated for MRI. Doping with Mn^2^⁺ and Zn^2^⁺ improves magnetic properties to achieve dual-modal T_1_/T_2_ MRI. For instance, glioma cell membrane-camouflaged Fe_3_O_4_@Bi_2_S_3_ nanoparticles (FBM) 68 employ superparamagnetic Fe_3_O_4_ as T_2_ MRI moiety and Bi_2_S_3_ as a radiosensitizer. *In vitro* results reveal concentration-dependent negative T_2_ enhancement, with a transverse relaxivity *r_2_* of 24.36 mM⁻^1^·s⁻^1^ and a linear correlation coefficient *R^2^* of 0.99, demonstrating favorable imaging performance (Figure 14A). In orthotopic glioma models, FBM accumulates abundantly in tumors via magnetic targeting and homologous recognition. The T_2_ signal reaches the minimum at 12 h post-injection with optimal contrast, allowing clear tumor demarcation and supporting MRI-guided precise radiotherapy. Bi/Mn bimetallic biomineralized nanoparticles (BiMn/BSA) 76 produce positive T₁ enhancement via Mn^2^⁺ paramagnetism, while bismuth enables CT imaging and radiosensitization. *In vitro* T_1_ signal increases in a concentration-dependent manner (Figure 14B). *In vivo*, low-dose radiotherapy facilitates macrophage infiltration and stroma degradation, promoting deep tumor penetration of BiMn/BSA. Its tumor MRI signal is 5.4-fold higher than that from the passive enhanced permeability and retention effect, greatly improving visualization of tumor parenchyma and margins, and enabling real-time imaging monitoring for intratumoral delivery and uniform distribution of nanomedicines.

Cao *et al*. 99 synthesized amorphous bismuth-doped CoFe layered double hydroxide (a-CoBiFe-LDH-PEG). Paramagnetic Fe^2^⁺/Fe^3^⁺ endows the material with high-performance T_2_-weighted MRI. Its *r₂* value reaches 176.9 mM⁻^1^·s⁻^1^, much higher than conventional iron-based contrast agents. The nanoparticles rapidly accumulate in tumors after intravenous injection, with maximal T_2_ contrast at 8 h post-administration. The sustained imaging window covers the entire theranostic process, and the signal returns to baseline within 24 h, indicating favorable metabolic clearance (Figure 14C). This system realizes precise SDT and therapeutic evaluation under MRI guidance. Collectively, the three types of Bi-based composites achieve high relaxivity, excellent tumor targeting and prolonged imaging windows via structural engineering, establishing quantifiable and reproducible high-performance contrast platforms for image-guided precise tumor therapy.

### 3.3 Photoacoustic Imaging

PA imaging is an emerging modality that combines the advantages of optical and ultrasonic imaging. It relies on strong NIR absorption of materials, where photothermal conversion generates acoustic signals detectable by ultrasonic transducers. Many narrow-bandgap Bi-based semiconductors, including Bi_2_S_3_, Bi_2_Se_3_ and BiOI, show intrinsic plasmon resonance or interband transition absorption in the NIR-I/II region (700-1700 nm). Nanostructures such as nanoflowers, nanosheets and hollow spheres can boost photothermal conversion efficiency and PA signal intensity. Signal reconstruction enables high-contrast and deep-penetration tissue imaging, and these materials deliver distinct PA generation mechanisms and imaging performances due to varied compositions and structures.

BiOI-loaded hollow MnO_2_-coated bacterial membrane vesicles (MB@MV) 118 exhibit synergistic NIR absorption from bismuth and MnO_2_, producing PA signals under 808 nm laser irradiation for tumor blood oxygenation monitoring. Compared with PBS and MVs groups, MB slightly elevates tumor oxyhemoglobin content (Figure 15A). Benefiting from bacterial membrane vesicle-mediated tumor targeting, MB@MV significantly enhances PA signals of oxyhemoglobin in tumors. It allows precise visualization of hypoxia relief and clear discrimination between hypoxic and normoxic tumor regions. Some Bi-based perovskites also present excellent PAI performance. Non-stoichiometric BiFeO_3-x_ nanoparticles 65 are transformed *in situ* into strongly NIR-absorptive Bi_2_S_3_ triggered by high-concentration H_2_S in TME, thus activating PA signals. *In vitro* PA signals increase linearly with NaHS concentration (R^2^ = 0.9816). After intratumoral injection, the PA signal peaks at 4 h with a 3.22-fold enhancement relative to the initial state and remains stable for over 5 h, achieving H_2_S-responsive and highly specific tumor PA imaging and therapeutic guidance (Figure 15B). Polyvinylpyrrolidone-modified copper bismuthate (PCBO) developed by Wang *et al*. 73 generates intrinsic PA signals owing to the narrow bandgap and broad NIR absorption of CuBi_2_O_4_. The maximum PA signal in tumors appears at 24 h post intravenous injection. This agent clearly delineates tumor boundaries and intratumoral distribution with much higher signal intensity than normal tissues (Figure 15C). With favorable concentration dependence and passive tumor targeting ability, it provides accurate temporal and spatial guidance for SDT.

### 3.4 Fluorescence Imaging

Fluorescence imaging relies on the property that materials emit fluorescence at different wavelengths after absorbing incident light. Bi-based nanomaterials are promising fluorescent probes. The Bi^3^⁺ ion with a 6s^2^ lone-pair electron configuration acts as a sensitizer or luminescent center 119. Doping with rare-earth ions (e.g., Eu^3^⁺, Mn^2^⁺) or constructing bismuth halide perovskites (e.g., Cs_3_Bi_2_I_9_) enables fluorescence emission spanning visible to NIR regions. Curcumin-based carbon quantum dots doped with gadolinium serve as multifunctional fluorescent imaging agents.^120^ Quantum confinement and surface passivation (e.g., silica coating) greatly improve the quantum yield and photostability of Bi-based nanomaterials 119. 2D materials such as graphene and MXene can also gain fluorescence via doping or surface modification.

Song 74 fabricated a protoporphyrin-sensitized degradable wide-bandgap Bi-based semiconductor nanotheranostic (P-NBOF). Yb-doped bismuth oxyfluoride (Na_0.2_Bi_0.8_O_0.35_F_1.91_) served as the mesoporous core. Protoporphyrin (PpIX) was immobilized via carboxyl coordination as an US sensitizer, followed by amphiphilic PEG coating to improve water dispersibility and biocompatibility. P-NBOF exhibits intrinsic fluorescence from loaded PpIX. Upon excitation at 630 nm, it emits characteristic red fluorescence at 700 nm. In cellular assays, the fluorescence intensity in 4T1 cells increased with incubation time and peaked at 24 h, indicating efficient cellular uptake. *In vivo* fluorescence imaging revealed continuously enhanced tumor signals with maximum intensity at 12 h post intravenous injection. The tumor showed high contrast and clear boundaries, with its fluorescence intensity 2.8-fold higher than normal tissues. The fluorescence distribution was highly consistent with the intratumoral bismuth accumulation detected by ICP-OES, realizing accurate tumor fluorescence imaging and biodistribution verification.

### 3.5 US Imaging

US imaging generates images based on acoustic reflection and scattering at tissue interfaces 121. Bi-based nanomaterials act as effective US contrast agents due to their distinct acoustic impedance from soft tissues. The acoustic impedance of bismuth (Z ≈ 2.6–3.0×10^6^ kg·m⁻^2^·s⁻^1^) is much higher than that of soft tissues (Z ≈ 1.6×10^6^ kg·m⁻^2^·s⁻^1^), facilitating strong acoustic reflection 122.

To further amplify US signals, Bi-based nanomaterials are engineered into micro/nanobubble composites or hollow/porous structures. Such architectures possess larger acoustic scattering cross-sections and yield high-contrast US images. For instance, encapsulating Bi_2_S_3_ nanoparticles into lipid microbubbles or loading BiOBr nanosheets onto echogenic liposomes can remarkably boost imaging performance. Meanwhile, researchers have fabricated Bi-based nanocomposites loaded with perfluorocarbon phase-change nanodroplets. Acoustically induced phase transition enables the instantaneous generation of microbubbles under an US field, which markedly amplifies the signal intensities of harmonic US imaging 123. This phase-transition strategy not only elevates imaging contrast but also permits spatiotemporal regulation of microbubble formation by tuning US frequency and power intensity 124.

In addition, hollow or porous Bi-based nanospheres (e.g., hollow Bi_2_S_3_ nanospheres) further boost the signal-to-noise ratio of US imaging by reducing bulk density and enlarging acoustic scattering cross-sections 125. Another cutting-edge design relies on piezoelectric Bi-based materials, such as BiFeO_3_ and doped bismuth oxides. Mechanical vibration triggered by ultrasonic stimulation induces piezoelectric polarization in these materials. Such polarization facilitates the production of ROS for SDT; meanwhile, interfacial charge rearrangement modulates local acoustic properties to indirectly improve imaging contrast 126.

### 3.6 Integration of Multimodal Imaging

In the development of multifunctional theranostic platforms, Bi-based nanomaterials exhibit versatile performance in multimodal imaging. Combined multimodal imaging strategies are widely adopted to enable accurate diagnosis and therapeutic evaluation. Rational nanostructure design and surface modification overcome the limitations of single-modal imaging and provide comprehensive information for precise tumor diagnosis and treatment 117, 127, 128.

CT/MRI dual-modal imaging is the most prevalent combination for Bi-based materials. Bismuth with high X-ray attenuation coefficient enables CT imaging with high spatial resolution and clear anatomical morphology, while magnetic components such as Mn and Fe endow MRI with superior soft-tissue contrast and functional metabolic information, constructing a complementary anatomical and functional diagnostic system. For BDS-GO_x_@MnO_x_ nanoflowers 110, bismuth acts as the CT imaging core and Mn serves as the T_1_-weighted MRI contrast unit. Its *in vitro* CT attenuation slope reaches 61.99 HU·mM⁻^1^, outperforming clinical iodine-based contrast agents (**Figure 16A**). Sustained Mn^2^⁺ release in acidic TME increases the longitudinal relaxivity *r₁* to 5.30 mM⁻^1^·s⁻^1^. CT precisely delineates tumor anatomical boundaries and morphology, and MRI distinguishes intratumoral necrosis and invasion areas, achieving full-dimensional visualization of tumor location, size and internal structure. CDBi,Mn-FA@CaP nanoparticles 129 also adopt bismuth-manganese composite design. Bismuth produces stable CT enhancement, and Mn imparts T_1_-weighted MRI capability. Acidic microenvironment triggers particle size transformation and simultaneous enhancement of dual-modal signals. CT compensates for the poor performance of MRI in imaging calcified and osseous tissues, while MRI remedies the low soft-tissue resolution of CT, enabling accurate evaluation of tumors from macroscopic localization to microscopic invasion (**Figure 16B**).

CT/PAI dual-modal imaging integrates deep-penetration structural imaging and high-sensitivity functional imaging, based on the strong X-ray and NIR absorption of Bi-based materials. Bismuth serves as the core signal source for CT, while PA imaging acquires acoustic signals from photothermal conversion to dynamically monitor tumor blood oxygenation, blood flow and nanoparticle accumulation (**Figure 17B**) 96. Gold-capped Bi-based nanocomposites (BACNs) 130 show a positive linear correlation between PA signal intensity and concentration under 1064 nm NIR-II irradiation, clearly outlining tumor contour and intratumoral distribution. The synergistic effect of bismuth and gold significantly elevates CT HU values with increasing material concentration. PA imaging compensates for the inability of CT to monitor tumor physiological functions in real time, and CT provides accurate anatomical references for PA imaging, realizing integrated structural and functional tumor diagnosis (**Figure 17A**). Defect-engineered Fe_3_O_4_@Au@Bi_2_S_3_ nanospheres 67 possess enhanced NIR absorption. PA imaging tracks the targeted accumulation of nanoparticles in real time. Benefiting from the high atomic numbers of bismuth and gold, CT achieves high-contrast tumor imaging under magnetic targeting for clear differentiation between tumor and normal tissues.

CT/MRI/PA dual- and trimodal imaging further integrate the advantages of structural, functional and molecular imaging. As core signal carriers, Bi-based materials integrate CT, MRI and PA functions to realize full-process diagnosis including anatomical localization, functional assessment and molecular tracing 69, 72, 111, 131. In this system, CT provides macroscopic anatomical and positional information, MRI offers high-resolution soft-tissue imaging and evaluates tumor invasion, and PA imaging dynamically reflects tumor hypoxia, blood perfusion and nanoparticle metabolism. These modalities complement each other and address the drawbacks of single-modal imaging such as limited penetration depth, insufficient resolution and lack of functional data. This strategy enables accurate identification of tumor location, size and morphology, as well as dynamic monitoring of TME and nanomedicine delivery. It provides real-time, comprehensive and precise imaging guidance for theranostics, and greatly improves the accuracy of early tumor diagnosis and therapeutic efficacy evaluation.

Although Bi-based nanoplatforms can effectively integrate the merits of CT, MRI, PA, fluorescence and US imaging to achieve synergistic visualization of tumor structure and function, current multimodal imaging systems still face several critical challenges. Distinct modalities exhibit remarkable discrepancies in spatiotemporal resolution: CT delivers ultrahigh spatial resolution yet lacks functional information; MRI features outstanding soft-tissue contrast but requires lengthy acquisition time; PA and fluorescence imaging possess high sensitivity while suffering from limited penetration depth. These limitations hinder precise spatiotemporal alignment and synchronous acquisition of multimodal signals, which may induce tumor localization errors and information misregistration.

Furthermore, imaging signals from different modalities originate from independent physical mechanisms with disparate signal dimensions and quantification criteria. Mature and universal multi-source signal fusion strategies and intelligent analytical models remain scarce, making it difficult to realize complementary superposition and collaborative quantitative analysis of multidimensional imaging data, thereby restricting imaging accuracy and clinical reproducibility. Additionally, tumor heterogeneity, dynamic fluctuations of the TME and variable *in vivo* metabolism of nanomaterials readily trigger signal attenuation, imaging artifacts and asynchronous multimodal responses, further compromising the precision of multimodal theranostics.

Future research should focus on optimizing spatiotemporal registration for multimodal imaging, developing intelligent signal fusion algorithms and establishing standardized quantitative frameworks. Combined with structurally engineered functional Bi-based nanomaterials, these efforts will advance multimodal imaging from simple signal superposition toward deep fusion, facilitating the clinical translation of high-precision Bi-based nanotheranostic platforms.

## 4. Multifunctional Synergistic Theranostic Integration

Conventional monotherapy for tumor treatment is restricted by tumor heterogeneity, immunosuppressive microenvironment, drug resistance and insufficient intratumoral drug accumulation. It fails to eliminate primary tumors and distant metastases, hindering the clinical translation of nanomedicines. With distinctive physicochemical properties, Bi-based nanomaterials integrate multimodal imaging and synergistic therapies. Through structural and compositional modification, they can combine phototherapy, radiotherapy, SDT and immunotherapy. Under exogenous stimulation, these materials induce tumor ICD and release damage-associated molecular patterns (DAMPs) to reverse the immune desert. Meanwhile, their TME-targeted responsiveness remodels the immunosuppressive microenvironment, achieving cascade therapeutic effects including local tumor ablation, TME regulation and systemic immunotherapy against metastasis (**Figure 18**).

In the tumor microenvironment, tumor-associated macrophages (TAMs) predominantly exhibit the tumor-promoting M2 phenotype that secretes IL-10 and TGF-β to suppress anti-tumor immune responses 132. Bismuth-based nanomaterials can reprogram TAMs toward the tumoricidal M1 phenotype via combined physical stimulation and chemical regulation 133. Under X-ray irradiation, bismuth with a high atomic number generates abundant secondary electrons to trigger ROS production, which activates the TLR4/NF-*κ*B pathway in macrophages and upregulates M1 markers including iNOS, CD86 and TNF-*α* 134-136. Bismuth semiconductors such as Bi_2_S_3_ and Bi_2_Se_3_ possess outstanding photothermal capacity, which improves tumor vascular permeability and releases DAMPs to further induce pro-inflammatory polarization of macrophages 132. PEG- or ligand-functionalized bismuth nanoparticles are readily internalized by TAMs; they can co-deliver TLR agonists and STAT6 inhibitors to block the IL-4/IL-13-driven M2 polarization pathway, and downregulate arginase-1 to remodel cellular metabolism, thereby fundamentally reversing the immunosuppressive features of macrophages 137.

On this basis, bismuth nanomaterial-triggered ICD serves as the core driver to initiate systemic adaptive immunity 138. Combined radiotherapy and photothermal therapy stimulate tumor cells to expose calreticulin (CRT) and secrete ATP and HMGB1 139. HMGB1 binds to TLR4 on dendritic cells (DC) to activate the NF-*κ*B cascade, upregulating MHC molecules and co-stimulatory CD80/CD86, facilitating DC maturation and antigen cross-presentation, and priming naive T cells into cytotoxic CD8⁺ T cells after DC migration to draining lymph nodes. ROS generated by bismuth nanomaterials elevates the immunogenicity of tumor cells for easier immune recognition 140. Intracellular ROS accumulation also upregulates NKG2D ligands on tumor cells to activate natural killer cells, coordinating innate and adaptive immunity to establish a durable systemic anti-tumor immune circuit 141.

Collectively, bismuth-based nanomaterials remodel the tumor immune microenvironment (TIME from multiple dimensions 142. They reduce the infiltration of immunosuppressive populations (M2-TAMs, regulatory T cells, myeloid-derived suppressor cells) while enriching M1 macrophages and effector T cells to dismantle immune suppression. Their enzyme-mimetic activity catalyzes endogenous H_2_O_2_ decomposition to generate oxygen and alleviate tumor hypoxia, and depletes intracellular GSH to weaken tumor antioxidant defense and amplify oxidative stress-mediated immune activation 143. Moreover, ICD induced by bismuth nanomaterials upregulates tumor PD-L1 expression, laying a theoretical foundation for combination with immune checkpoint blockad. Preclinical studies have validated that this combinatorial strategy efficiently suppresses primary tumors and inhibits distant metastasis via the abscopal effect 144, 145.

This chapter summarizes three synergistic systems: phototherapy-immunotherapy, radiotherapy-immunotherapy with TME responsiveness, and sonodynamic-phototherapy with TME responsiveness. It elaborates the structure-activity relationships of materials, clarifies molecular pathways of ICD and regulatory targets of the microenvironment, and discusses translational challenges and optimization strategies based on current studies. This work provides theoretical basis for the structural design and clinical application of multifunctional Bi-based nanotheranostics.

### 4.1 Synergistic Phototherapy and Immunotherapy

With high atomic number, unique band structure and tunable lattice defects, Bi-based nanomaterials enable the construction of non-additive cascade synergistic systems combining PTT, PDT and immunotherapy. They establish a closed-loop therapy from local tumor elimination to systemic immune activation via in-situ induction of ICD, targeted remodeling of immunosuppressive TME and cascade amplification of systemic anti-tumor immunity, overcoming the limitations of monotherapies in eradicating primary tumors and inhibiting distant metastasis 146. Narrow-bandgap Bi-based semiconductors exhibit excellent NIR photon capture capacity. Lattice relaxation generates hyperthermia for tumor ablation. Optimized structures such as heterojunctions and oxygen vacancies facilitate photogenerated carrier separation, suppress electron-hole recombination and sustain ROS production. Hyperthermia and oxidative stress trigger multiple programmed cell death including apoptosis, ferroptosis and PANoptosis. Mild photothermal effect improves intratumoral blood flow and alleviates tumor hypoxia to compensate for oxygen deficiency in PDT, thereby enhancing the intratumoral infiltration of T cells and immunotherapeutics.

These nanomaterials also possess nanozyme activity to catalyze endogenous H_2_O_2_ decomposition for oxygen generation and downregulate HIF-1*α*. Adapted to the unique TME of colorectal cancer (high H_2_S), solid tumors (high GSH and acidity), they undergo lattice dissociation and in-situ phase transformation. Fenton-like reactions continuously produce ROS, deplete GSH and inhibit GPX4 expression to aggravate tumor oxidative damage 147-149. They consume intracellular reductive substances and suppress immunosuppressive cytokines, polarizing tumor-associated macrophages from pro-tumor M2 phenotype to anti-tumor M1 phenotype and reducing immunosuppressive cells such as Tregs and myeloid-derived suppressor cells. Such effects convert immune "cold tumors" into activated “hot tumors” 150.

Tumor-associated antigens released after phototherapy, together with TME modulatory signals, jointly promote dendritic cell (DC) maturation and antigen presentation. Immuno-adjuvants modified on material surfaces cooperate with photoinduced ROS to activate the STING pathway, further boosting DC function and CD8⁺ cytotoxic T cell infiltration to trigger innate and adaptive immunity 151, 152. Tumor-associated antigens released after phototherapy, together with TME modulatory signals, jointly promote DC maturation and antigen presentation. Immuno-adjuvants modified on material surfaces cooperate with photoinduced ROS to activate the STING pathway, further boosting DC function and CD8⁺ cytotoxic T cell infiltration to trigger innate and adaptive immunity.

A single-component theranostic platform was constructed based on non-stoichiometric oxygen vacancy-rich BiFeO_3-x_ (**Figure 19A**) 65. High-concentration H_2_S in colorectal cancer lesions triggers in-situ sulfuration phase transformation: BiFeO_3-x_ converts into Bi_2_S_3_ with strong NIR absorption and simultaneously releases Fe^2^⁺. Under 808 nm laser irradiation, the system integrates enhanced photothermal effect, Fenton-like catalysis and ferroptosis induction. *In vitro* assays on HCT116 cells revealed that cell viability decreased to 20% after combined treatment with the material and laser, while it remained above 70% in single-treatment groups. Protein quantification showed that GPX4 and SLC7A11 were downregulated by 2.74-fold and 1.64-fold, respectively, along with massive accumulation of lipid peroxides. Extracellular ATP and HMGB levels increased by 3.6-fold and 2.6-fold, verifying robust ICD. In CT26 bilateral tumor-bearing mice, primary tumors were completely eradicated within 12 days in the combined treatment group, and the volume of distant tumors was only 32.89 mm^3^, much lower than those in oxaliplatin and cisplatin chemotherapy groups. CyTOF analysis demonstrated that the maturation rate of DC rose from 22.78% to 45.89%, the proportion of regulatory T cells dropped from 13.14% to 2.28%, and the percentage of M1-type macrophages increased obviously, indicating effective reversal of the immunosuppressive TME. The survival rate of treated mice reached 60% within 50 days (Figure 19**B**). Meanwhile, PA imaging dynamically monitored the intratumoral sulfuration process of the material to realize image-guided therapy. It possesses clinical potential for minimally invasive treatment of early superficial colorectal cancer.

Xiao *et al*. 98 developed a biomimetic bacterial nanocarrier (Bif@PBi-R) for targeted photothermal-immunotherapy. Porous bismuth nanoparticles loaded with TLR7/8 agonist R848 were anchored onto bifidobacterial surface via polydopamine adhesion (Figure 20A). Utilizing the anaerobic tropism of bifidobacteria, the carriers specifically colonized hypoxic regions of solid tumors and avoided systemic inflammation caused by free R848 diffusion. Under 808 nm NIR irradiation, bismuth nanoparticles exhibited a high photothermal conversion efficiency of 76.94% to generate localized hyperthermia for tumor ablation and massive release of tumor-associated antigens. The acidic TME and photothermal stimulation triggered in-situ dissociation of the carrier and site-specific R848 release. R848 activated the TLR7/8-MyD88-NF*κ*B pathway to promote DC maturation, forming a cascade synergistic effect between tumor antigens and in-situ adjuvant stimulation. *In vitro* results showed that combined treatment remarkably raised the maturation rate of bone marrow DCs to 43.62%, upregulated IL-6 secretion and sharply reduced the viability of 4T1 cells. In orthotopic and bilateral tumor-bearing mice, this strategy efficiently suppressed primary tumor growth, increased intratumoral infiltration of CD4⁺ and CD8⁺ T cells, and elevated memory T cells in the spleen. Serum pro-inflammatory factors including TNF-*α*, IFN-*β* and IL-6 were significantly upregulated (Figure 20B). The long-lasting systemic immunity effectively inhibited distant tumors and lung metastasis. Pathological and blood biochemical analyses confirmed favorable biosafety of the nanocarrier. This system possesses promising clinical prospects for superficial, early-stage metastatic triple-negative breast cancer and can serve as a minimally invasive adjuvant photothermal immunotherapeutic modality.

Another nanoplatform was constructed with metallic bismuth as the photothermal core, SiO_2_ as the interlayer, and porous COP shell co-loaded with AQ4N and paclitaxel. The outer tumor-erythrocyte hybrid membrane (BSCAPM) endowed the system with homologous targeting ability 111. NIR irradiation simultaneously activated PTT and PDT (Figure 20C). Mitochondrial damage and TOP2*α* inhibition induced intracellular Z-DNA accumulation, which further triggered ZBP1-dependent PANoptosome assembly and subsequent PANoptosis combining pyroptosis, apoptosis and necrosis, thereby greatly boosting DAMPs release. Assays on A549 and H1299 lung cancer cells demonstrated that multi-pathway cell death inhibitors only partially alleviated cellular damage, confirming PANoptosis as the dominant cell death pattern. In orthotopic and lung metastatic LLC mouse models, the formulation markedly reduced metastatic nodules in lungs, increased proportions of mature DCs and M1 macrophages as well as intratumoral CD8⁺ T infiltration, and prolonged the survival of tumor-bearing mice (Figure 20D). Other Bi-based nanosystems, including bismuth-porphyrin MOFs, 2D bismuthene, defect-modified bismuth materials and dye-doped cascade nanostructures, follow the universal design of Bi-based photothermal unit, functional modification and active components. All these agents achieve potent primary tumor inhibition and anti-metastatic immune activation *in vivo*. This design exhibits high translational potential for precision therapy of non-small cell lung cancer (NSCLC), targeting three major clinical bottlenecks of NSCLC: immunosuppressive TME, chemoresistance and pulmonary metastasis.

Lu 153 synthesized PEG-modified 2D Bi-based MOF (Bi-TCPP) via a one-step method using Bi^3^⁺ as metal nodes and TCPP porphyrin as ligands. Coordination between bismuth ions and ligands strengthens *π*-*π* stacking, narrows the band gap and optimizes carrier separation. Under 635 nm light irradiation, Bi-TCPP simultaneously generates ROS and exhibits efficient photothermal effect with a photothermal conversion efficiency of 41.5%. Moreover, Bi^3^⁺ specifically depletes GSH in TME and impairs cellular antioxidant capacity. The synergistic photothermal and PDT induces tumor cell apoptosis, necrosis and pyroptosis. Abundant DAMPs including CRT, ATP and HMGB are released to trigger ICD, which promotes dendritic cell maturation and CD8⁺ T cell infiltration, forming a cascade synergy between tumor ablation and systemic anti-tumor immunity. *In vitro*, the combination of Bi-TCPP and light irradiation reduced 4T1 cell viability to below 10%, with an anti-tumor efficacy 5.8 times higher than free TCPP, indicating non-additive synergistic cytotoxicity. In 4T1 tumor-bearing mice, the material achieved maximum tumor accumulation at 12 h post administration. The combined treatment led to remarkable regression of primary tumors, and the proportion of mature DC in the spleen increased from 17.6% to 42.2%. The established long-term immunity nearly completely suppressed lung metastasis. Additionally, Bi-TCPP showed a hemolysis rate below 5% and caused no pathological damage to major organs, demonstrating excellent biosafety.

### 4.2 Synergistic Radiotherapy-Immunotherapy and TME-Responsive Cascade Reactions

The core merits of Bi-based nanomaterials in combined radiotherapy and immunotherapy stem from their superior radiosensitization derived from high-Z bismuth, as well as TME-responsive cascades via rational engineering. This multidimensional synergy addresses hypoxia and immunosuppression in conventional radiotherapy, and converts local treatment into systemic anti-tumor immunity by inducing ICD.

Bismuth markedly enhances radiation energy absorption in tumors, generating abundant secondary electrons and ROS to cause irreversible DNA damage. Nevertheless, sole energy deposition fails to achieve optimal efficacy, since pervasive hypoxia and high GSH in TME rapidly eliminate ROS and facilitate DNA repair 154, 155. To tackle this issue, Bi-based nanomaterials are engineered as TME-responsive smart platforms. For instance, Mn-doped bismuth materials exert catalase-like activity to in-situ produce oxygen and alleviate hypoxia, while depleting excessive GSH to amplify radiation-induced DNA damage 118. Meanwhile, released Mn^2^⁺ initiates Fenton/Fenton-like reactions for robust ROS overproduction, synergizing with radiation to trigger ICD. The released cytoplasmic DNA combined with Mn²⁺ activates the cGAS-STING pathway, promotes dendritic cell maturation and CD8⁺ T cell infiltration, thereby realizing radiosensitization and TME remodeling simultaneously. The dual effects of self-oxygenation and antioxidant depletion fundamentally reverse radiotherapy resistance.

Yin *et al*. 103 fabricated BSA-modified 2D bismuthene nanozymes (BMBs) with in-situ grown manganese dioxide. The high-Z bismuth enables prominent radiosensitization, while Mn-based components possess triple POD/GPX/NDH-like activities. In acidic TME, BMBs sequentially deplete GSH and NADPH, downregulate GPX4 and FSP1 (two key ferroptosis defense pathways), and aggravate lipid peroxidation and intracellular oxidative stress (**Figure 21c**). Ferroptosis-mediated ICD links with radiation injury to form a cascade synergy of radiotherapy-ferroptosis-anti-tumor immunity. *In vitro*, BMBs plus radiotherapy dramatically increase DNA double-strand breaks and suppress GPX4/FSP expression, with lipid peroxidation elevated by 6.7-fold. In MC38 and LLC tumor-bearing mice (**Figure 21b**), the combined treatment achieves tumor inhibition rates of 70.6% and 73.2% respectively; intratumoral GSH drops by nearly 70% with elevated malondialdehyde (**Figure 21a**). The nanosystem efficiently remodels immunosuppressive TME with negligible toxicity to major organs. This nanozyme regimen perfectly addresses the clinical bottleneck of radiotherapy resistance in lung and colorectal cancers, featuring facile fabrication and favorable biosafety profiles. Ma *et al*. 156 developed virus-like hollow bismuth-manganese bimetallic oxide nanoparticles loaded with SOCS6 plasmid (SOCS6@vHMMn-Bi) (**Figure 21d**). High-Z Bi_2_O_3_ provides strong X-ray attenuation for exogenous radiosensitization. In acidic TME, MnO_2_ is reduced by GSH to release Mn^2^⁺, which triggers Fenton-like reactions to decompose endogenous H_2_O_2_. This process relieves tumor hypoxia via oxygen generation and accumulates massive ROS, while intracellular GSH depletion further exacerbates oxidative stress. Moreover, the delivered SOCS6 plasmid targets and inhibits HIF-1α and JAK2/STAT3 pathways, endogenously elevating ROS and blocking DNA damage repair, thus constructing a dual-mode radiosensitization system (**Figure 21f**). *In vitro*, SOCS6@vHMMn-Bi combined with 6 Gy X-ray significantly increases *γ*-H2AX-labeled DNA double-strand breaks and tumor cell apoptosis (**Figure 21e**). This design centers on esophageal squamous cell carcinoma (ESCC) and constructs a dual-mode radiosensitizing nanocarrier to address two predominant clinical hurdles: hypoxia-triggered radioresistance and postoperative recurrence associated with radiotherapy. In addition, *in vitro* assays verify that this system also holds radiosensitizing potential against MCF-7 breast cancer and A549 lung cancer cells.

The combination of radiotherapy and immunotherapy is far from a simple additive effect; Bi-based nanomaterials remodel the TME to achieve profound biological synergy. Radiotherapy-induced ICD releases abundant tumor-associated antigens and DAMPs including ATP, HMGB1 and calreticulin, acting as an in-situ vaccine to initiate specific immune responses 157, 158. Meanwhile, Bi-based nanomaterials eliminate immunosuppressive metabolites such as lactic acid and adenosine, and modulate suppressive immune cells like tumor-associated macrophages and myeloid-derived suppressor cells via cascade reactions 159, 160. TME reversal relieves T cell exhaustion and enhances the efficacy of immune checkpoint inhibitors (e.g., anti-PD-L1 antibodies), enabling T cells to recognize and eliminate tumor cells efficiently 161, 162. These nanomaterials also normalize tumor vasculature and improve drug permeability to boost immune cell infiltration into tumor parenchyma 163.

A research team from Shanghai Jiao Tong University developed PEG-modified pentavalent bismuth nanoplatforms (NaBiO_3_-PEG) (**Figure 22a**) 66. Triggered by acidic TME, H⁺ accelerates material hydrolysis. The valence transition from Biᵛ to Bi^3^⁺ enables spontaneous generation of hydroxyl radicals and singlet oxygen independent of exogenous stimulation, H_2_O_2_ or O_2_. Released Na⁺ increases intracellular osmotic pressure to activate caspase-1 and induce pyroptosis. ROS-mediated DNA damage further triggers apoptosis and ICD, with the secretion of CRT, HMGB1 and ATP amplifying immune signals. Intratumoral injection ablates primary tumors and inhibits distant tumor growth. Combined with anti-CTLA4, complete regression of distant tumors was observed in 4 out of 6 mice. Systemic administration endows the nanoplatform with CT imaging capability and radiosensitization. It downregulates 53BP1/RAD51 to block DNA repair and arrest tumor cells at G2/M phase, achieving remarkable tumor suppression after two intravenous injections (**Figure 22b**).

Muradova *et al*. 131 fabricated polysiloxane-based nanoparticles chelating Gd/Bi and conjugated with cRGD targeting peptides. cRGD guides the nanoparticles to tumor sites by targeting highly expressed integrins for intracellular accumulation. Under X-ray irradiation, high-Z bismuth amplifies ionization damage and elevates *γ*-H2AX levels reflecting DNA double-strand breaks. The combination upregulates HMGB1, converts "cold tumors" to "hot tumors", and promotes massive intratumoral infiltration of CD3⁺CD8⁺ T cells. Fractionated 6 Gy radiotherapy combined with this agent achieves complete tumor regression in partial mouse models and prolongs median survival, with no obvious systemic toxicity. Huang’s group synthesized TBNPs^88^ using FDA-approved bismuth subsalicylate as raw material (**Figure 22c**). Transferrin receptor-mediated transport enables the nanoparticles to cross the blood-brain barrier and target orthotopic glioma. Under X-ray exposure, bismuth enhances radiation absorption to aggravate tumor DNA damage and induce ICD, accompanied by the release of ATP, CRT and HMGB1 to strengthen anti-tumor immunity. After 4 Gy irradiation combined with TBNPs, the viability of glioma cells decreased to 33.5% *in vitro* (**Figure 22d**). Orthotopic tumor growth was markedly inhibited and animal survival was significantly prolonged. Blood routine and pathological examinations of liver and kidney confirmed excellent biosafety. Collectively, all three types of nanomaterials generate reactive species via TME-responsive cascades and amplify immune signals through ICD. They exert synergistic radioimmunotherapy effects via three pathways: radiation-induced DNA damage, pyroptosis/apoptosis, and effector T cell infiltration.

In summary, Bi-based nanomaterial-mediated radioimmunotherapy achieves full-range optimization from physical radiosensitization to biological immune activation via TME-responsive cascades. This strategy resolves hypoxia-induced radiotherapy resistance and breaks tumor immune tolerance by activating key immune pathways such as cGAS-STING, offering a potent approach to combat cancer metastasis and recurrence.

### 4.3 Synergistic Sonodynamic Therapy, Phototherapy and Immunotherapy

SDT generates ROS via US-activated sonosensitizers and features deep tissue penetration, yet its efficacy is limited by insufficient ROS production under tumor hypoxia 164. Phototherapy, including PDT and PTT, relies on photon excitation with high energy conversion efficiency, but suffers from shallow penetration and interference by skin pigments 165. Bi-based nanomaterials can respond to both US and light stimuli to achieve spatiotemporal complementarity when the two modalities are combined 166. Mechanically, ultrasonic cavitation and mechanical force increase cell membrane permeability to facilitate intracellular delivery of photosensitizers or drugs, thus enhancing phototherapeutic effects 167. Meanwhile, localized hyperthermia from photothermal effects accelerates sonochemical reactions and boosts ROS yield. For example, Bi_2_S_3_-TiO_2_ heterojunctions remarkably improve the killing effect of SDT against hypoxic tumors under photothermal assistance. The improved local microcirculation alleviates tumor hypoxia indirectly 168. Such dual-modal excitation broadens the therapeutic window and induces cancer cell apoptosis and necrosis through distinct physical mechanisms 166.

Beyond direct tumor cell elimination, the combined therapy exerts long-term effects via TME remodeling. SDT and phototherapy induce ICD to release DAMPs such as CRT and HMGB1, which promote DC maturation and tumor antigen presentation.^169^ TME-responsive cascades deplete GSH and relieve hypoxia to reverse immunosuppression and elevate cytotoxic T lymphocyte infiltration 170. Bi-based nanoplatforms can also load immune checkpoint inhibitors (e.g., anti-PD-L1 antibodies) to further immune activation, elicit an *in-situ* vaccine effect, and inhibit tumor metastasis and recurrence 171.

He *et al*. 114 synthesized Pt-decorated ternary Bi-based heteronanoparticles (**Figure 23a**). The constructed Z-scheme junction and Pt Schottky contact, together with abundant oxygen vacancies, accelerate carrier separation under US. Pt exhibits catalase-like activity to decompose intratumoral H_2_O_2_ for oxygen supply against hypoxia, while bismuth ions deplete intracellular GSH. The investigation centered on triple-negative breast cancer as the core research model, targeting two major clinical bottlenecks: compromised SDT efficacy and tumor immunosuppression arising from the hypoxic microenvironment of solid tumors. US stimulation triggers massive production of ·OH and O_2_⁻, inducing PANoptosis and ICD accompanied by DAMPs release. This facilitates DC maturation and CD8⁺ T cell infiltration, and remodels the immunosuppressive TME (**Figure 23b**). *In vivo*, combined US treatment achieves a 40% inhibition rate for primary tumors and 90% for distant tumors, effectively suppressing lung metastasis and establishing long-term anti-tumor immunity (**Figure 23c**).

Pu *et al*. 100 developed injectable ROS-responsive DBG hydrogel loaded with bismuth ferrite piezoelectric nanosheets and 2-DG (**Figure 24a**). US activates the piezoelectric effect of bismuth ferrite to split water into ·OH and O_2_⁻ and trigger ICD. Meanwhile, locally generated ROS degrade the hydrogel scaffold for sustained 2-DG release. 2-DG blocks tumor N-glycosylation to eliminate "don’t eat me" signals and reverse immune evasion, converting cold tumors into hot ones via dual pathways (**Figure 24b**). *In vivo*, the DBG plus US group significantly suppresses primary tumors, distant lesions (**Figure 24c**) and lung metastasis (**Figure 24d**), with elevated proportions of CD8⁺ T cells and mature DCs in spleens and lymph nodes. Tumor rechallenge assays confirm complete tumor resistance mediated by memory T cells. Both studies realize synergistic sonodynamic-immunotherapy via TME regulation, US-driven ROS generation and ICD activation.

The integration of phototherapy into sonodynamic-immunotherapy brings multiple benefits. Photothermal hyperthermia directly ablates tumor cells and synergistically accumulates intracellular ROS to strengthen SDT. It also downregulates HIF-1α to mitigate hypoxia and remove the oxygen dependence of SDT. Additionally, heat stress promotes the release of DAMPs including CRT and HMGB1 to amplify ICD, further facilitating DC maturation and CD8⁺ T cell infiltration and optimizing the TME for triple-modal synergistic therapy. Cao *et al*. 115 fabricated homologous Schottky heterojunctions (Bi-HJ) via defect engineering, and loaded atovaquone (ATO) to obtain ATO@Bi-HJ (**Figure 25a**). The in-situ Schottky structure and abundant oxygen vacancies narrow the bandgap and accelerate US-induced electron-hole separation, yielding abundant ^1^O_2_, ·OH and O_2_⁻. ATO inhibits mitochondrial oxygen consumption to relieve tumor hypoxia and break the oxygen limitation of SDT. Oxygen vacancies also endow Bi-HJ with prominent NIR photothermal performance (photothermal conversion efficiency: 31.2%). Combined ROS and hyperthermia induce robust ICD and DAMPs release to activate DCs and CD8⁺ T cells (**Figure 25b**). *In vivo*, the combined treatment of ATO@Bi-HJ with US and NIR irradiation achieves a tumor inhibition rate of 94.2%. The nanocomposite also possesses CT imaging capability, acid degradability and favorable biosafety. Miao’s group 91 synthesized sulfur-doped bismuth oxide (BS) via topological sulfurization, and fabricated BSM nanoformulations by loading ivermectin (IVM) (**Figure 25c**). Sulfur doping narrows the bandgap and improves electron-hole separation. The platform produces high levels of ROS under US and exhibits efficient photothermal conversion under NIR light. IVM is released in a controlled manner under dual stimulation of acidic TME and US. The triple-stimulus system potently induces ICD, promotes DC maturation and upregulates CD8⁺ T cells and pro-inflammatory cytokines (**Figure 25d**). *In vivo*, BSM combined with US and NIR irradiation nearly eradicates orthotopic 4T1 breast tumors. Both Bi-based nanosystems enhance SDT via structural optimization, and integrate photothermal ablation with ICD-mediated systemic anti-tumor immunity to suppress primary tumors and prevent immune-mediated metastasis.

The biosafety of Bi-based nanomaterials is highly dependent on their chemical composition, surface modification, particle size, and degradability under physiological conditions 45, 125. These nanomaterials follow the absorption-distribution-metabolism-excretion pathway *in vivo* 172. Unlike non-degradable nanocarriers such as gold and silica, bismuth-based compounds undergo self-sacrificial dissolution in the acidic tumor microenvironment or low-pH lysosomes, releasing Bi^3^⁺ and ligand ions 173. The liberated bismuth ions readily bind thiol-containing substances including glutathione and proteins to form complexes, which are primarily excreted via urine through the kidneys with minor biliary-fecal elimination; this clearance route effectively prevents long-term accumulation in the reticuloendothelial system 172. Although short-term experiments verify favorable biocompatibility of bismuth-based materials, their long-term toxic risks cannot be ignored. Retention of nanoparticles in the liver and spleen may trigger chronic inflammation, oxidative stress and even tissue fibrosis. While the ROS generated by such materials can specifically eliminate tumor cells, unbalanced oxidative stress may damage the mitochondria, cell membranes and DNA of normal cells, thereby inducing genotoxicity and reproductive toxicity. Moreover, their toxicity exhibits dose- and time-dependent characteristics. Long-term high-dose exposure leads to accumulation of degradation products in the kidneys and subsequent renal tubular injury. Surface charge and grafted ligands also markedly modulate material immunogenicity; unmodified nanoparticles tend to form protein coronas, which facilitate phagocytosis by macrophages and further elicit foreign body reactions and granulomatous lesions 174, 175.

Current research on the long-term biological fate and toxic mechanisms of bismuth-based nanomaterials has multiple limitations. *In vivo* studies lack long-term metabolic tracking data, and the potential hazards of trace accumulation of long-half-life bismuth isotopes and non-degradable components in deep bone tissues remain unclear 176. Bismuth ions and other metal ions released from multi-element doped bismuth nanocomposites exert synergistic or antagonistic toxic effects, yet their *in vivo* interaction mechanisms have not been systematically investigated, which may lead to uncharacterized toxic outcomes 135. Most existing toxicity data are derived from healthy animal models. Cancer patients often suffer from impaired hepatic and renal function as well as immune disorders, which drastically alter the clearance rate and *in vivo* tolerance threshold of nanomaterials, whereas current safe-by-design strategies fail to fully incorporate these pathological factors 177. In addition, there is no unified standard for administration dosages, exposure routes and detection indicators across laboratories, making cross-study data comparison and systematic safety analysis infeasible due to the scarcity of high-quality long-term experimental datasets 178. Inter-patient and intra-tumor heterogeneity (e.g., uneven hypoxia or acidity distribution) may affect the stability of *in vivo* therapeutic effects of tumor microenvironment-responsive smart materials.

## 5. Conclusion

This review systematically summarizes structural design strategies, multifunctional synergistic mechanisms and biomedical advances of Bi-based nanomaterials for precise tumor theranostics, and analyzes structure-performance merits of four mainstream nanotheranostic systems: elemental/multimetallic composite Bi-based nanomaterials, non-heterojunction core-shell coated composites, typical Bi-based heterojunction nanomaterials, and Bi-based/containing metal-organic framework nanomaterials. Endowed with high atomic number, tunable band structure, superior PA/catalytic performance and controllable biodegradability, these materials overcome clinical drawbacks of conventional tumor therapies including low precision, single treatment modality, frequent drug resistance and severe side effects. Rational structural engineering via defect modulation, interface regulation and functional integration enables multimodal imaging (CT, MRI, PA) for early tumor diagnosis, lesion localization and real-time therapeutic monitoring. Combined with PTT, radiotherapy, SDT and immunotherapy, they trigger multiple cell death pathways and remodel immunosuppressive TME, achieving synergistic effects of local tumor inhibition and systemic anti-metastasis. Bi-based nanomaterials are promising next-generation intelligent platforms for tumor theranostics.

Despite their excellent anti-tumor potential, multiple challenges hinder clinical translation. Major bottlenecks include poor therapeutic durability, heavy reliance on external stimulation, limited deep tumor penetration and high recurrence risk. Deficiencies in biosafety lie in uncontrollable degradation, unstable composite structures, potential *in vivo* accumulation toxicity and non-specific damage. These materials also suffer from unsatisfactory targeting performance dominated by passive accumulation, weak deep-tissue retention and obvious off-target effects. Additionally, insufficient immune activation fails to effectively reverse "cold tumor" phenotypes, accompanied by immune cell exhaustion and weak anti-tumor immune memory, leading to limited efficacy against advanced and metastatic tumors. Low synthesis reproducibility, immature large-scale fabrication and inadequate long-term *in vivo* mechanism research further impede clinical application.

Future research shall focus on precision, intelligence, safety and long-term efficacy to bridge fundamental innovation and clinical translation.

(1) Optimize microenvironment responsiveness, deep penetration and theranostic stability via defect engineering, bandgap modulation, interface modification and topological reconstruction, to develop low-dose, high-efficiency and high-specificity nanoplatforms against shallow treatment depth, transient efficacy and tumor recurrence.

(2) Biomimetic membrane coating and amino acid-based degradable polymer hybridization enable the fabrication of Bi-based architectures with spatiotemporally controllable degradation. The development of renal-clearable ultrasmall bismuth heterostructures and topologically reconstructed 2D bismuthene derivatives further achieves fully biodegradable and bioeliminable bismuth nanosystems.

(3) Advance targeted delivery strategies by integrating targeting peptides, specific antibodies, cell membrane bionics and anaerobic bacteria targeting to cross physiological barriers, enabling precise accumulation in primary tumors, deep lesions and micrometastases while reducing off-target toxicity.

(4) Multimodal imaging real-time monitors nanomaterial accumulation, tumor heterogeneity and lesion microenvironments. Combined with AI-assisted radiomics, therapeutic and external stimulation parameters are dynamically optimized according to individual tumor characteristics and drug resistance, enabling precise personalized tumor intervention.

(5) The interdisciplinary integration of Bi-based nanomaterials with CAR-T therapy and mRNA tumor vaccines enhances the infiltration, survival and cytotoxicity of CAR-T cells. Such nanoplatforms facilitate *in situ* mRNA vaccine expression to amplify specific anti-tumor immunity, establishing an innovative theranostic paradigm integrating conventional combination therapy with advanced immunotherapy.

## Figures and Tables

**Figure 1:**
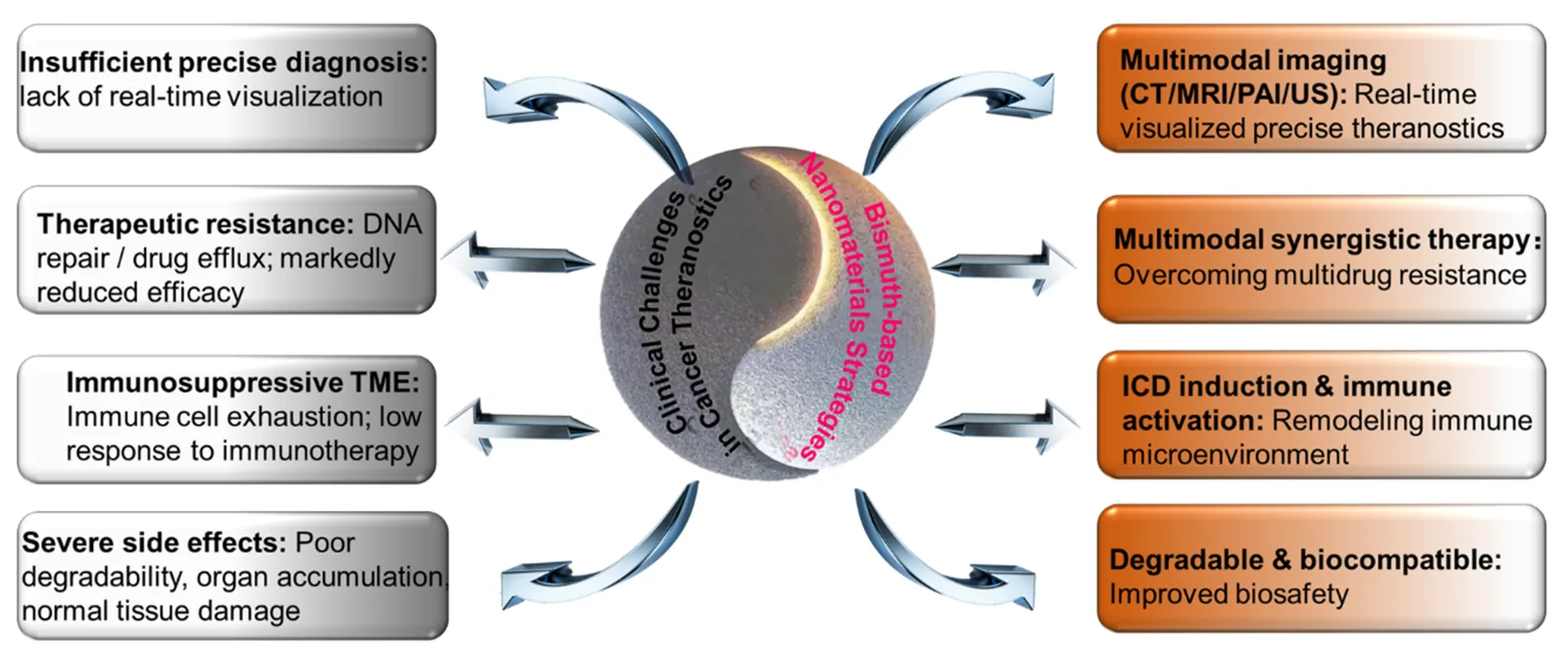
Clinical challenges of cancer theranostics and corresponding solutions enabled by bismuth-based nanomaterials. (Created with Microsoft PowerPoint).

**Figure 2:**
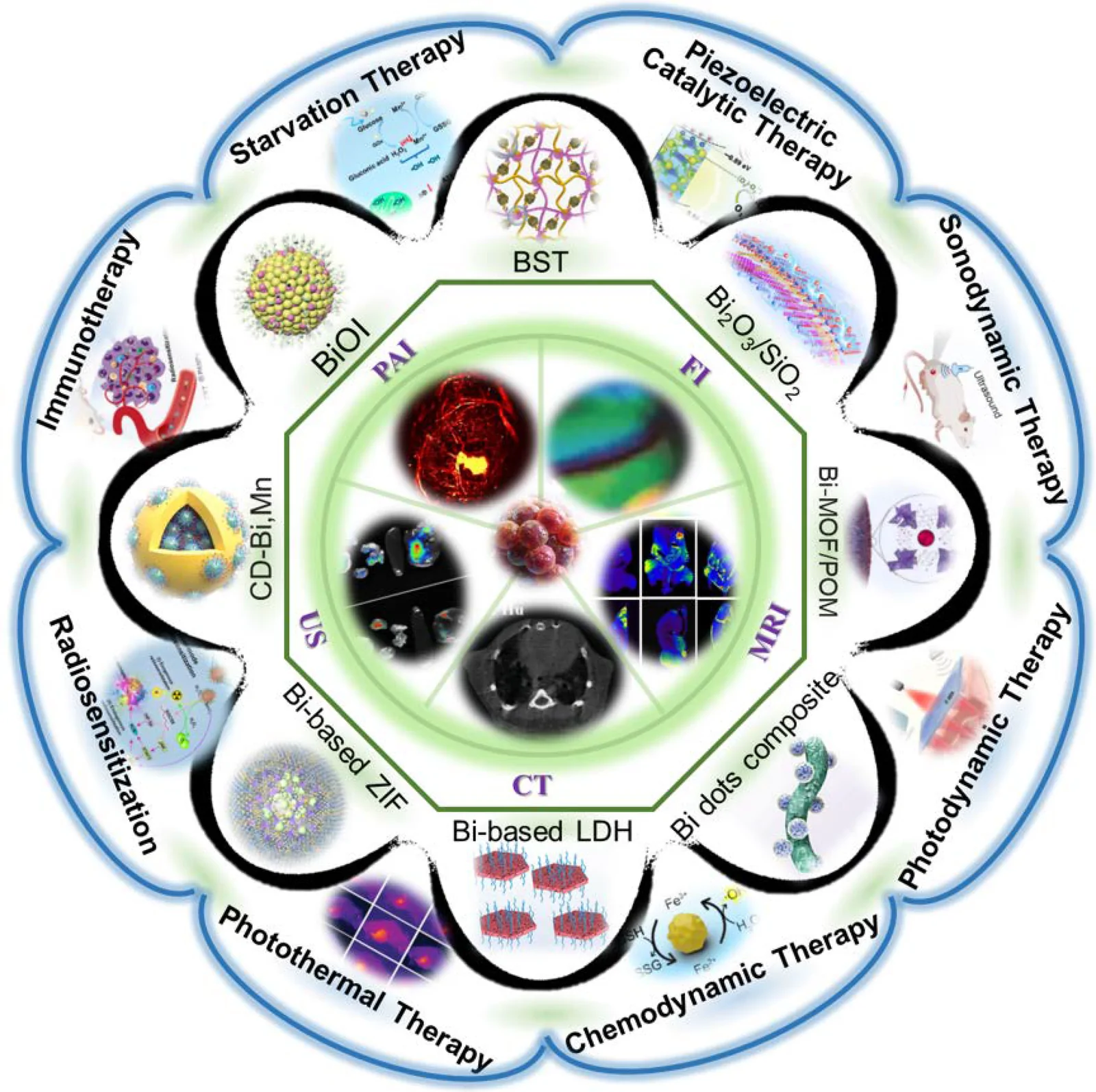
Bismuth-based nanomaterials represent a class of highly investigated nanoplatforms for tumor theranostics. In existing studies, multi-dimensional structural design and functional modification of bismuth-based nanomaterials can construct imaging systems for single-modal or multimodal synergistic imaging, including photoacoustic imaging, fluorescence imaging, magnetic resonance imaging, CT imaging and ultrasound imaging. Meanwhile, relying on their specific responsive properties toward the tumor microenvironment, bismuth-based nanomaterials can mediate multiple therapeutic modalities such as radiotherapy, immunotherapy, sonodynamic therapy and photodynamic therapy, so as to achieve monotherapies or combined synergistic therapies against tumors. (Created with Microsoft PowerPoint).

**Figure 3:**
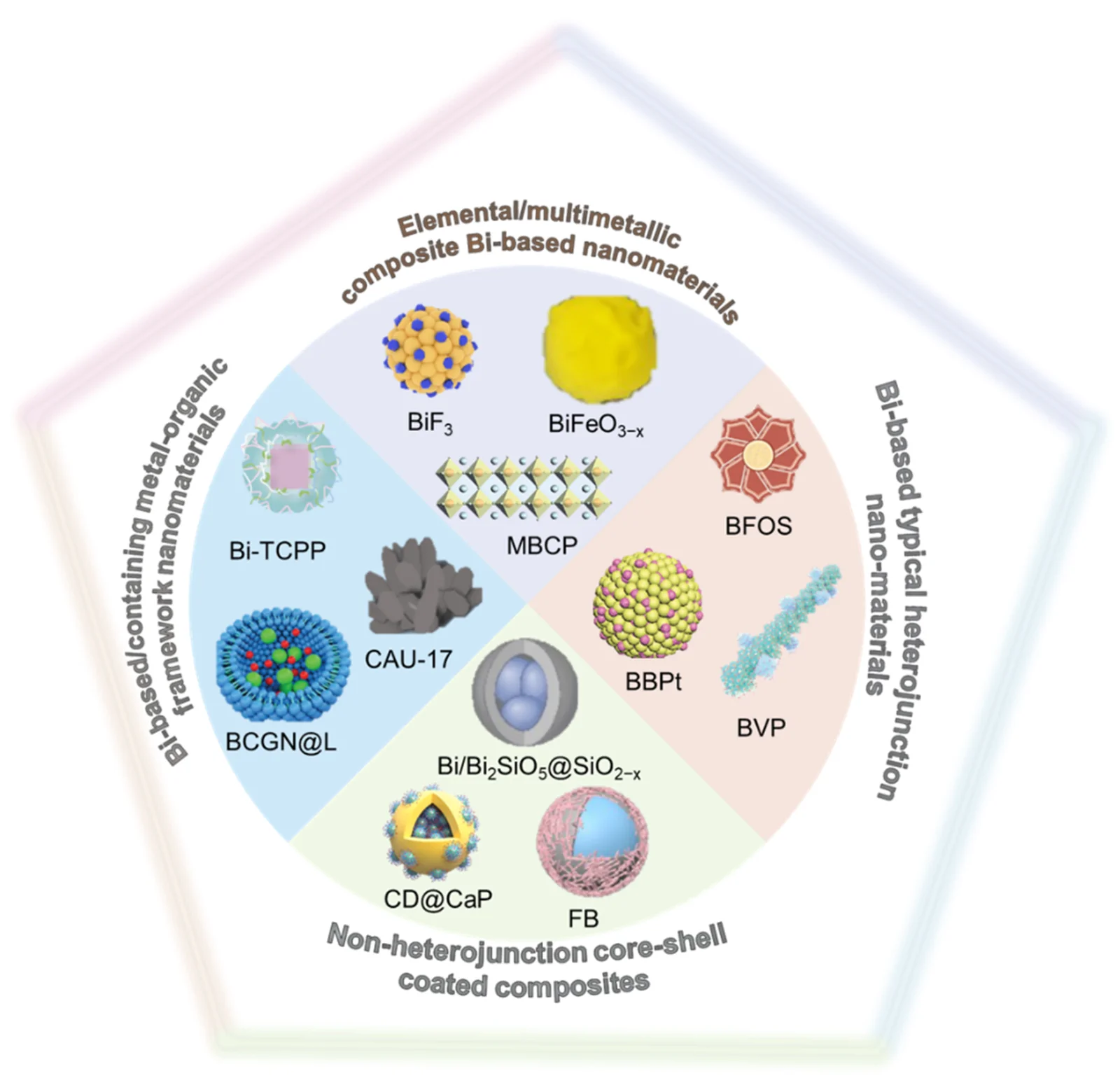
Based on current research progress, bismuth-based nanomaterials can be categorized into four structural systems: elemental/multimetallic composite Bi-based nanomaterials, non-heterojunction core-shell coated composites, typical Bi-based heterojunction nanomaterials, and Bi-based/containing metal-organic framework nanomaterials. (Created with Microsoft PowerPoint).

**Figure 4:**
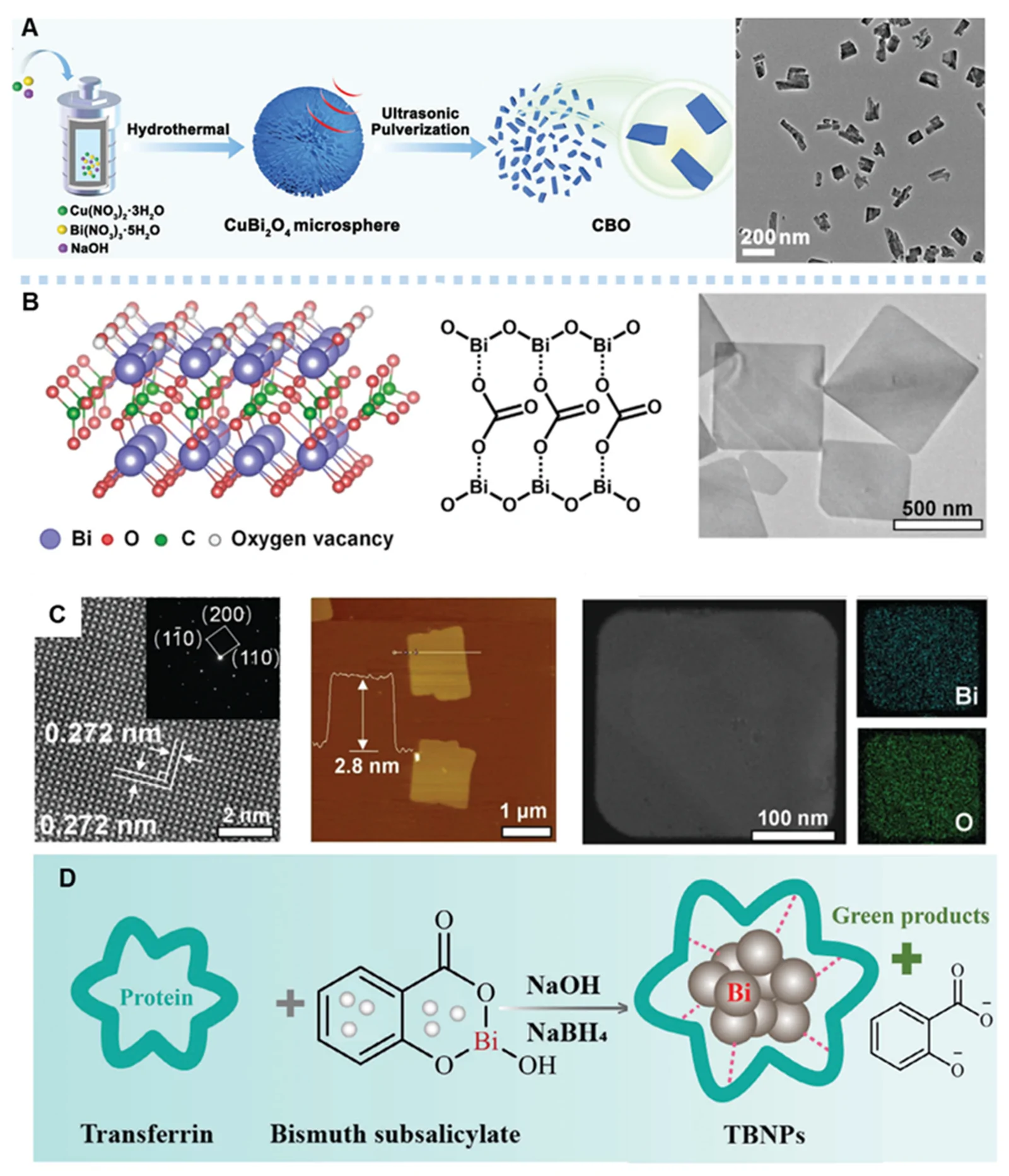
(A) Synthesis and characterization of novel PCBO. Adapted with permission from 73 Copyright 2024, Wiley-VCH GmbH. (B) Schematic illustration of crystal structure, chemical structure and TEM image of the nanosheets. (C) HRTEM image, AFM image and HAADF image of the nanosheets. Adapted with permission from 25 Copyright 2024, Wiley-VCH GmbH. (D) Design and synthesis process of TBNPs. Adapted with permission from 88 Copyright 2025, Wiley-VCH GmbH.

**Figure 5:**
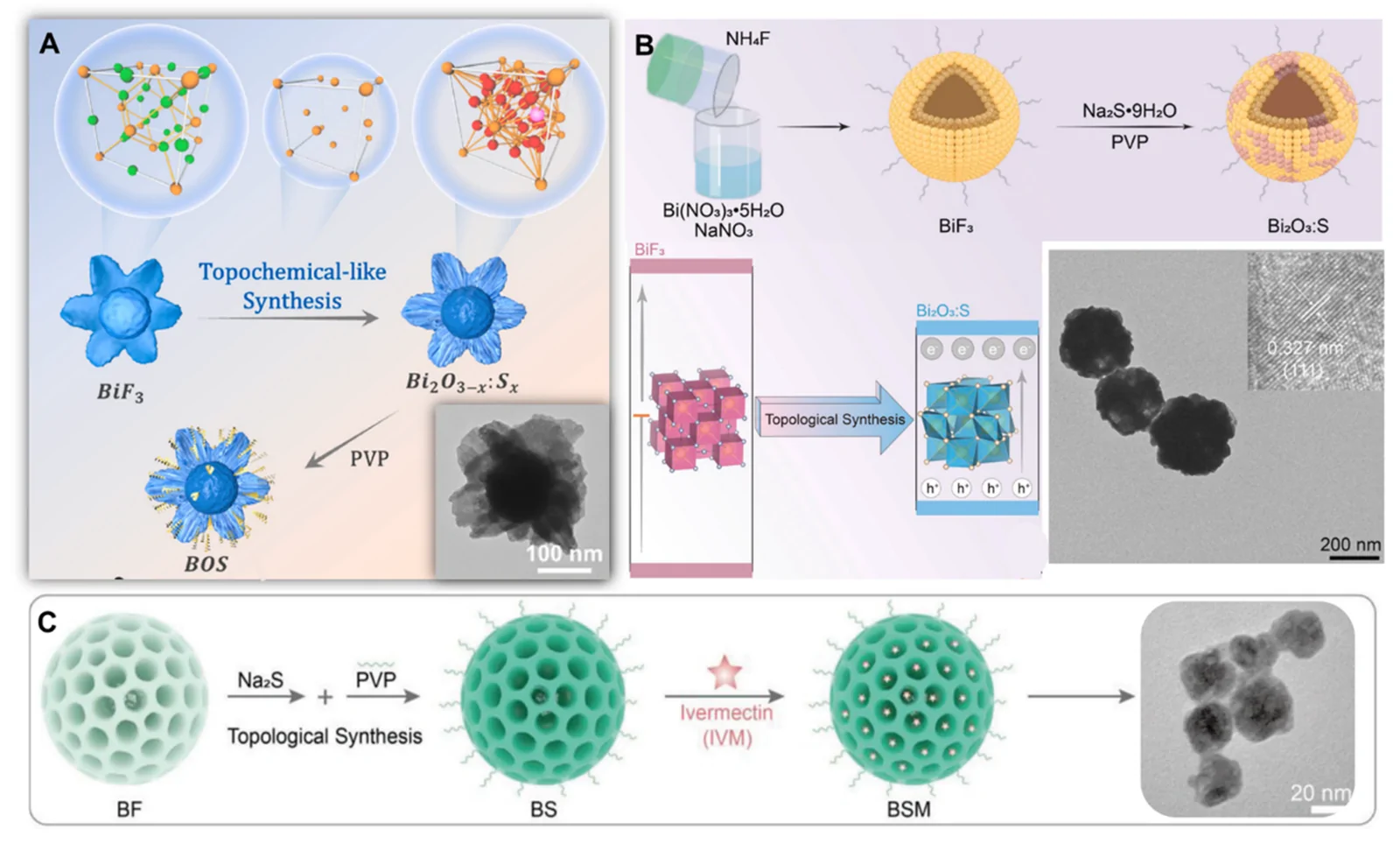
Schematic illustration of (A) flower-like, (B) Spherical, and (C) Mesoporous. (A) Adapted with permission from 89, Copyright 2024, Elsevier Inc. (B) Adapted with permission from 90, Copyright 2023, Elsevier Inc. (C) Adapted with permission from 91, Copyright 2023, Wiley-VCH GmbH.

**Figure 6:**
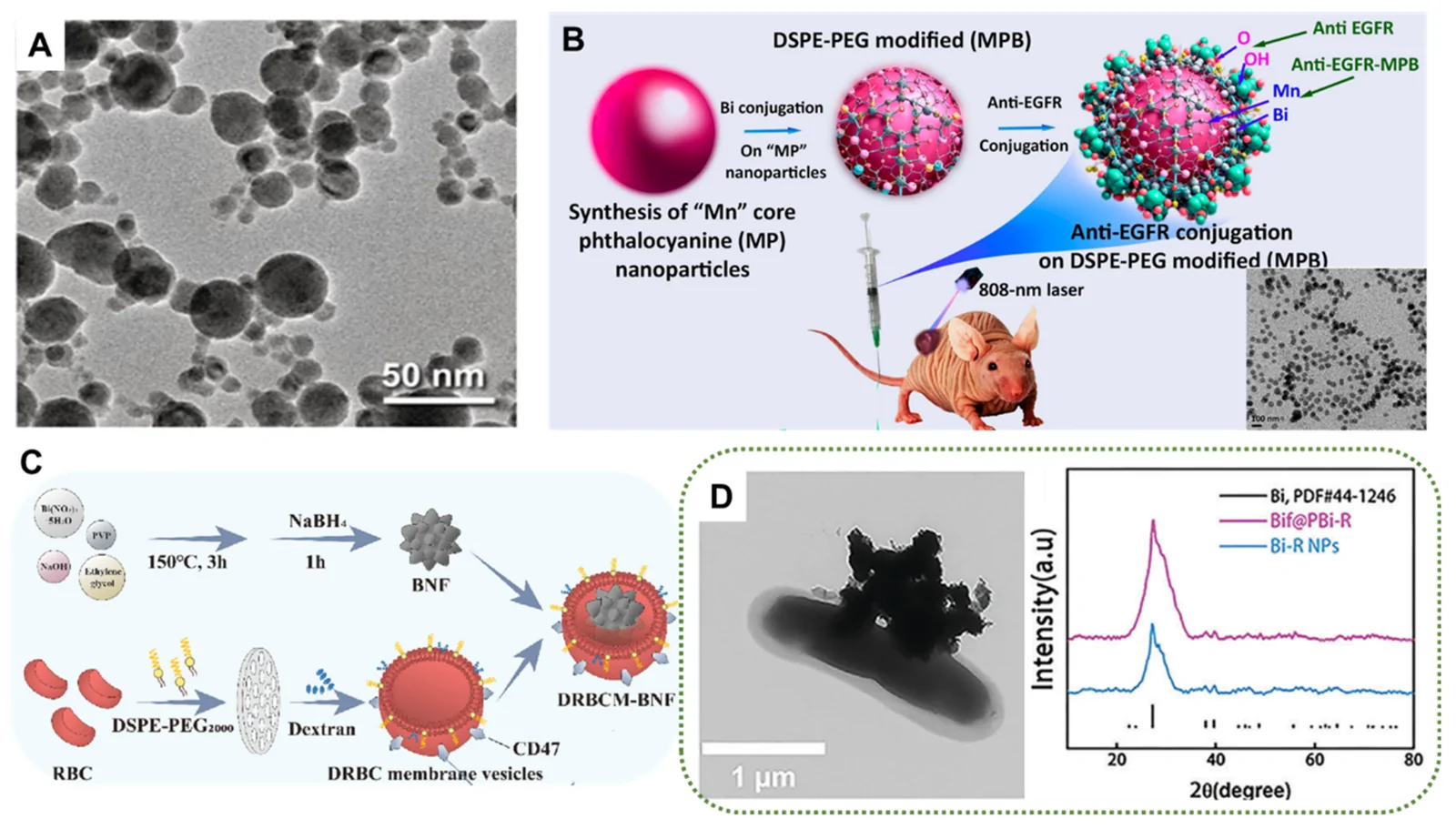
(A) Transmission electron microscopy image of laser-synthesized Bi NPs. Adapted with permission from 71 Copyright 2024, Elsevier. (B) Schematic representation of anti-EGFR-MPB nanocomposite. Adapted with permission from 96 Copyright 2024, Sudip Mondal *et al*. (C) Schematic illustration of the DRBCM-BNF. Adapted with permission from 97 Copyright 2023, Elsevier. (D) TEM images and XRD patterns of Bif@PBi-R. Adapted with permission from 98 Copyright 2024, Wiley-VCH GmbH.

**Figure 7:**
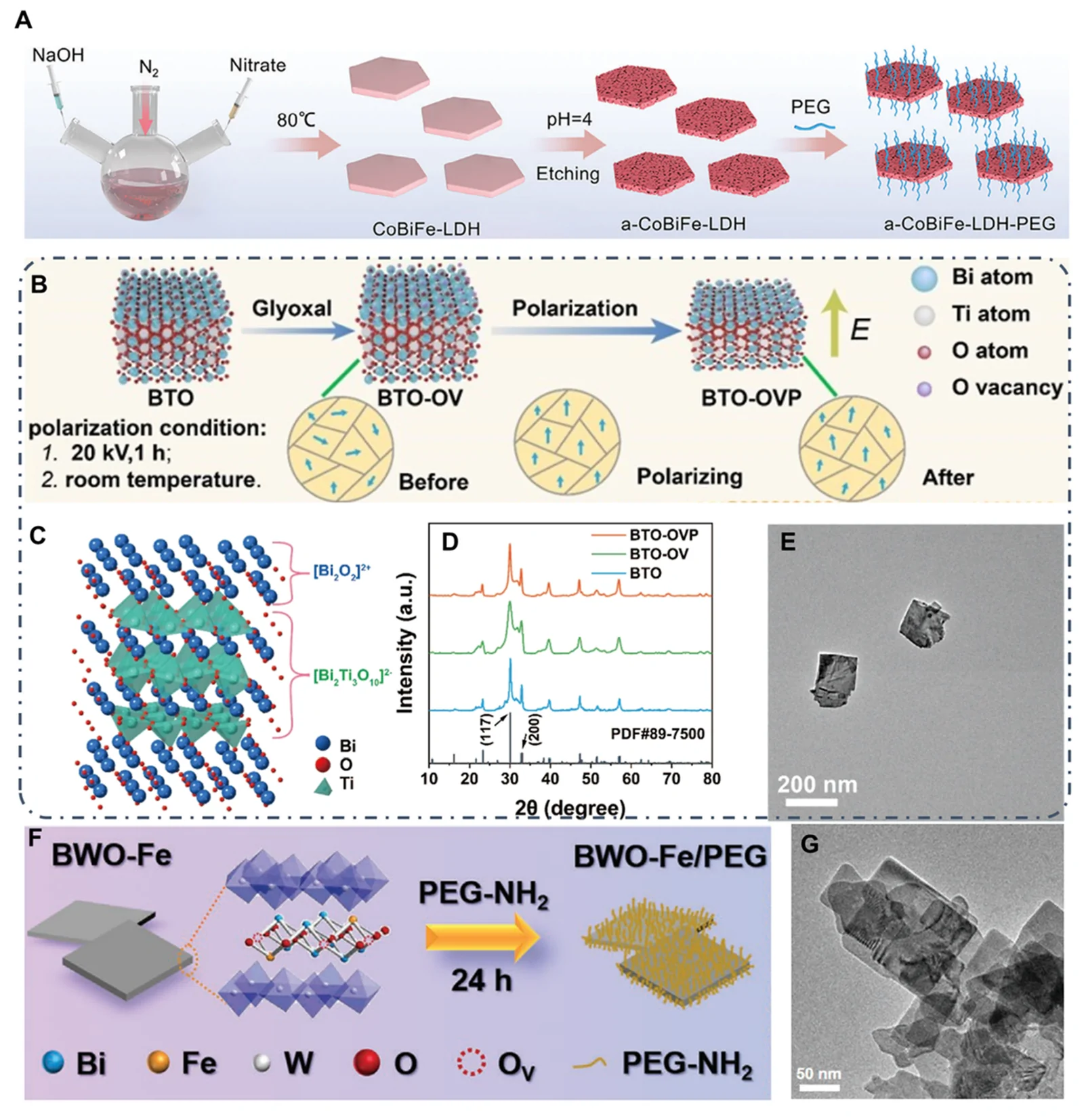
(A) Schematic illustration of the preparation of a-CoBiFe-LDH-PEG nanosheets. Adapted with permission from 99, Copyright 2024, Wiley-VCH GmbH. (B) Schematic Diagram of Surface Oxygen Vacancies and Corona Polarization of Bi_4_Ti_3_O_12_ Nanosheets. (C) Crystal structure image of BTO. (D) XRD analyses of BTO, BTO-OV, and BTO-OVP. (E) TEM image of BTO-OV nanosheets. Adapted with permission from 87, Copyright 2024, American Chemical Society. (F) Schematic diagram of the synthetic procedure of BWO-Fe/PEG NSs. (G) TEM image of BWO-Fe/PEG NSs. Adapted with permission from 101, Copyright 2023, Wiley-VCH GmbH.

**Figure 8:**
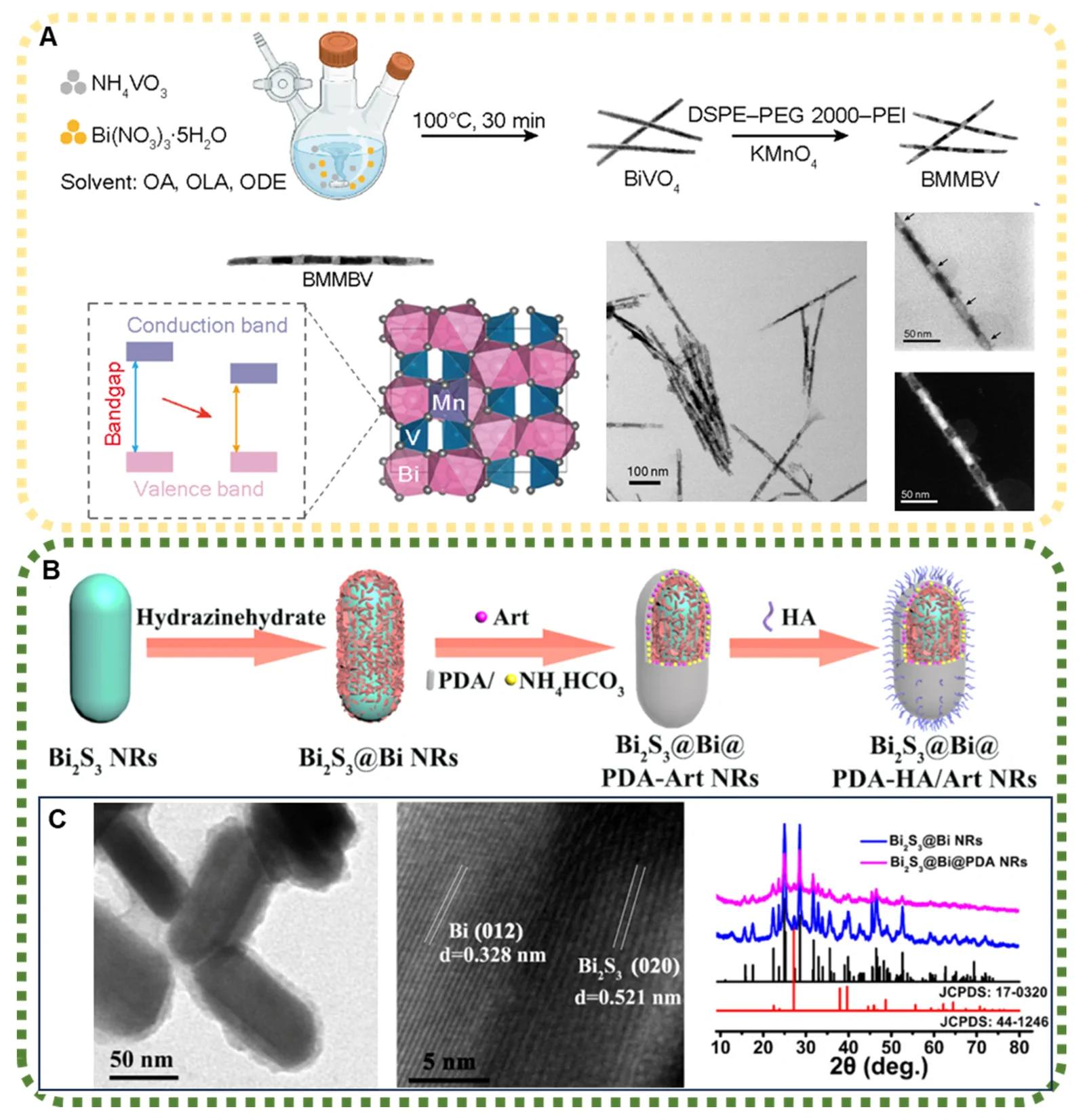
(A) Schematic diagram of the synthesis process and micromorphology images of BMMBV. Adapted with permission from 106, Copyright 2025, American Association for the Advancement of Science. (B) Schematic illustration of the preparation of Bi_2_S_3_@Bi@PDA-HA/Art NRs. (C) TEM image, HRTEM lattice fringes and XRD patterns of Bi_2_S_3_@Bi@PDA NRs. Adapted with permission from 107, Copyright 2023, Shenyang Pharmaceutical University. Published by Elsevier B.V.

**Figure 9:**
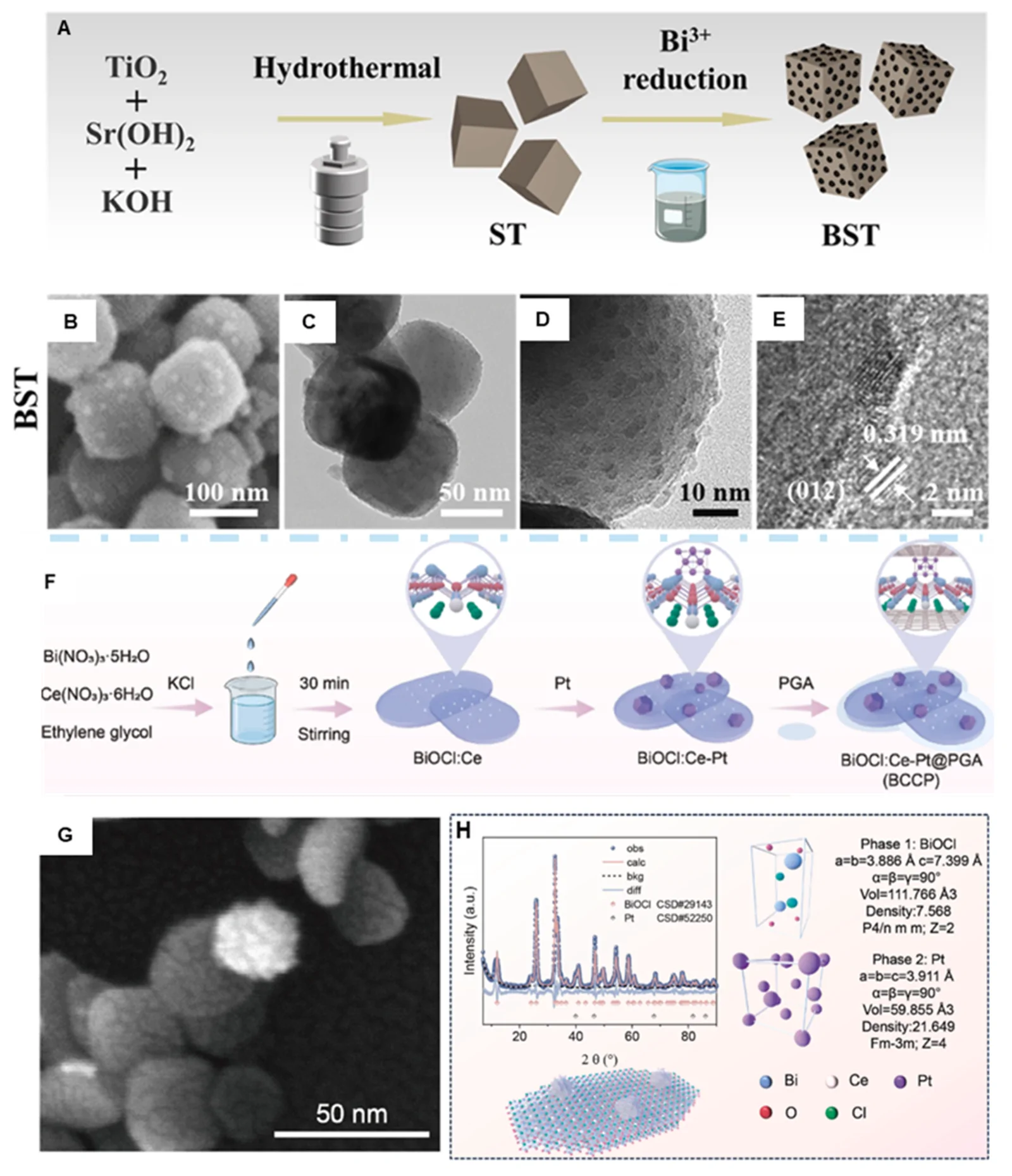
(A-E) Fabrication process and characterizations of BST NPs. Adapted with permission from 108, Copyright 2024, Elsevier B.V. (F, G) Schematic illustration of the synthesis and characterizations of BCCP. (H) XRD pattern and refined crystal structures of BiOCl and Pt. Adapted with permission from 109, Copyright 2025, Elsevier B.V.

**Figure 10:**
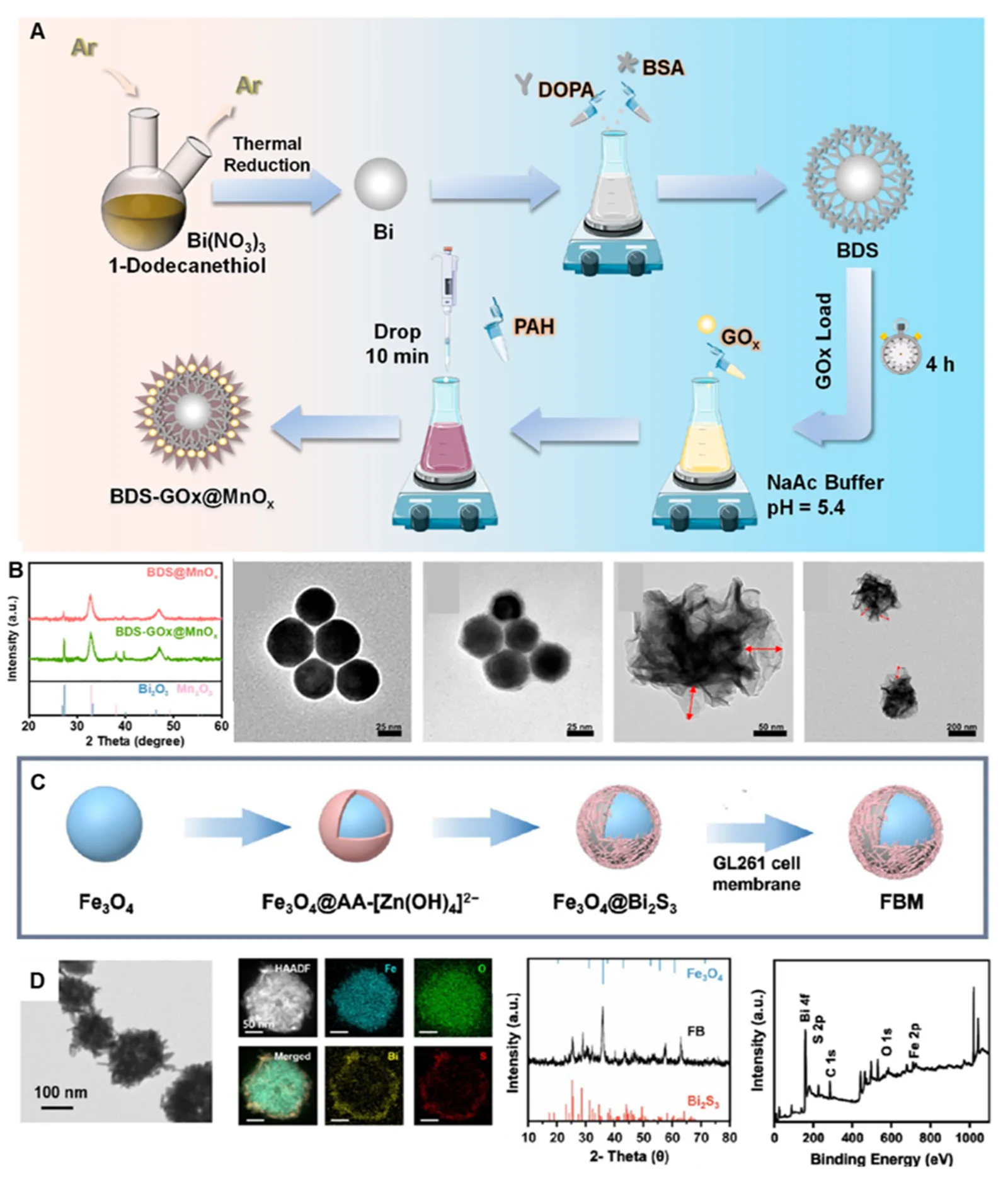
(A, B) Synthesis procedure and characterizations of BDS-GO_x_@MnO_x_. Adapted with permission from 110, Copyright 2023, American Chemical Society. (C, D) Preparation procedure and characterizations of FBM. Adapted with permission from 68, Copyright 2025, American Chemical Society.

**Figure 11:**
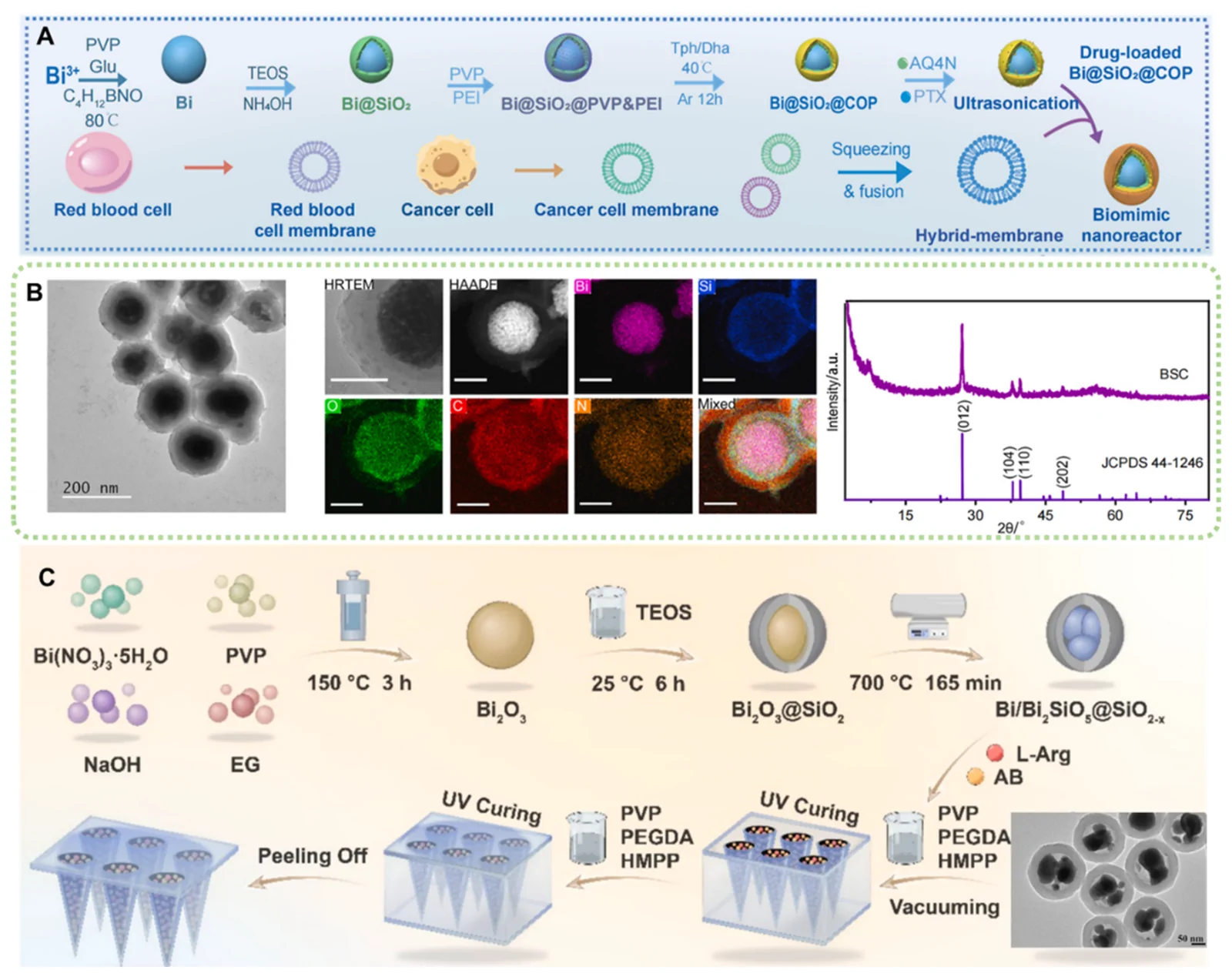
(A, B) The synthetic process and characterizations of BSCAPM nanoparticles. Adapted with permission from 111, Copyright 2025, Elsevier Ltd. (C) Schematic diagram of the Bi/Bi_2_SiO_5_@SiO_2-x_-based microneedle synthesis and TEM image. Adapted with permission from 112, Copyright 2025, Elsevier Inc.

**Figure 12:**
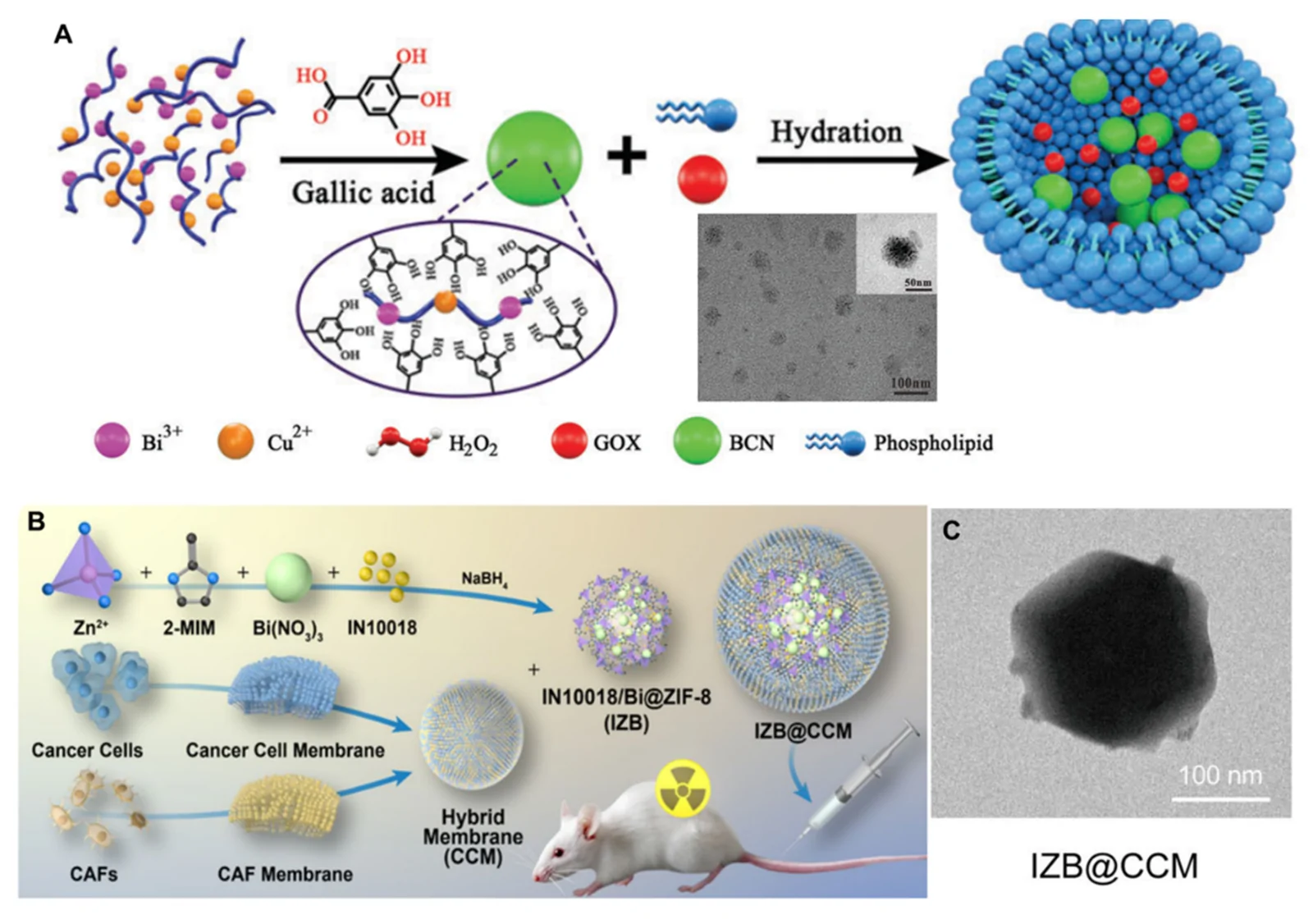
(A) Schematic illustration and TEM image of the synthesis route of BCGN@L. Adapted with permission from 113, Copyright 2023, Wiley-VCH GmbH. (B) Schematic Illustration of IZB@CCM Construction. (c) TEM image of IZB@CCM. Adapted with permission from 70, Copyright 2025, Springer Nature.

**Figure 13:**
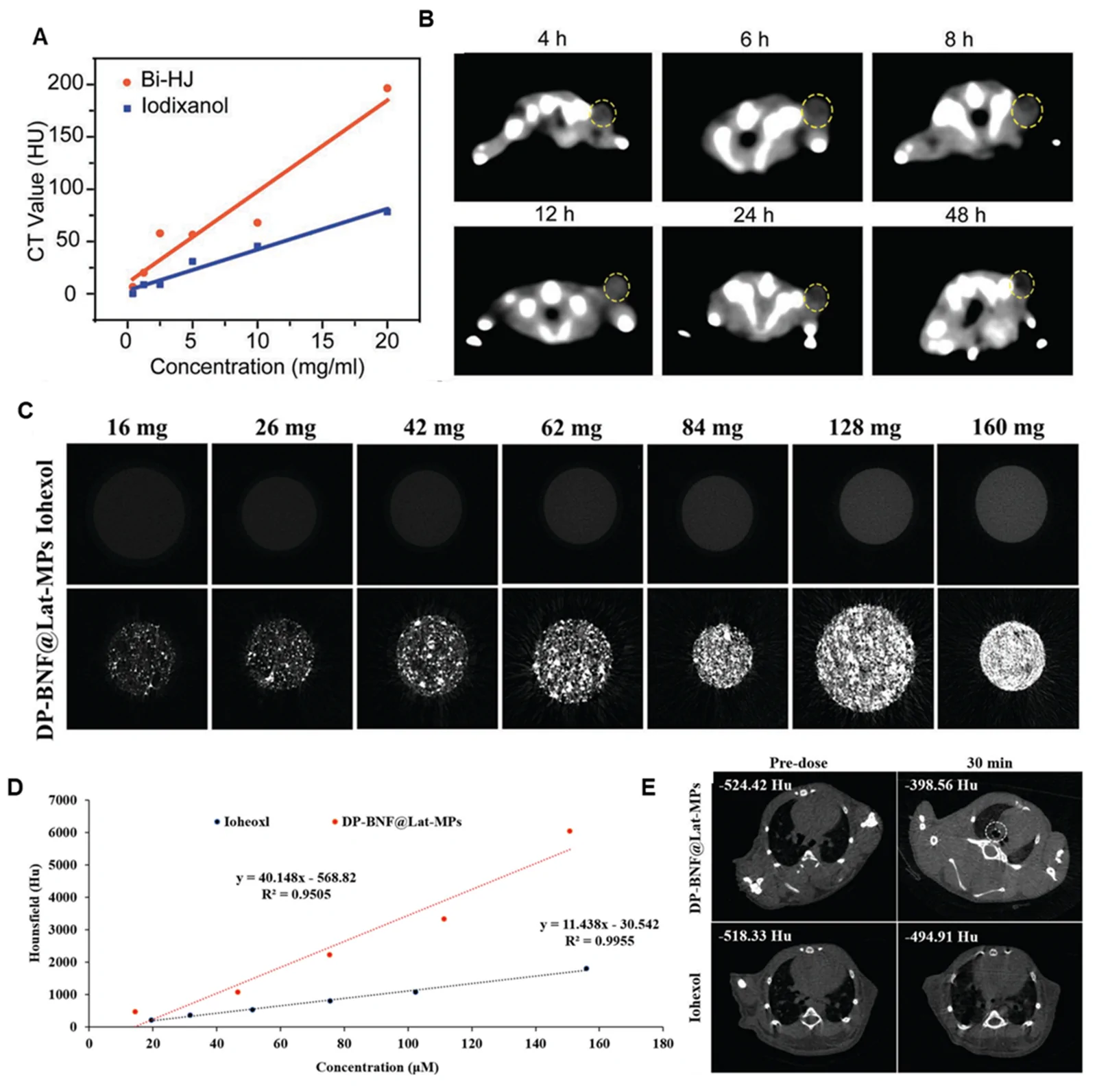
(A) Fitted curve of average CT intensity values of Bi-HJ and Iodixanol at different concentrations. (B) *In vivo* CT images captured at 4, 6, 8, 12, 24, and 48 h after intravenous administration of Bi-HJ (2 mg kg^-1^). Adapted with permission from 115, Copyright 2023, Wiley-VCH GmbH. (C) The *in vitro* CT images of iohexol and DP-BNF@Lat-MPs with different concentrations. (D) Quantification of CT intensity versus concentrations of iohexol and DP-BNF@Lat-MPs. (E) *In vivo* CT images and Hu value of mice before administration and 30 min after insufflated iohexol and DP-BNF@Lat-MPs. Adapted with permission from 116, Copyright 2023, Wiley-VCH GmbH.

**Figure 14:**
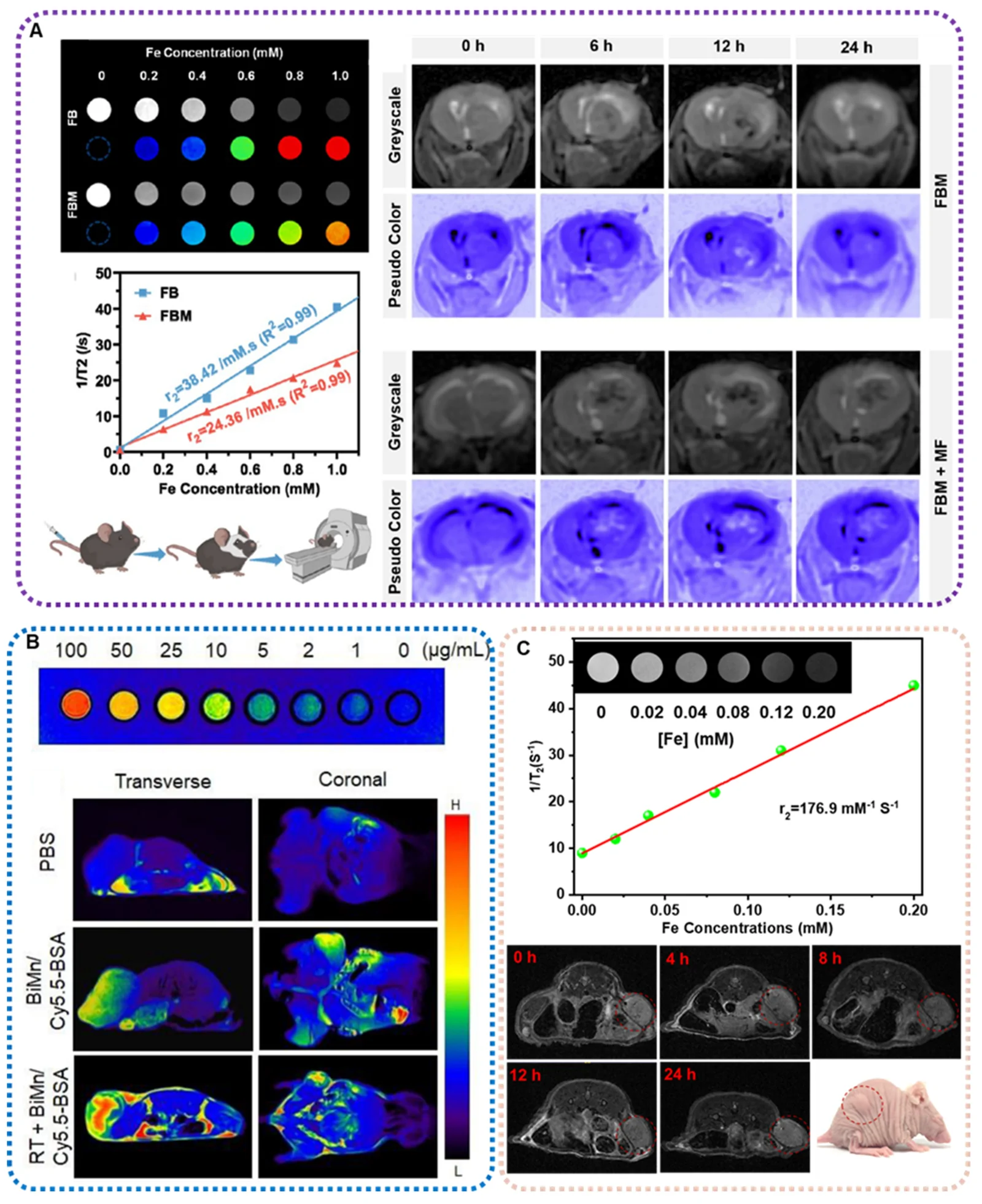
(A) MRI characterization and *in vivo* targeted imaging of FB and FBM nanoparticles. Adapted with permission from 68, Copyright 2025, American Chemical Society. (B) T_1_-weighted contrast enhanced MRI images of different concentrations of BiMn/BSA into PCR tubes, and mouse tumor MRI T_1_ images of tumors after different treatment. Adapted with permission from 76, Copyright 2024, Elsevier B.V. (C) MRI characterization and *in vivo* T_2_-weighted imaging of a-CoBiFe-LDH-PEG nanosheets. Adapted with permission from 99, Copyright 2024, Wiley-VCH GmbH.

**Figure 15:**
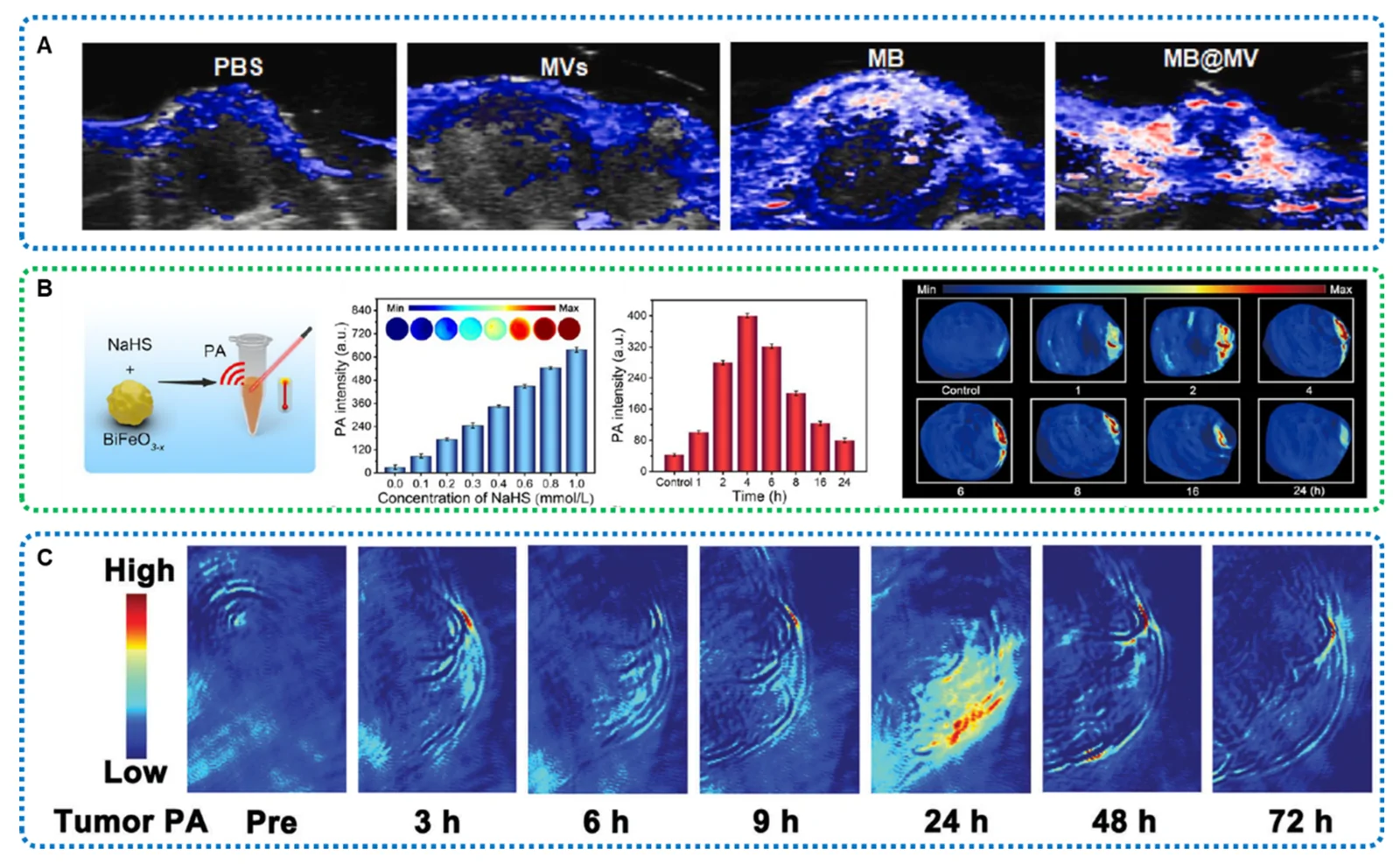
(A) Representative tumoral PA images after different treatments. Adapted with permission from 118, Copyright 2025, Bentham Science Publishers B.V. (B) HS⁻-activatable PTT and PA imaging performance of BiFeO_3-x_. Adapted with permission from 65, Copyright 2026, American Chemical Society. (C) PA images of the tumor-bearing mice treated with PCBO. Adapted with permission from 73, Copyright 2024, Wiley-VCH GmbH.

**Figure 16:**
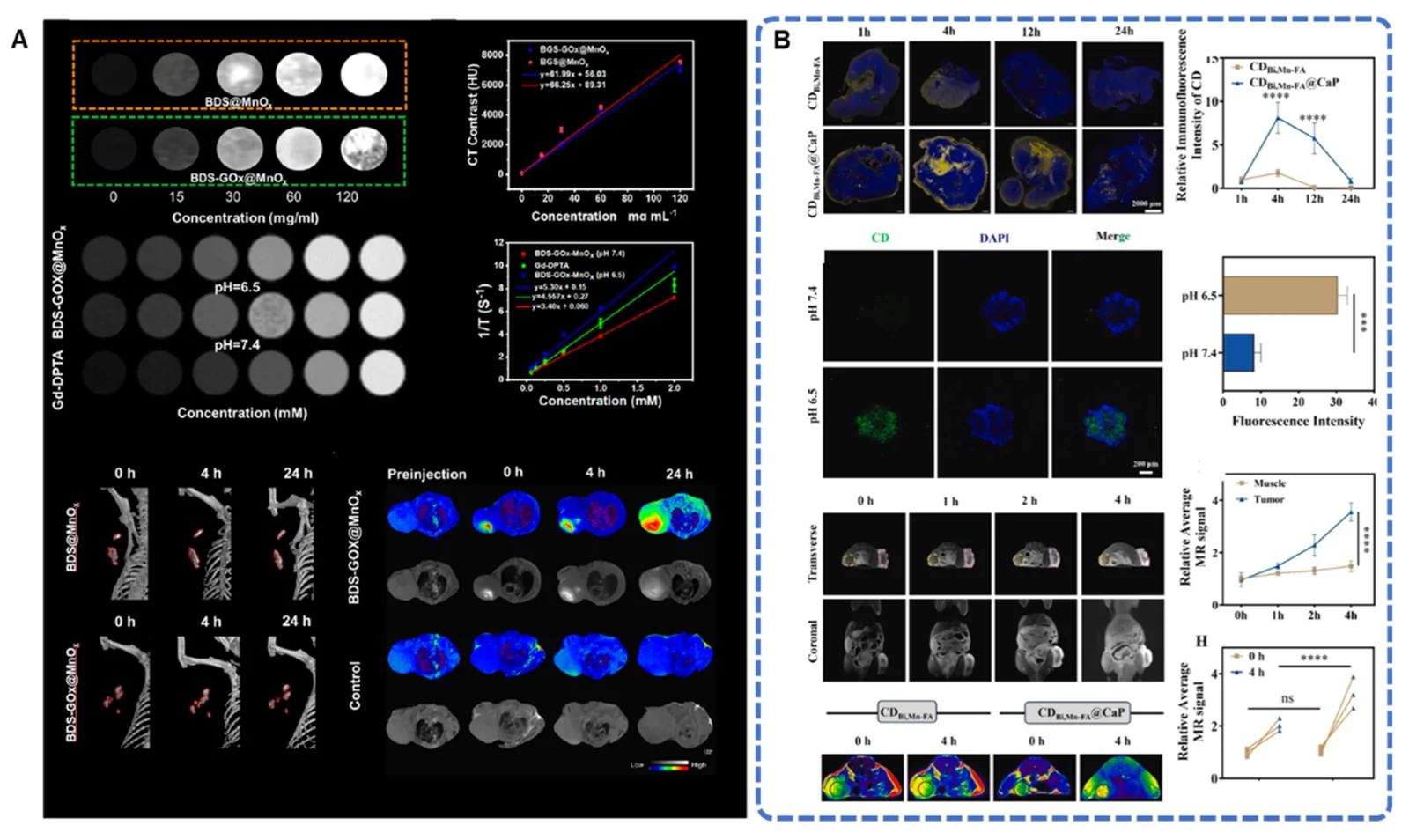
(A) CT and T1-weighted MRI enabled by BDS-GO_x_@MnO_x_. Adapted with permission from 110, Copyright 2023, American Chemical Society. (B) Tissue immunofluorescence staining to evaluate the penetration and retention of CDBi,Mn-FA and CDBi,Mn-FA @CaP and *In vivo* T_1_-weighted MRI in mice at various time points following the injection of CDBi,Mn-FA. Adapted with permission from 129, Copyright 2024, Elsevier B.V.

**Figure 17:**
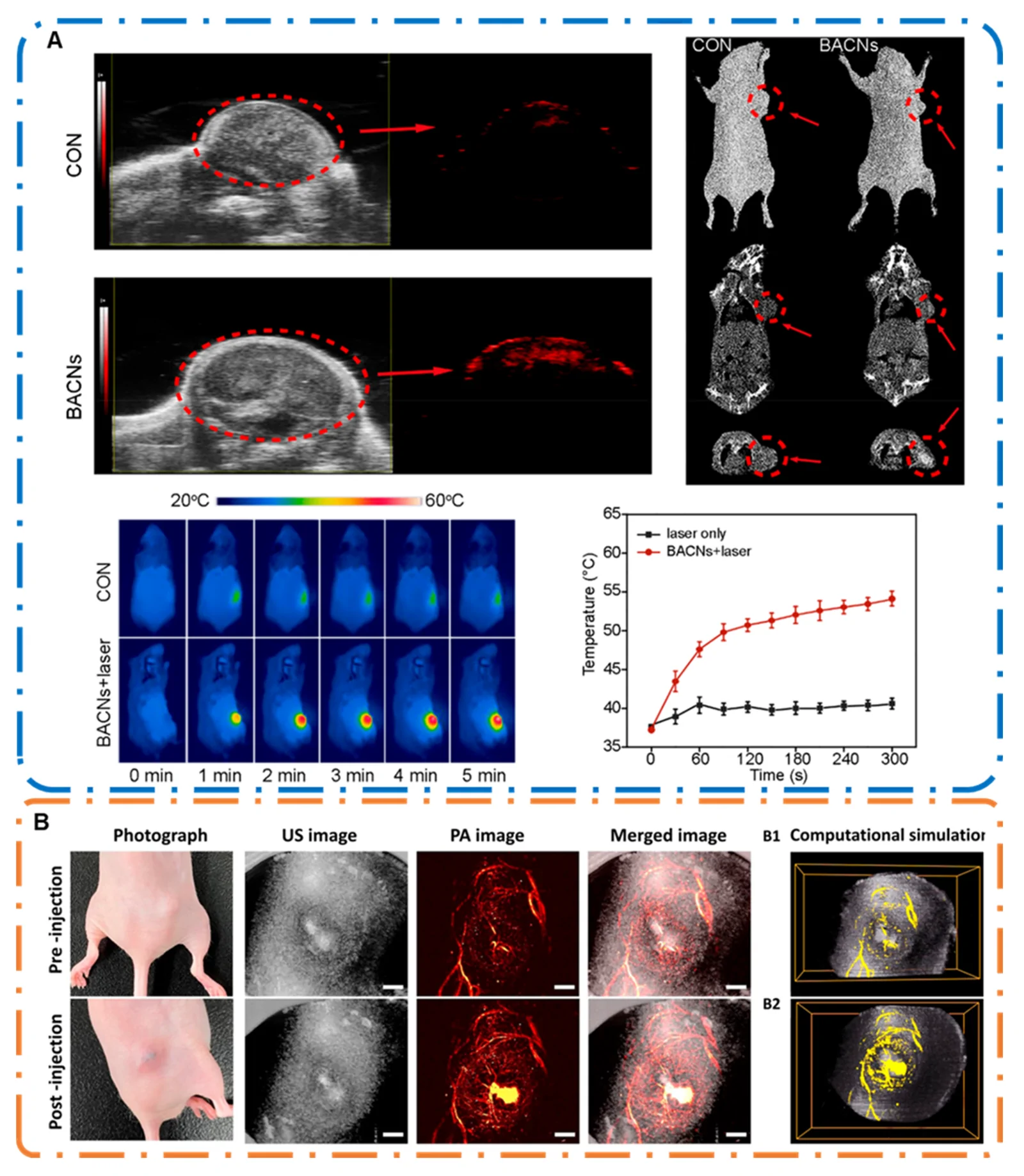
(A) CT/PA/IR imaging of tumor cells by BACNs *in vivo*. Adapted with permission from 130, Copyright 2023, American Chemical Society. (B) *In vivo* 3D PAI of tumor tissues of nude mice before and after the injection of anti-EGFR-MPB nanocomposite. Adapted with permission from 96, Copyright 2024, Elsevier B.V.

**Figure 18:**
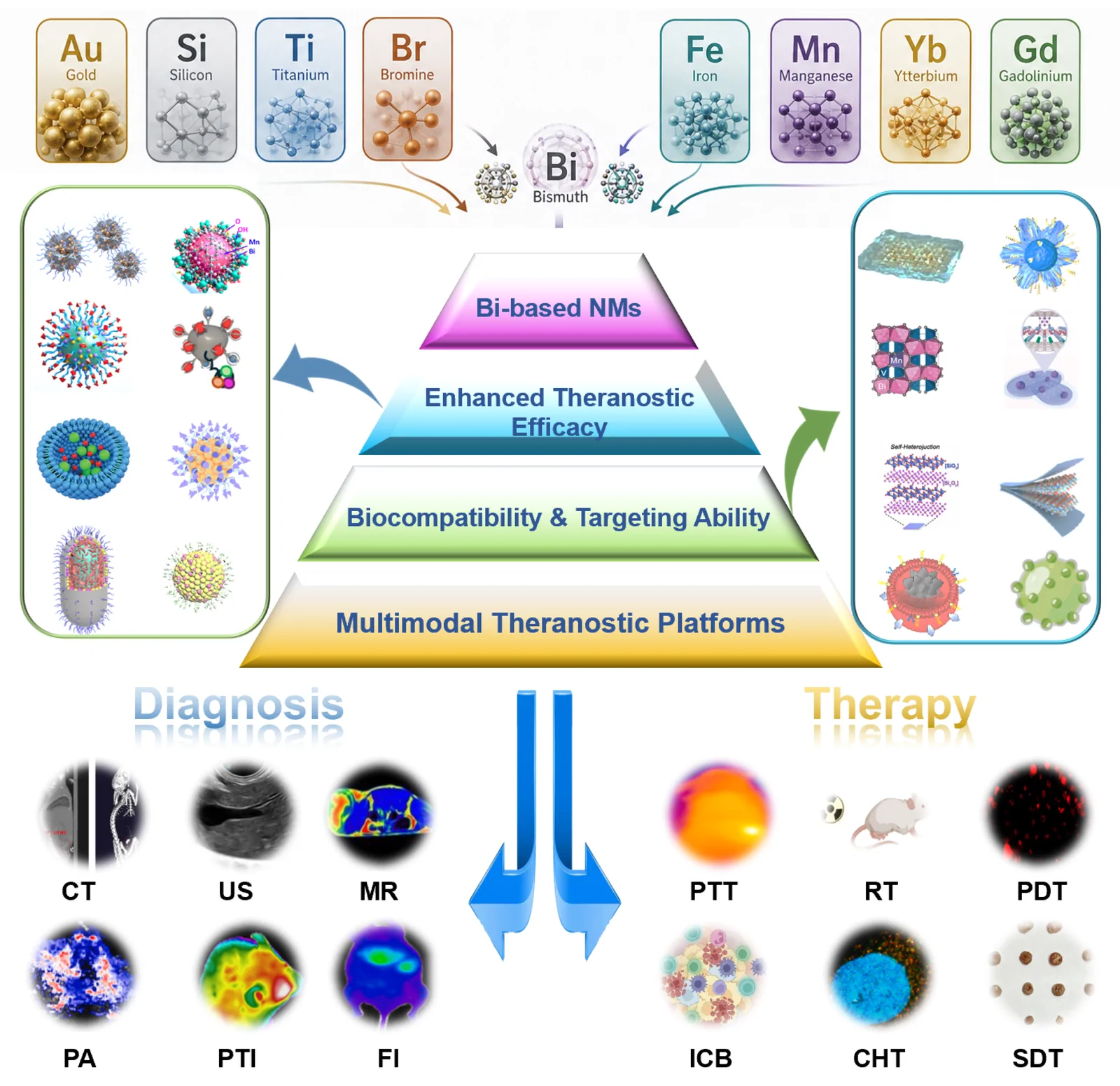
Bi-based nanomaterials possess enormous potential in multimodal imaging and combination therapy. (Created with Microsoft PowerPoint).

**Figure 19:**
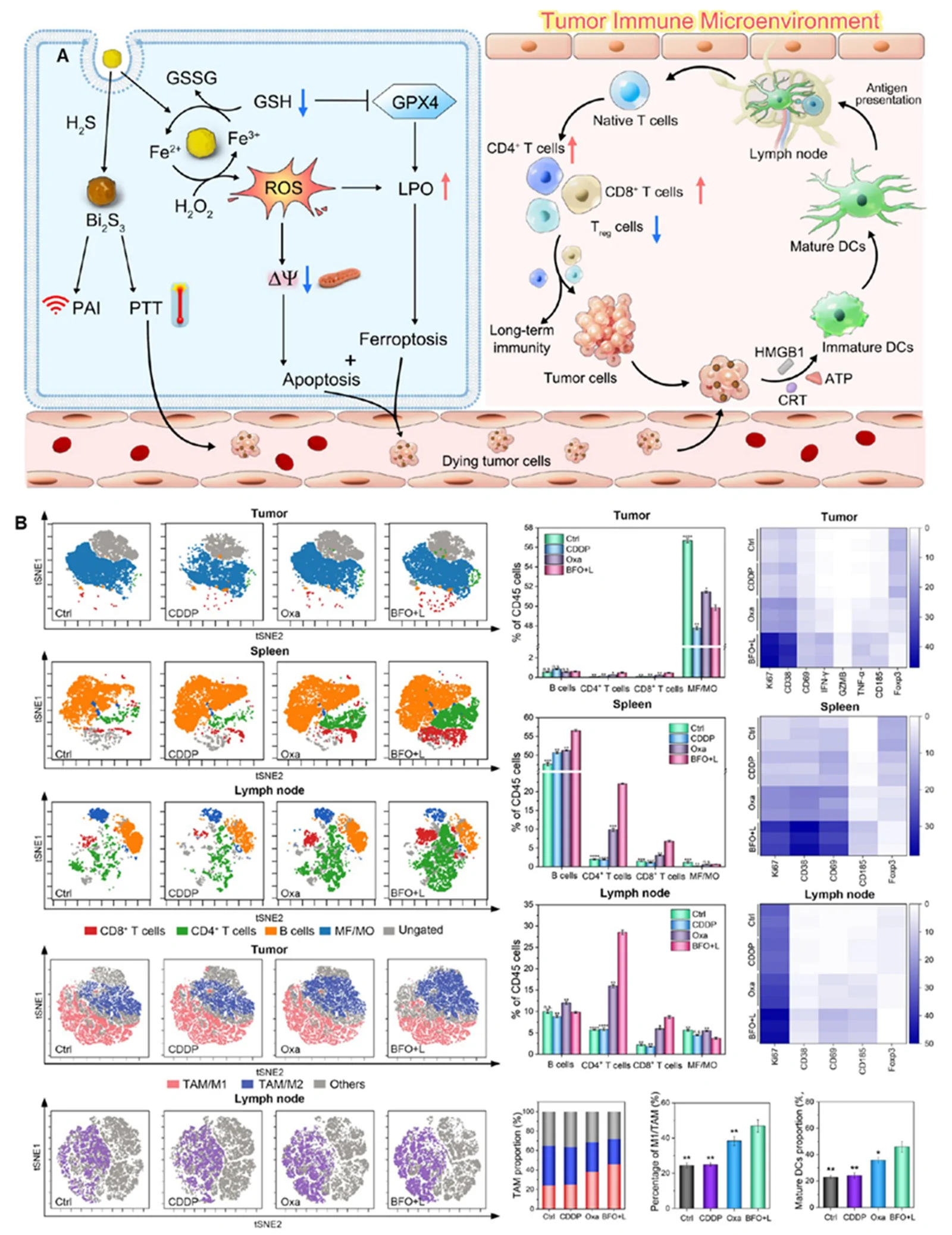
(A) Schematic depiction for the synthesis of H_2_S-activable BiFeO_3-x_ nanoparticles as the theranostic nanoplatform upon 808 nm laser irradiation, triggering the ferroptosis/apoptosis Co-activated ICD. (B) Immune cell analysis in different groups was performed using CyTOF. Adapted with permission from 65, Copyright 2026, American Chemical Society.

**Figure 20:**
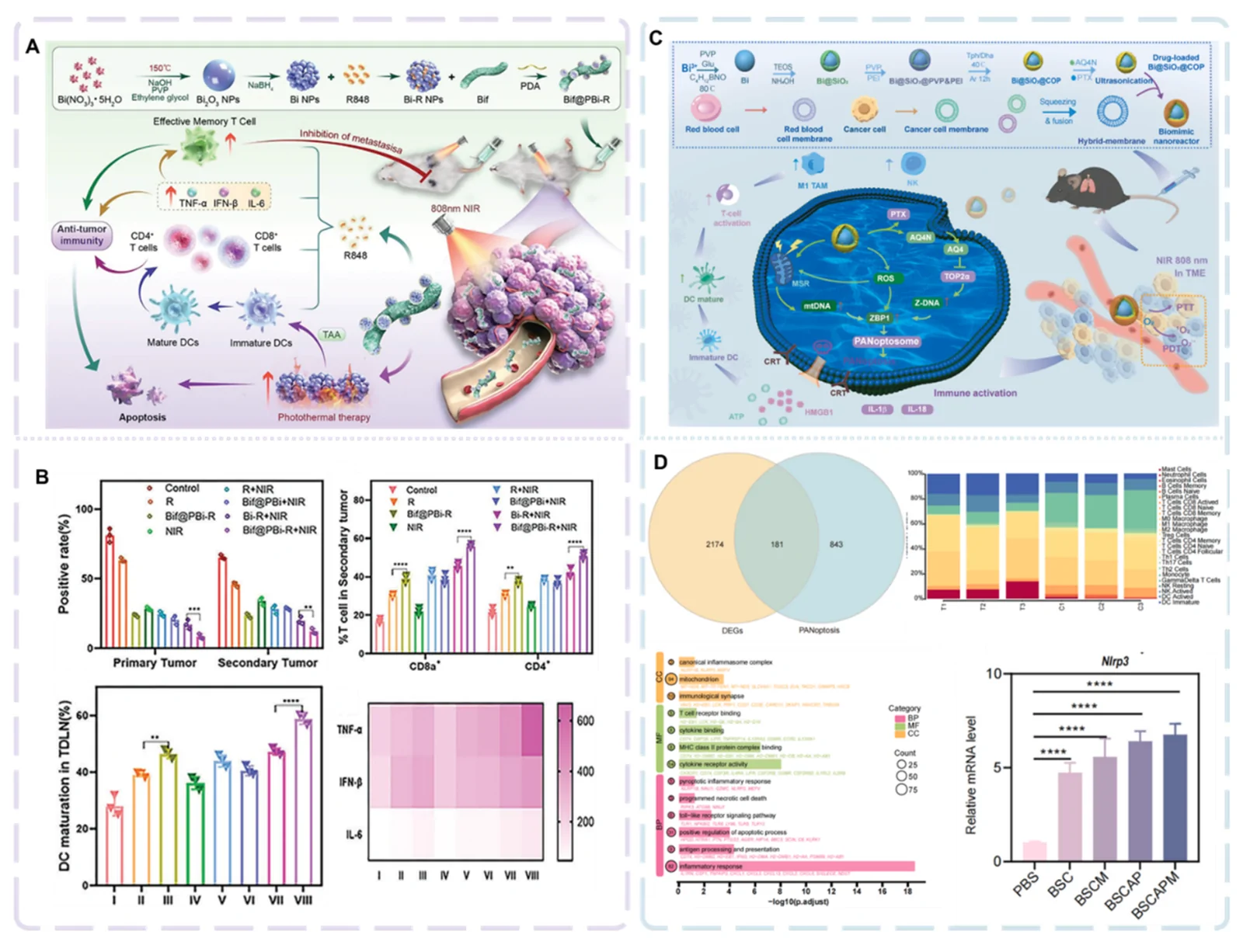
(A) Schematic diagram of the preparation process and anti-tumor mechanism of Bif@PBi-R. (B) Pharmacodynamic evaluation of Bif@PBi-R combined with PTT for remodeling anti-tumor immunity and suppressing tumor proliferation and distant metastasis in bilateral 4T tumor-bearing mouse models. Adapted with permission from 98, Copyright 2024, Wiley-VCH GmbH. (C) The synthetic process of BSCAPM nanoparticles and their anti-tumor mechanisms. (D) Transcriptome combined with qPCR verification that BSCAPM remodels the tumor immune microenvironment via the PANoptosis pathway. Adapted with permission from 111, Copyright 2025, Elsevier Ltd.

**Figure 21:**
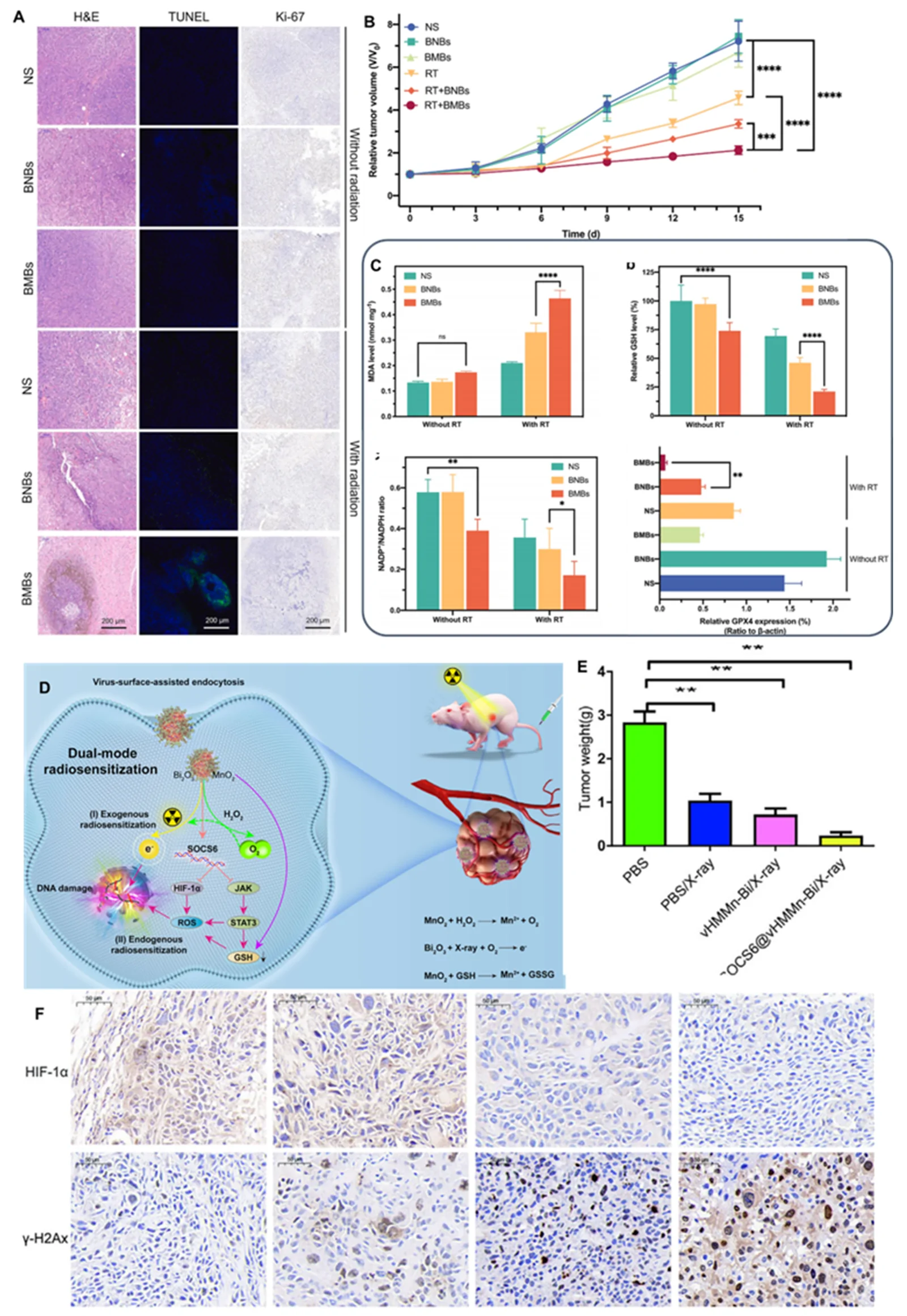
(A) H&E, TUNEL, and antigen Ki-67 analysis of tumor slices subjected to various treatments. (B) Tumor volumes for each group of mice recorded every 3 days after various treatments. (C) *In vivo* mechanism of BMB-triggered ferroptosis for radiotherapy sensitization. Adapted with permission from 103, Copyright 2024, Elsevier B.V. (D) Schematic illustration of virus-inspired SOCS6@vHMMn-Bi nanoparticles with dual-mode endogenous and exogenous sensitization for amplified ESCC radiotherapy. (E) Tumor growth curves of BALB/cmice bearing Kyse-150 tumors after the various treatments. (F) IHC analysis of HIF-1α, γ-H2AX expression in tumor tissues. Adapted with permission from 156, Copyright 2025, Wiley-VCH GmbH.

**Figure 22:**
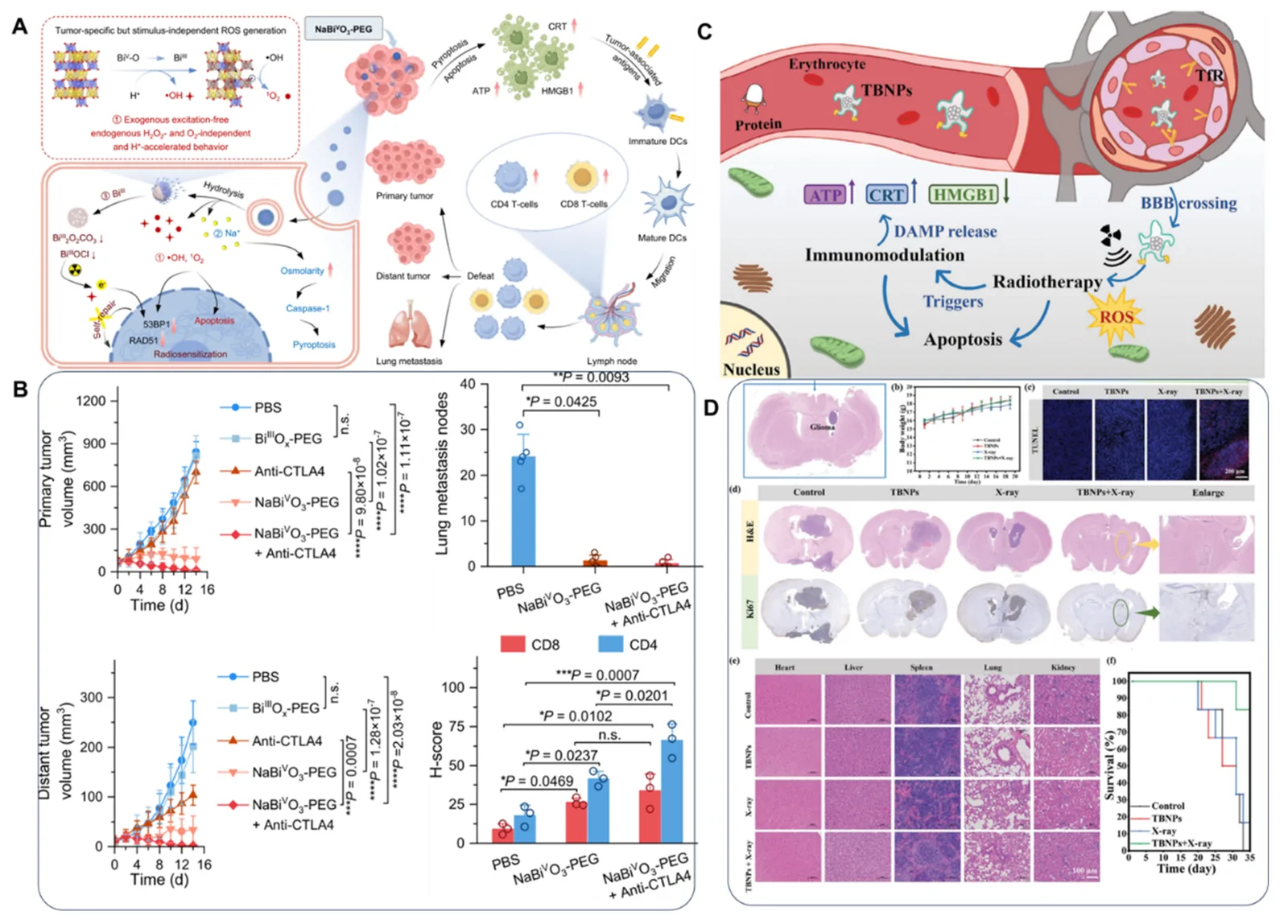
(A) Schematic diagram of exogenous excitation-free and endogenous H_2_O_2_ and O^2-^independent NaBi^Ⅴ^O_3_-PEG for tumor immunotherapy. (B) Anti-tumor and immunotherapeutic performance of NaBiᵛO_3_-PEG *in vivo*. Adapted with permission from 66, Copyright 2025, Springer Nature. (C) Therapeutic mechanism of TBNPs for synergistic radiotherapy and immunotherapy. (D) *In vivo* synergistic radiotherapy-immunotherapy efficacy of TBNPs against orthotopic glioma. Adapted with permission from 88, Copyright 2025, Wiley-VCH GmbH.

**Figure 23:**
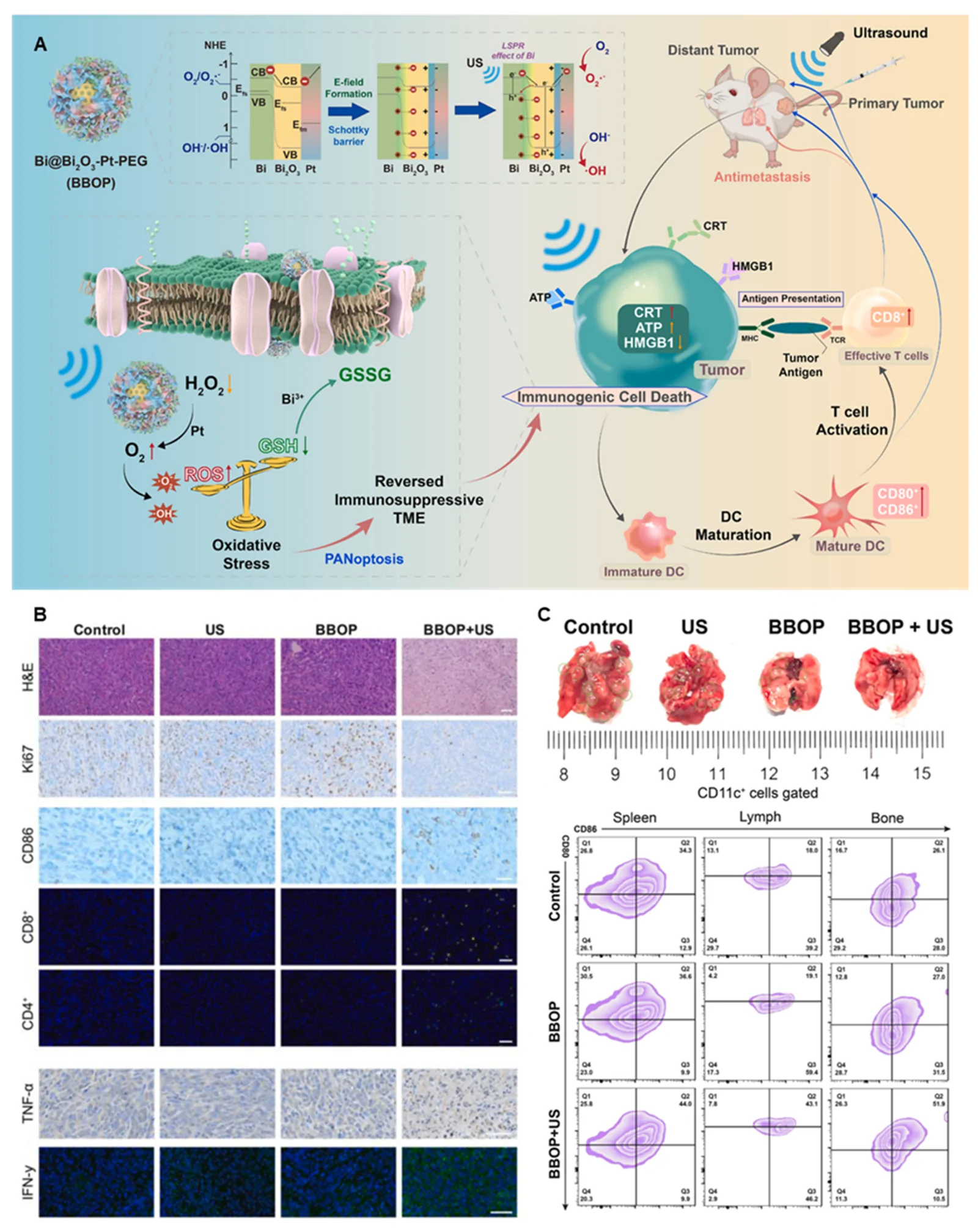
(A) Schematic representation of tumor sono-immunotherapy by Bi-based heteronanoparticles. (B) Changes in tumor proliferation, microenvironment and immune response after sono-immunotherapy. (C) Tumor metastasis inhibition and systemic immune response. Adapted with permission from 114, Copyright 2024, Elsevier Ltd.

**Figure 24:**
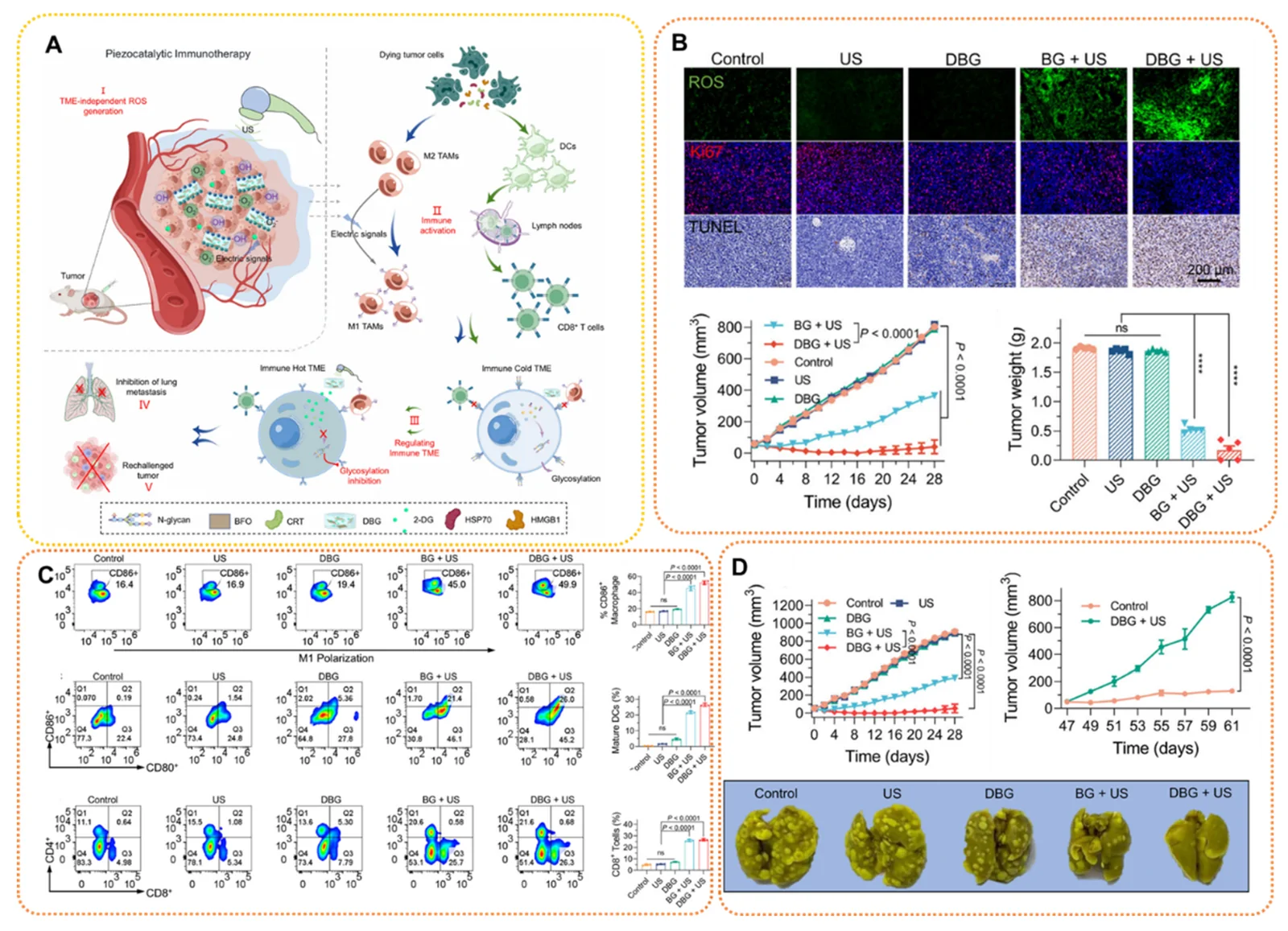
(A) Schematics of amplified tumor piezocatalytic immunotherapy by DBG via *in situ* generating ROS and inhibiting glycosylation of tumor cells. (B) The *in vivo* anticancer performance of DBG under US irradiation. (C) Immune activation evaluations *in vivo*. (D) Effectiveness in combating distant tumors. Adapted with permission from 100, Copyright 2024, Wiley-VCH GmbH.

**Figure 25:**
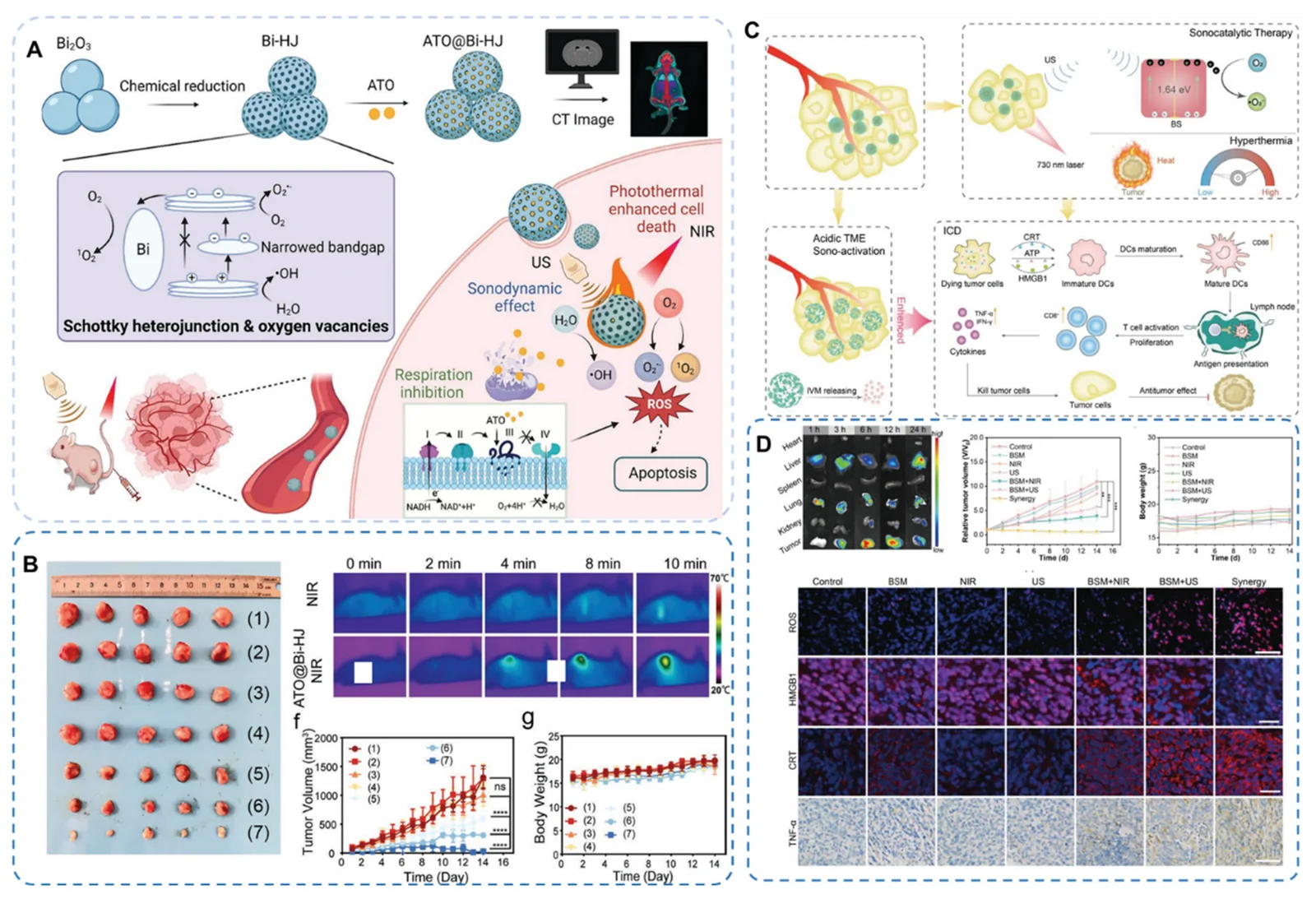
(A) Schematic diagram of the synthesis of Bi-based nanosonosensitizers for metabolic regulation-enhanced sonodynamic/photothermal tumor therapy. (B) *In vivo* combinational therapeutic efficacy. Adapted with permission from 115, Copyright 2023, Wiley-VCH GmbH. (C) Schematic illustration of sono/photo-activated bismuth nanoagonist remodels immune microenvironment for ICD-mediated tumor therapy. (D) *In vivo* anti-tumor effect on breast cancer in BALB/c mice. Adapted with permission from 91, Copyright 2023, Wiley-VCH GmbH.

**Table 1:** Classification, Structural Features and Core Advantages of Bi-Based Nanotheranostic Materials

Material Category	Core Positioning	Prominent Structural Features	Core Advantages	References
Elemental/multimetallic composite Bi-based nanomaterials	Basic Theranostic Materials	Facile synthesis, easily tunable morphology	Strong acoustic response, controllable biocompatibility, intrinsic high X-ray absorption and integrated theranostic potential	64-66
Non-hetero-junction core-shell coated composites	Protective Function-integrated Materials	High stability, easy functional modification	High multifunctional integration, improved colloidal stability, high drug loading capacity, stimuli-responsive release, prolonged *in vivo* circulation time, targeted delivery capability	41, 67, 68
Bi-based typical heterojunction nano-materials	High-performance Catalytic Theranostic Materials	Matched band structure at the interface, efficient separation of photogenerated carriers	Broadened optical response range, enhanced ROS generation, tunable band engineering, integrated functional advantages of multiple materials for efficient synergistic therapy	42, 49, 69
Bi-based/containing metal-organic framework nano-materials	Porous Multifunctional Customizable Materials	High specific surface area, designable pore structure	Programmable pore structure, abundant ligand functionalization sites, excellent performance of derived materials, stimuli-responsiveness, precisely customizable theranostic functions	47, 48, 70

**Table 2:** Summary of elemental/multimetallic composite Bi-based nanomaterials

Category	Representative materials	Structural characteristics	Functional features
Bi-based oxygen-containing compounds	CuBi_2_O_4_, Bi_2_O_2_CO_3_, TBNPs, sulfur-doped bismuth oxides from BiF₃	Layered 2D octahedron with rich oxygen vacancies; topological asymmetric/core-shell structures	TME-degradable; piezoelectric, photothermal, sonodynamic and radiosensitizing effects; glutathione depletion for synergistic therapy
Metallic bismuth	F127-capped Bi nanocrystals, anti-EGFR-MPB, DRBCM-BNF, Bif@PBi-R	Phase-pure metallic lattice; flower-like/porous structure, easy biomodification	Strong X-ray attenuation, excellent NIR photothermal property; TME-controllable degradation; targeting and combinatorial therapy
Bi-based multimetallic composites	Amorphous a-CoBiFe-LDH	Layered LDH matrix; atomic dispersion, interfacial coupling, abundant oxygen vacancies	CT/MRI dual-modal imaging; high US-triggered ROS; enzyme-mimetic oxygen production and immune activation
Bi-based perovskites	KBiO_3_, BiFeO_3_, Bi_2_WO_6_, Bi_4_Ti_3_O_12_, Fe-doped Bi_2_WO_6_	Layered Aurivillius/quasi-perovskite lattice; tunable oxygen vacancies and ferroelectric domains	Piezoelectric/ferroelectric effect accelerates carrier separation; high ROS generation for synergistic theranostics

## Abbreviations

ATO: Atovaquone
ATP: Adenosine Triphosphate
CAF: Cancer-Associated Fibroblast
CRT: Calreticulin
CT: Computed Tomography
DAMPs: Damage-Associated Molecular Patterns
DC: Dendritic Cell
EGFR: Epidermal Growth Factor Receptor
ESCC: Esophageal Squamous Cell Carcinoma
FDA: Food And Drug Administration
GSH: Glutathione
ICD: Immunogenic Cell Death
IVM: Ivermectin
LDH: Layered Double Hydroxides
LSPR: Localized Surface Plasmon Resonance
MOF: Metal-Organic Framework
MRI: Magnetic Resonance Imaging
NIR: Near-Infrared Window
NSCLC: Non-Small Cell Lung Cancer
PA: Photoacoustic
PANoptosis: Pyroptosis, Apoptosis, and Necroptosis
PDT: Photodynamic Therapy
PEG: Polyethylene Glycol
PGA: Polyglutamic Acid
PTT: Photothermal Therapy
PVP: Polyvinylpyrrolidone
ROS: Reactive Oxygen Species
SDT: Sonodynamic Therapy
STING: Stimulator Of Interferon Genes
TAM: Tumor-Associated Macrophages
TME: Tumor Microenvironment
US: Ultrasound

## References

[1] Ravi V M, Will P, Kueckelhaus J, Sun N, Joseph K, Salié H (2022). Spatially resolved multi-omics deciphers bidirectional tumor-host interdependence in glioblastoma. Cancer Cell.

[2] Ring A, Nguyen-Sträuli B D, Wicki A, Aceto N (2023). Biology, vulnerabilities and clinical applications of circulating tumour cells. Nat Rev Cancer.

[3] Cheng H, Su G, Wu Y, Chen G, Yu Z (2023). Extracellular vesicles in anti-tumor drug resistance: Mechanisms and therapeutic prospects. J Pharm Anal.

[4] Holtermann A, Gislon M, Angele M, Subklewe M, Von Bergwelt-Baildon M, Lauber K (2024). Prospects of synergy: Local interventions and CAR-T cell therapy in solid tumors. BioDrugs.

[5] Song S H, Ghosh T, You D G, Joo H, Lee J, Lee J (2023). Functionally masked antibody to uncouple immune-related toxicities in checkpoint blockade cancer therapy. ACS Nano.

[6] Schelker C, Nowak-Sliwinska P, Borchard G (2023). HDACIs and TKIs combinations and their liposomal delivery for cancer treatment. J. Control. Release.

[7] Maitra S, Sarkar S, Chandra M, Ballal S, Kalia R, Arya R (2025). Nanotechnology-enhanced extracellular vesicles-based chipsets in early cancer detection and theranostics. Int. J. Nanomed.

[8] Liu Y, Wang L, Zhang T, Wang C, Fan Y, Wang C (2024). Tumor microenvironment-regulating two-photon probe based on bimetallic post-coordinated MOF facilitating the dual-modal and deep imaging-guided synergistic therapies. ACS Appl. Mater. Interfaces.

[9] Wang C, Ding S, Wang S, Shi Z, Pandey N K, Chudal L (2021). Endogenous tumor microenvironment-responsive multifunctional nanoplatforms for precision cancer theranostics. Coord. Chem. Rev.

[10] Zhan M, Xu Y, Jia L, Yu H, Wang H, Shen M (2024). Biomimetic copper-containing nanogels for imaging-guided tumor chemo-chemodynamic-immunotherapy. Acta Biomater.

[11] Gao J, Yang P, Li H, Li N, Liu X, Cai K (2023). Advances in anti-tumor research based on bionic micro-nano technology. J. Drug Deliv. Sci. Technol.

[12] Zhou Y, Yuan J, Xu K, Li S, Liu Y (2024). Nanotechnology reprogramming metabolism for enhanced tumor immunotherapy. ACS Nano.

[13] Xia M, Li H, Wang Y, Zhang H, Yu Z (2025). An efficient nanoreactor reverses the tumor immunosuppressive microenvironment through synergetic dual-route reactions. Chem. Commun.

[14] Moghaddam F D, Zare E N, Hassanpour M, Bertani F R, Serajian A, Ziaei S F (2024). Chitosan-based nanosystems for cancer diagnosis and therapy: Stimuli-responsive, immune response, and clinical studies. Carbohydr. Polym.

[15] Wang Y, Li S, Ren X, Yu S, Meng X (2023). Nano-engineering nanomedicines with customized functions for tumor treatment applications. J. Nanobiotechnol.

[16] Guo Y, Hu P, Shi J (2024). Nanomedicine remodels tumor microenvironment for solid tumor immunotherapy. J. Am. Chem. Soc.

[17] Wang Y, Yu J, Luo Z, Shi Q, Liu G, Wu F (2021). Engineering endogenous tumor-associated macrophage-targeted biomimetic nano-RBC to reprogram tumor immunosuppressive microenvironment for enhanced chemo-immunotherapy. Adv. Mater.

[18] Xu M, Hu Y, Wu J, Liu J, Pu K (2024). Sonodynamic nano-LYTACs reverse tumor immunosuppressive microenvironment for cancer immunotherapy. J. Am. Chem. Soc.

[19] Shen W, Li Y, Yang Z, Li W, Cao Y, Liu Y (2024). Tumor microenvironment reprogramming combined with immunogenic enhancement by nanoemulsions potentiates immunotherapy. J. Nanobiotechnol.

[20] Yang Z, Gao D, Zhao J, Yang G, Guo M, Wang Y (2023). Thermal immuno-nanomedicine in cancer. Nat. Rev. Clin. Oncol.

[21] Xing T, Yu X, Li W (2026). Bismuth-based nanomaterials with enhanced radiosensitivity for cancer diagnosis and treatment. J. Mater. Chem. B.

[22] Shearer A, Kheilnezhad B, Fan Y, Molinaro M, Montazerian M, Mauro J C (2026). Bismuth-containing bioceramics: Multifunctional platforms for regeneration and therapy. Biophys. Rev.

[23] Zhu H, Li B, Yu Chan C, Low Qian Ling B, Tor J, Yi Oh X (2023). Advances in Single-component inorganic nanostructures for photoacoustic imaging guided photothermal therapy. Adv. Drug Deliv. Rev.

[24] Shu G, Zhang C, Wen Y, Pan J, Zhang X, Sun S-K (2024). Bismuth drug-inspired ultra-small dextran coated bismuth oxide nanoparticles for targeted computed tomography imaging of inflammatory bowel disease. Biomaterials.

[25] Shi S, Li X, Zhang Y, Huang H, Liu J, Zhang J (2024). Ultrathin and biodegradable bismuth oxycarbonate nanosheets with massive oxygen vacancies for highly efficient tumor therapy. Small.

[26] Deng J, Zhang Y, Xiang K, Shu G, Wang J, Pan J (2026). Synthesis and preclinical evaluation of a bismuth pyclen chelate with superior water solubility, high stability, and low osmolarity for multisystem CT imaging in vivo. Biomaterials.

[27] Huang X, Zha F, Zou J, Li Y, Wang F, Chen X (2022). Photoacoustic imaging-guided synergistic photothermal/radiotherapy using plasmonic Bi/Bi2O3-x nanoparticles. Adv. Funct. Mater.

[28] Wu Y, Zeng F, Zhao Y, Wu S (2021). Emerging contrast agents for multispectral optoacoustic imaging and their biomedical applications. Chem. Soc. Rev.

[29] Lai Y, Bai M, Huang A, Chiang T, Wang Y, Wu C (2024). Near-infrared radiation of gold-platinum submicron particle-decorated nonwoven mats enhances wound healing: In vitro and In vivo studies. Mater. Today Commun.

[30] Bai L, Yi W, Chen J, Wang B, Tian Y, Zhang P (2022). Two-stage targeted bismuthene-based composite nanosystem for multimodal imaging guided enhanced hyperthermia and inhibition of tumor recurrence. ACS Appl. Mater. Interfaces.

[31] Kou X, Yue J, Cong Y, Gao R, Dong W, Li L (2026). Boron-doped AIE nanomaterials with NIR-II emission for FL/MR dual-modality tumor imaging. Small.

[32] Wang C, Hu J, Huang Y, Chen Y, Zhan L, Liu H (2026). FAP-targeting peptide-directed nanoprobes enable tumor microenvironment-activatable MR/NIRF imaging of breast cancer primary tumor and lung metastases. Mater. Today Bio.

[33] Liu S, Li H, Ding Y, Duan D, Cao L, Liu K (2026). MRI-based quantitative evaluation of tumor ferroptosis mediated by a novel self-reducing nano-prodrug. Adv. Healthcare Mater.

[34] Jung W, Son Y, Lee D Y, Jeon Y, Asaddudin M, Park S (2026). ROS-responsive nanobubbles for dual-enhanced ultrasound and magnetic resonance imaging of tumor oxidative stress. Adv. Sci.

[35] Wu S, Li W, Yang Q, Song L, Huang W, Chen Z (2026). Synchronous PET/MR imaging and visualizing penetration of nanomedicine within tumor based on ⁶⁸Ga-NaGdF₄ probes. J. Nanobiotechnol.

[36] Zhao J, Zhang H, Lu J, Fan M, Jie P, Yuan Z (2026). Tumor microenvironment-programmed dual-responsive nanoprobes enable fluorescence/photoacoustic imaging-guided enhanced tumor phototherapy by blocking protective autophagy. Chem. Eng. J.

[37] Xiao F, Hua S, Liu F, Yan Z, Yi Z, Xu Y (2026). A bismuth-based metal-phenolic nanocoordinator blocks TGF β to reverse immunosuppression for potentiated hepatocellular carcinoma radioimmunotherapy. Adv. Funct. Mater.

[38] He Z, Shao X, Feng X, Yin L, Yang N, Wang Y (2026). Photothermal sensitive nano heterojunction triggering ferroptosis and PANoptosis for improving tumor immunotherapy. Adv. Mater.

[39] Wang J, Hou W, Qiu X, Zhang Y, Tao L, Chen Y (2026). Ursolic acid-loaded iron-MOF nanomedicine inhibits lung cancer metastasis via JAK2/STAT3 suppression and ferroptosis. Adv. Healthcare Mater.

[40] Mishra A, San Valentin E M D, Barcena A J R, Bolinas D K M, Bernardino M R, Canlas G (2025). Antibody-targeted bismuth gadolinium nanoconjugate for image-guided radiotherapy of hepatocellular carcinoma. ACS Appl. Mater. Interfaces.

[41] Chen Z, Lin H, Yoon C, Huang H, Kim Y, Meng C (2026). Bismuthene-based nanoplatform for synergistic thermogenetic CRISPR and photothermal cancer therapy. Nano Lett.

[42] Pang Y, Guo J, Ma Q, Qi J, Liu L, Bu Y (2025). Hyaluronic acid-functionalized bismuth vanadate/molybdenum disulfide nanoheterojunctions achieve efficient phototherapy of hypoxic tumor. Biomater. Res.

[43] Wang X, Cui Z, Jia Q, Hao C, Wu B, Wang B (2025). Injectable bismuth-based composite enable bone defect repair for osteosarcoma treatment and mild magnetothermal bone regeneration. Adv. Funct. Mater.

[44] Zhu L, Wang Q, Du J, Li X, Meng Q, Lu J (2025). Non-central symmetric 2D bismuth-based perovskites for piezoelectric-enhanced sonodynamic immunotherapy. J. Colloid Interface Sci.

[45] Zhang X, Gao F, Dai Y, Wang M, Liu J (2025). Safe-by-design strategies towards bismuth-based nanomaterials in tumor diagnosis and therapy. Nano Today.

[46] Chaki Borrás M L, Barker P J, Sluyter R, Konstantinov K (2025). Theranostic nonstoichiometric, multivalent bismuth (Bi)-based nanospheres for selective spontaneous melanoma treatment. ACS Appl. Mater. Interfaces.

[47] Wu X, Zhang J, Deng Z, Sun X, Zhang Y, Zhang C (2024). Bacteria-based biohybrids for remodeling adenosine-mediated immunosuppression to boost radiotherapy-triggered antitumor immune response. Biomaterials.

[48] Gu L, Li X, Chen G, Yang H, Qian H, Pan J (2024). A glutathione-activated bismuth-gallic acid metal-organic framework nano-prodrug for enhanced sonodynamic therapy of breast tumor. J. Colloid Interface Sci.

[49] Zhu L, Chen G, Wang Q, Du J, Wu S, Lu J (2024). High-Z elements dominated bismuth-based heterojunction nano-semiconductor for radiotherapy-enhanced sonodynamic breast cancer therapy. J. Colloid Interface Sci.

[50] Hao P, Yang L, Yan Y, Wang X, Yin J, Hong W (2024). Metal-based nanocomposites for immunotherapy of osteosarcoma. Adv. Compos. Hybrid Mater.

[51] Li Y, Han Y, Li H, Niu X, Liu X, Zhang D (2023). Study of bismuth metal organic skeleton composites with photocatalytic antibacterial activity. J. Colloid Interface Sci.

[52] Zhao Y, Wang X, He M, Zeng G, Xu Z, Zhang L (2023). Vacancy-rich bismuth-based nanosheets for mitochondrial destruction via CO Poisoning, Ca2⁺ dyshomeostasis, and oxidative damage. Small.

[53] Zhang Q, Tu J, Jiang Y, Wang H, Xue W, Dong C (2025). Bismuth-based nanomaterials: Design, synthesis and applications in tumor synergistic therapy. Colloids Surf, B.

[54] Charles I A, Nnenna H U, Pelumi A, Rukayat O B, Abisoye O F, Ruth J A (2025). Radiotherapy-chemodynamic cancer therapy using bismuth-based nanoparticles: A synergistic approach for enhanced cancer treatment. RSC Adv.

[55] Yang Z, Wang L, Zhang J, Liu J, Yu X (2023). Application of bismuth sulfide-based nanomaterials in cancer diagnosis and treatment. Nano Today.

[56] Hong C, Chen T, Wu M, Lin J, Gao C, Ma X (2023). Bismuth-based two-dimensional nanomaterials for cancer diagnosis and treatment. J. Mater. Chem. B.

[57] Dhanraj G, Chitra S, Rajeshkumar S, Ashok K (2022). S, Karthikeyan M, Sivaperumal P, et al. Recent breakthrough of bismuth-based nanostructured materials for multimodal. J. Nanomater.

[58] Griffith D M, Li H, Werrett M V, Andrews P C, Sun H (2021). Medicinal chemistry and biomedical applications of bismuth based compounds and nanoparticles. Chem. Soc. Rev.

[59] Zheng L, Zhu R, Chen L, Fu Q, Li J, Chen C (2021). X-ray sensitive high-Z metal nanocrystals for cancer imaging and therapy. Nano Res.

[60] Ali T, Masoud A, Leili D, Azadeh A, Morteza G, Ayuob A (2026). Radiosensitivity enhancement of bismuth-based nanoparticles in radiotherapy: A systematic review and meta-analysis. Comput. Biol. Chem.

[61] Daryoush S G, Yazdan C, Arezoo K, Paria N, Saghar S G (2023). A Review of bismuth-based nanoparticles and their applications in radiosensitising and dose enhancement for cancer radiation therapy. IET Nanobiotechnol.

[62] Wen S, Ovais M, Li X, Ren J, Liu T, Wang Z (2022). Tailoring bismuth-based nanoparticles for enhanced radiosensitivity in cancer therapy. Nanoscale.

[63] Amna B, Ina K, Manja K, Michael B, Philip C A, Holger S (2025). Targeted bismuth-based materials for cancer. Dalton Trans.

[64] He Z, Du J, Wang Q, Chen G, Li X, Zhang Z (2024). Dye-augmented bandgap engineering of a degradable cascade nanoreactor for tumor immune microenvironment-enhanced dynamic phototherapy of breast cancer. Acta Biomater.

[65] Liang J, Luo X, Shi Y, Yang L, Li C, Huang L (2026). H2S-activable BiFeO3-x nanocatalysts for ferroptosis-driven cancer immunotherapy. ACS Nano.

[66] Tang Y, Yu X, He L, Tang M, Yue W, Chen R (2025). A high-valence bismuth(V) nanoplatform triggers cancer cell death and anti-tumor immune responses with exogenous excitation-free endogenous H2O2 and O2-independent ROS generation. Nat. Commun.

[67] Jia P, Tu J, Shen H, Jiang Y, Zhang Q, Xue W (2025). Defect-engineered magnetic bismuth nanomedicine for dual-modal imaging and synergistic lung tumor therapy. Mater. Today Bio.

[68] Song X, Xiao L, Xu L, Jiang Y, Fu W, Chen B (2025). Fe₃O₄@Bi₂S₃ nanoparticles mediated MRI-guided precision radiosensitization for orthotopic glioblastoma via external magnetism. ACS Appl. Mater. Interfaces.

[69] Wang Q, Du J, Yang F, Wu S, Zhu L, Li X (2024). Charge separation-engineered piezoelectric ultrathin nanorods modulate tumor stromal microenvironment and enhance cell immunogenicity for synergistically piezo-thermal-immune therapy. Small.

[70] Chang Y, Huang K, Tang H, Yao Y, Min J, Quan H (2025). Biomimetic MOF nanoplatform for dual-targeted co-delivery of FAK inhibitor and bismuth to enhance cervical cancer radiosensitivity. Adv. Compos. Hybrid Mater.

[71] Zavestovskaya I N, Filimonova M V, Popov A L, Zelepukin I V, Shemyakov A E, Tikhonowski G V (2024). Bismuth nanoparticles-enhanced proton therapy: Concept and biological assessment. Mater. Today Nano.

[72] Cao Q, Yang C, Yao Y, Li B, Liu J, Cao Z (2023). Learning from human metabolism for nanomedicine: a convertible bismuth-agent for tumour-selective theranostics. Mater. Horiz.

[73] Wang J, Zheng H, Hu G, Yang X, You H, Dong L (2024). Novel spatially asymmetric copper bismuthate-mediated augmentation of energy conversion to realize “three-step” tumor suppression. Adv. Sci.

[74] Song K, Chen G, He Z, Shen J, Ping J, Li Y (2023). Protoporphyrin-sensitized degradable bismuth nanoformulations for enhanced sonodynamic oncotherapy. Acta Biomater.

[75] Huang H, Du J, Meng Q, Pu S, Chen S, Liu K (2026). Lattice distortion-mediated synergistic piezoelectric-long-acting chemodynamic tumor therapy via ferroptosis with cation-doped bismuth nanospheres. ACS Appl. Mater. Interfaces.

[76] Liu J, Liu P, Duan J, Xie Q, Feng J, Tan H (2024). Macrophages-mediated tumor accumulation and deep penetration of bismuth/manganese biomineralized nanoparticles for enhanced radiotherapy. Chin. Chem. Lett.

[77] Peng R, Ainiwa G, Luo Y, Zhang K, Zhang R, Duan B (2025). Cascade-amplified oxidative stress via bandgap-tuned KBiO₃ perovskite for cancer therapy. Small.

[78] Mao W, Shen X, Zhang L, Liu Y, Liu Z, Guan Y (2024). A review on Bi2WO6-based photocatalysts synthesis, modification, and applications in environmental remediation, life medical, and clean energy. Front. Environ. Sci. Eng.

[79] Han J, Chen M, Hao S, Li N, Wen F, He K (2025). Bi2WO6@PDA nanosheet with near-infrared induced emission for combinational photothermal/photodynamic therapy. Adv. Mater. Technol.

[80] Lao X, Bai Q, Zhao Y, Dai X, Liu Y, Han X (2025). Enhanced sonodynamic bacterial elimination and wound healing therapy based on lanthanide ion doped Bi₂WO₆ Nanosheets and Hydrogel Platform. Adv. Funct. Mater.

[81] Wu M, Yong J, Zhang H, Wang Z, Xu Z P, Zhang R (2023). 2D ultrathin iron doped bismuth oxychloride nanosheets with rich oxygen vacancies for enhanced sonodynamic therapy. Adv. Healthcare Mater.

[82] Zhang X, Guo R, Chen Y, Xu X, Yang Z, Cheng D (2021). A BiOCl nanodevice for pancreatic tumor imaging and mitochondria-targeted therapy. Nano Today.

[83] Yu C, Dong Y, Zhu X, Feng L, Zang P, Liu B (2024). Oxygen vacancy piezoelectric nanosheets constructed by a photoetching strategy for ultrasound “unlocked” tumor synergistic therapy. Nano Lett.

[84] Yang L, Tian B, Xie Y, Dong S, Yang M, Gai S (2023). Oxygen-vacancy-rich piezoelectric BiO2-x nanosheets for augmented piezocatalytic, sonothermal, and enzymatic therapies. Adv. Mater.

[85] Liao Y, Wang D, Zhu S, Zhou R, Rahbarizadeh F, Gu Z (2022). Piezoelectric materials for synergistic piezo- and radio-catalytic tumor therapy. Nano Today.

[86] Du Y, Yang J, He F, Zhao X, Zhou J, Zang P (2023). Revealing the mutually enhanced mechanism of necroptosis and immunotherapy induced by defect engineering and piezoelectric effect. Adv. Mater.

[87] Ma J, Yuan M, Yang Z, Ma Z, Zhang J, Li Z (2024). Surface oxygen vacancies and corona polarization of Bi₄Ti₃O₁₂ nanosheets for synergistically enhanced sonopiezoelectric therapy. J. Am. Chem. Soc.

[88] Huang X, Ge W, Li S, Huang R, Wang F (2025). Transferrin-based bismuth nanoparticles for radiotherapy with immunomodulation against orthotopic glioma. Adv. Healthcare Mater.

[89] Du J, He Z, Wang Q, Chen G, Li X, Lu Ji (2024). Topochemical-like bandgap regulation engineering: A bismuth thiooxide nanocatalyst for breast cancer phototherapy. J. Colloid Interface Sci.

[90] Chen G, Gu L, Liu Y, Du J, Qi Q, Miao Q (2023). Topology-regulated nanocatalysts for ferroptosis-mediated cancer phototherapy. J. Colloid Interface Sci.

[91] Chen G, Yang Z, Du J, He Z, Zhang Y, Zheng K (2023). Topological regulating bismuth nano-semiconductor for immunogenic cell death-mediated sonocatalytic hyperthermia therapy. Small.

[92] Bogusz K, Tehei M, Cardillo D, Lerch M, Rosenfeld A, Dou S X (2018). High toxicity of Bi(OH)3 and α-Bi2O3 nanoparticles towards malignant 9L and MCF-7 cells. Mater. Sci. Eng, C.

[93] Bogusz K, Tehei M, Stewart C, Mcdonald M, Cardillo D, Lerch M (2014). Synthesis of potential theranostic system consisting of methotrexate-immobilized (3-aminopropyl) trimethoxysilane coated α- Bi2O3 nanoparticles for cancer treatment. RSC Adv.

[94] Stewart C, Konstantinov K, Mckinnon S, Guatelli S, Lerch M, Rosenfeld A (2016). First proof of bismuth oxide nanoparticles as efficient radiosensitisers on highly radioresistant cancer cells. Phys. Med.

[95] Chaki Borrás M L, Colbran G, Mitchell D R G, Barker P J, Sluyter R, Konstantinov K (2023). Multifunctional bismuth oxide (Bi2O3) particles: Evidence for selective melanoma therapy. J. Biomed. Mater. Res, Part A.

[96] Mondal S, Park S, Nguyen V T, Doan V H M, Choi J, Ly C D (2024). Precision cancer therapy enabled anti-epidermal growth factor receptor-conjugated manganese core phthalocyanine bismuth nanocomposite for dual imaging-guided breast cancer treatment. Biomater. Res.

[97] Wen J, Liu C, Liu J, Wang L, Miao S, Chen D (2023). Dextran 40 hybrid biomimetic bismuth-nanoflower designed for NIR II-triggered hypoxic tumor thermoradiotherapy via macrophage escape. Carbohydr. Polym.

[98] Xiao S, Feng C, Mu M, Yu W, Wang X, Li H (2024). Bacteria-mediated bismuth-based nanoparticles activate toll-like receptors for breast cancer photothermal immunotherapy. Adv. Funct. Mater.

[99] Cao M, Yang S, Li J, Yang Y, Zhan L, Wang T (2024). Bifunctional bismuth-based layered double hydroxide sonosensitizer for magnetic resonance imaging-guided sonodynamic cancer therapy. Small.

[100] Pu Y, Zhou B, Bing J, Wang L, Chen M, Shen Y (2024). Ultrasound-triggered and glycosylation inhibition-enhanced tumor piezocatalytic immunotherapy. Nat. Commun.

[101] Ding Y, Zhao Y, Yao S, Wang S, Wan X, Hu Q (2023). Enhanced sonodynamic cancer therapy through iron-doping and oxygen-vacancy engineering of piezoelectric bismuth tungstate nanosheets. Small.

[102] Qian H, Pan J, Li X, Du J, Gu L, Miao Y (2025). Anion exchange regulated charge separation engineering in bismuth nanoflowers for sonocatalytic radio-immunotherapy. J. Colloid Interface Sci.

[103] Yin Y, Zhu L, Jiang T, Chai R, Zhang Y, Li T (2024). Engineered multienzyme-mimicking 2D bismuthene catalytic nanotriggers enable cascade enzyodynamic-boosted and synergistic GPX4/FSP1-mediated ferroptosis amplification for cancer radiosensitization. Chem. Eng. J.

[104] Wu S, Wang Q, Du J, Zhu L, Yang F, Lu J (2024). Bi-Pt heterojunction cascade reaction platform for sono-immunotherapy of tumors via PANoptosis and ferroptosis. Adv. Healthcare Mater.

[105] Li G, Dai X, Liu Y, Chen J, Yu W, Li P (2025). Alternating interlayered piezoelectric self-heterojunction boosts sono-piezocatalytic pyroptosis oncotherapy. Adv. Mater.

[106] Yang W, Di S, Yang Z, Cao J, Fu Q, Ren H (2025). One-dimensional nanosonosensitizer boosted multiple branches of immune responses against MHC-deficient immune-evasive urologic tumor. Sci. Adv.

[107] Lv J, Wang X, Zhang X, Xu R, Hu S, Wang S (2023). Tumor microenvironment-responsive artesunate loaded Z-scheme heterostructures for synergistic photo-chemodynamic therapy of hypoxic tumor. Asian J. Pharm. Sci.

[108] Xiao C, Wang R, Fu R, Yu P, Guo J, Li G (2024). Piezo-enhanced near infrared photocatalytic nanoheterojunction integrated injectable biopolymer hydrogel for anti-osteosarcoma and osteogenesis combination therapy. Bioact. Mater.

[109] Pan J, Qian H, Zheng J, Li X, Wang B, Du J (2025). Ultrasound-enhanced dual-responsive bismuth nanocatalysts for alleviating tumor hypoxia and promoting breast cancer sonodynamic-immunotherapy. Chem. Eng. J.

[110] Li L, Lin Z, Xu X, Wang W, Chen H, Feng Z (2023). A pH/GSH/glucose responsive nanozyme for tumor cascade amplified starvation and chemodynamic theranostics. ACS Appl. Mater. Interfaces.

[111] Xu D, Xu T, Zhang Y, Su Y, Hou X, Cui Y (2025). Nanoreactor-enabled PANoptosis via ZBP1 activation potentiates immunotherapy in non-small cell lung cancer. Biomaterials.

[112] Hu T, Lin S, Yang Z, Li Y, Yang C, Xu M (2025). Bismuth-based dissolvable microneedle: A precision-targeted “surgical scalpel” for dual-gas synergistic tumor therapy. J. Colloid Interface Sci.

[113] Zhang L, Fu J, Song L, Cheng K, Zhang F, Tan W (2023). Ultrasmall Bi/Cu coordination polymer combined with glucose oxidase for tumor enhanced chemodynamic therapy by starvation and photothermal treatment. Adv. Healthcare Mater.

[114] He Z, Wang Q, Du J, Wu S, Miao Q, Li Y (2024). Overcoming tumor hypoxic bismuth-based ternary heterojunctions enable defect modulation-augmented tumor sonocatalytic immunotherapy. Biomaterials.

[115] Cao X, Wang Y, Song X, Lou W, Li X, Lu W (2023). Defect-engineering bismuth-based homologous schottky heterojunction for metabolic regulation-augmented sonodynamic tumor therapy. Adv. Funct. Mater.

[116] Wang Q, Liu J, Chen D, Miao S, Wen J, Liu C (2023). “Cluster Bomb” based bismuth nano-in-micro spheres formed dry powder inhalation for thermo-radio sensitization effects of lung metastatic breast cancer. Adv. Healthcare Mater.

[117] Wu B, Lu S, Yu H, Liao R, Li H, Lucie Zafitatsimo B V (2018). Gadolinium-chelate functionalized bismuth nanotheranostic agent for in vivo MRI/CT/PAI imaging-guided photothermal cancer therapy. Biomaterials.

[118] Chen J, Ran P, Xu Y, Khouchani M, Li X, Jian L (2025). Biomimetic multifunctional nanoparticles for improved radiotherapy and immunotherapy in cancer treatment. Mater. Today Bio.

[119] Ding M, Liu J, Yang J, Wang H, Xie X, Yang X (2022). How do bismuth-based nanomaterials function as promising theranostic agents for the tumor diagnosis and therapy?. Curr. Med. Chem.

[120] Molaei M J (2023). Turmeric-derived gadolinium-doped carbon quantum dots for multifunctional fluorescence imaging and MRI contrast agent. J. Lumin.

[121] Dhamija P, Mehata A K, Setia A, Priya V, Malik A K, Bonlawar J (2023). Nanotheranostics: Molecular diagnostics and nanotherapeutic evaluation by photoacoustic/ultrasound imaging in small animals. Mol. Pharm.

[122] Gomez C, Hallot G, Laurent S, Port M (2021). Medical applications of metallic bismuth nanoparticles. Pharmaceutics.

[123] Wang J, Wang Y, Zhong L, Yan F, Zheng H (2024). Nanoscale contrast agents: A promising tool for ultrasound imaging and therapy. Adv. Drug Deliv. Rev.

[124] Huang H, Zheng Y, Chang M, Song J, Xia L, Wu C (2024). Ultrasound-based micro-/nanosystems for biomedical applications. Chem. Rev.

[125] Meng R, Ye Y, Xia H, Wang S, Chen A, Kankala R K (2025). Nanoarchitectured bismuth-based structures for diverse applications: Recent advancements and latest breakthroughs. Coord. Chem. Rev.

[126] Jin L, Zhu S, Wang H, Tai X, Wang X (2026). Bismuth-based oxides in piezocatalysis: Structure, mechanism, and multifunctional applications. Nano Energy.

[127] Shetty A, Chandra S (2020). Inorganic hybrid nanoparticles in cancer theranostics: understanding their combinations for better clinical translation. Mater. Today Chem.

[128] Bauri S, Tripathi S, Choudhury A M, Mandal S S, Raj H, Maiti P (2023). Nanomaterials as theranostic agents for cancer therapy. ACS Appl. Nano Mater.

[129] Liu J, Li Q, Tu H, Feng J, Fan X, Mi Z (2024). Bismuth/manganese-doped carbon dots in calcium phosphate matrix: Dynamic conformational nanoparticles enhancing tumor accumulation, deep penetration, and radiosensitivity. Chem. Eng. J.

[130] Meng X, Liu J, Zheng Q, Li S, Xiao H, Huang J (2023). Gold-crowned bismuth-based nanocomposites for sonodynamic, photothermal, and chemotherapeutic cancer therapy. ACS Appl. Mater. Interfaces.

[131] Muradova Z, Carmès L, Brown N, Rossetti F, Guthier R, Yasmin-Karim S (2025). Targeted-theranostic nanoparticles induce anti-tumor immune response in lung cancer. J. Nanobiotechnol.

[132] Li S, Hou H, Chu X, Zhu Y, Zhang Y, Duan M (2024). Nanomaterials-involved tumor-associated macrophages’ reprogramming for antitumor therapy. ACS Nano.

[133] Hao J, Zhao X, Wang C, Cao X, Liu Y (2024). Recent advances in nanoimmunotherapy by modulating tumor-associated macrophages for cancer therapy. Bioconjugate Chem.

[134] Zhang Q, Tu J, Jiang Y, Wang H, Xue W, Dong C (2025). Bismuth based nanomaterials: Design, synthesis and applications in tumor synergistic therapy. Colloids Surf, B.

[135] Khan M, Sun X, Kashif M, Zada A, Azizi S, Ragab A H (2025). Exploration of bismuth-based nanomaterials: From fundamental concepts to innovative synthesis techniques and emerging applications. Coord. Chem. Rev.

[136] Xu B, Cui Y, Wang W, Li S, Lyu C, Wang S (2020). Immunomodulation-enhanced nanozyme-based tumor catalytic therapy. Adv. Mater.

[137] Lin H, Li Z, Zelepukin I V, Deyev S M, Yang X, Li Z (2025). Effects of nanoparticle physicochemical properties on macrophage polarization. J. Control. Release.

[138] Xie D, Wang Q, Wu G (2022). Research progress in inducing immunogenic cell death of tumor cells. Front. Immunol.

[139] Tsai K, Chen C, Su J, Lee Y, Tseng Y, Wei W (2023). The blockade of mitogen-activated protein kinase 14 activation by marine natural product crassolide triggers ICD in tumor cells and stimulates anti-tumor immunity. Mar. Drugs.

[140] Peng T, Xu T, Liu X (2022). Research progress of the engagement of inorganic nanomaterials in cancer immunotherapy. Drug Deliv.

[141] Pan Y, Cheng J, Zhu Y, Zhang J, Fan W, Chen X (2024). Immunological nanomaterials to combat cancer metastasis. Chem. Soc. Rev.

[142] Xie M, Sun W, Hu D, Liu L, Han C, Liu W (2026). From barrier to gateway: Nanomaterials reshaping the tumor microenvironment for therapy. Int. J. Nanomed.

[143] Zhang R, Chen G, Du J, Wang Q, Qi Q, Li X (2023). Rare earth regulatory defect engineering: A multifunctional nanoplatform for breast cancer therapy through PANoptosis. Chem. Eng. J.

[144] Chen J, Cheng X (2025). Nanomaterials and immune checkpoint inhibitors in cancer immunotherapy: the synergistic innovation prospects. Front. Immunol.

[145] Tan M, Cao G, Wang R, Cheng L, Huang W, Yin Y (2024). Metal-ion-chelating phenylalanine nanostructures reverse immune dysfunction and sensitize breast tumour to immune checkpoint blockade. Nat. Nanotechnol.

[146] Sun Z, Wang N, Wu Y, Wen S, Jin D (2025). Recent advances in nanomaterials for integrated phototherapy and immunotherapy. Coord. Chem. Rev.

[147] Jiang X, Huang Z, Liu Z, Wang S, Qiu Y, Su X (2024). MOF-derived oxygen-deficient titania-mediated photodynamic/photothermal-enhanced immunotherapy for tumor treatment. ACS Appl. Mater. Interfaces.

[148] Thiruppathi J, Vijayan V, Park I-K, Lee S E, Rhee J H (2024). Enhancing cancer immunotherapy with photodynamic therapy and nanoparticle: making tumor microenvironment hotter to make immunotherapeutic work better. Front. Immunol.

[149] Lima-Sousa R, Melo B L, Alves C G, Moreira A F, Mendonça A G, Correia I J (2021). Combining photothermal-photodynamic therapy mediated by nanomaterials with immune checkpoint blockade for metastatic cancer treatment and creation of immune memory. Adv. Funct. Mater.

[150] Zhao R, Li S, Zhao J, Yao C (2025). Advancements in nano-delivery systems for photodynamic and photothermal therapy to induce immunogenic cell death in tumor immunotherapy. Int. J. Nanomed.

[151] Jin F, Liu D, Xu X, Ji J, Du Y (2021). Nanomaterials-based photodynamic therapy with combined treatment improves antitumor efficacy through boosting immunogenic cell death. Int. J. Nanomed.

[152] Sun Q, Yang J, Wu Q, Shen W, Yang Y, Yin D (2023). Targeting lysosome for enhanced cancer photodynamic/photothermal therapy in a “one stone two birds” pattern. ACS Appl. Mater. Interfaces.

[153] Lu J, Wang Q, Du J, Wu S, Miao Q, Li Y (2024). Bismuth-based porphyrin metal organic framework specific nano-inducer for tumor photoimmunotherapy. Chem. Eng. J.

[154] Guan X, Zhang X, Wang X (2025). Synergistic nanotherapy enhances tumor and lymph node immunity. Chem.

[155] Meng X, Che C, Yi Y, Qu X (2025). Reprogramming the tumor-immune landscape via nanomaterial-induced immunogenic cell death: a mini review. Front. Bioeng. Biotechnol.

[156] Ma R, Yang Z, Miao X, Hu J, Zhang T, Ma L (2025). Dual-mode radiosensitization of esophageal squamous cell carcinoma via SOCS6-loaded virus-inspired manganese-bismuth bimetallic oxide nanoparticles. Adv. Healthcare Mater.

[157] Chen Z, He Q, Qian R, Cai J, Wang Z, Zhou X (2025). Reengineering the tumor oxygen microenvironment in radiotherapy to enhance T-Cell function for radio-immunotherapy. Adv. Funct. Mater.

[158] Luo H, Ma W, Chen Q, Yang Z, Dai Y (2023). Radiotherapy-activated tumor immune microenvironment: Realizing radiotherapy-immunity combination therapy strategies. Nano Today.

[159] Liu S, Wang W, Hu S, Jia B, Tuo B, Sun H (2023). Radiotherapy remodels the tumor microenvironment for enhancing immunotherapeutic sensitivity. Cell Death Dis.

[160] Tian L, Lin M, Zhong H, Cai Y, Li B, Xiao Z (2022). Nanodrug regulates lactic acid metabolism to reprogram the immunosuppressive tumor microenvironment for enhanced cancer immunotherapy. Biomater. Sci.

[161] Piao H, Jiang Y, Jin S, Shi J, Yu J, Wang W (2025). Modulation of the immune microenvironment using nanomaterials: a new strategy for tumor immunotherapy. Front. Immunol.

[162] Luo Y, Li C, Zhang Y, Liu P, Chen H, Zhao Z (2023). Gradient tumor microenvironment-promoted penetrating micelles for hypoxia relief and immunosuppression reversion in pancreatic cancer treatment. Acta Biomater.

[163] Liu L, Pan Y, Zhao C, Huang P, Chen X, Rao L (2023). Boosting checkpoint immunotherapy with biomaterials. ACS Nano.

[164] Wang X, Wang C, Tian H, Wu B, Cheng W (2023). Tumor microenvironment-related sonodynamic therapy mediates tumor therapy. Adv. Ther.

[165] Dong C, Yi Q, Fang W, Zhang J (2022). A mini review of nanomaterials on photodynamic therapy. J. Photochem. Photobiol. C.

[166] Hu H, Zhao J, Ma K, Wang J, Wang X, Mao T (2023). Sonodynamic therapy combined with phototherapy: Novel synergistic strategy with superior efficacy for antitumor and antiinfection therapy. J. Control. Release.

[167] Son S, Kim J H, Wang X, Zhang C, Yoon S A, Shin J (2020). Multifunctional sonosensitizers in sonodynamic cancer therapy. Chem. Soc. Rev.

[168] Chen R, Yang Z, Chen Y, Wang J, Wang Y, Wei L (2025). Bismuth sulfide-titania heterostructure used for photothermal amplified sonodynamic therapy against hypoxic tumor. Mater. Today Bio.

[169] Gui M, Zheng X, Fu H, Cang M, Ma H, He J (2025). Applications of sonodynamic therapy combined with nanobiotechnology in the treatment of digestive tract tumors. Discov. Oncol.

[170] Xue Y, Wang Q, Chen Y, Zhang X, Tang J, Liu Y (2025). Biomimetic diselenide-sonosensitizer nanoplatform for enhanced sonodynamic therapy and in situ remodeling immunosuppressive microenvironment via activating innate and adaptive immunotherapy. Adv. Healthc. Mater.

[171] Yu Y, Song Z, Zhu A, Li J, Fan R, Xiao B (2025). Sequential tumor microenvironment reprogramming by nanoplatform potentiates sonodynamic-chemodynamic therapy and immune checkpoint blockade in breast cancer. Adv. Sci.

[172] Zhang A, Meng K, Liu Y, Pan Y, Qu W, Chen D (2020). Absorption, distribution, metabolism, and excretion of nanocarriers in vivo and their influences. Adv. Colloid Interface Sci.

[173] Yang H, Lu D, Liu Z, Xu Y, Niu Y, Liu C (2024). pH-responsive nanozyme cascade catalysis: A strategy of BiVO4 application for modulation of pathological wound microenvironment. J. Colloid Interface Sci.

[174] Ji Y, Wang Y, Wang X, Lv C, Zhou Q, Jiang G (2024). Beyond the promise: Exploring the complex interactions of nanoparticles within biological systems. J. Hazard. Mater.

[175] Wang Y, He X, Huang K, Cheng N (2024). Nanozyme as a rising star for metabolic disease management. J. Nanobiotechnol.

[176] Chen X, Sun D, He Z, Kang S, Miao Y, Li Y (2024). Ferrite bismuth-based nanomaterials: From ferroelectric and piezoelectric properties to nanomedicine applications. Colloids Surf, B.

[177] Chen M, Hei J, Huang Y, Liu X, Huang Y (2024). In vivo safety evaluation method for nanomaterials for cancer therapy. Clin. Transl. Oncol.

[178] Mu L, Yu F, Jia Y, Sun S, Li X, Zhang X (2023). Machine Learning in Prediction of Nanotoxicology. In: Hong, H. (eds) Machine Learning and Deep Learning in Computational Toxicology. Computational Methods in Engineering & the Sciences. Springer, Cham.

